# Histone deacetylases 1 and 2 maintain S-phase chromatin and DNA replication fork progression

**DOI:** 10.1186/1756-8935-6-27

**Published:** 2013-08-15

**Authors:** Srividya Bhaskara, Vincent Jacques, James R Rusche, Eric N Olson, Bradley R Cairns, Mahesh B Chandrasekharan

**Affiliations:** 1 Department of Radiation Oncology, University of Utah School of Medicine, Salt Lake City 84112, UT, USA; 2 Department of Oncological Sciences, University of Utah School of Medicine, Salt Lake City 84112, UT, USA; 3 Huntsman Cancer Institute, University of Utah School of Medicine, 2000 Circle of Hope, Salt Lake City 84112, UT, USA; 4 Repligen Corporation, Waltham 02453, MA, USA; 5 Department of Molecular Biology, University of Texas Southwestern Medical Center, Dallas 75390, TX, USA

**Keywords:** Hdacs1, Hdacs2, Replication, Nascent chromatin, Chromatin remodelers

## Abstract

**Background:**

Histone deacetylases (HDACs) play a critical role in the maintenance of genome stability. Class I HDACs, histone deacetylase 1 and 2 (Hdac1 and Hdac2) are recruited to the replication fork by virtue of their interactions with the replication machinery. However, functions for Hdac1 and Hdac2 (Hdacs1,2) in DNA replication are not fully understood.

**Results:**

Using genetic knockdown systems and novel Hdacs1,2-selective inhibitors, we found that loss of Hdacs1,2 leads to a reduction in the replication fork velocity, and an increase in replication stress response culminating in DNA damage. These observed defects are due to a direct role for Hdacs1,2 in DNA replication, as transcription of genes involved in replication was not affected in the absence of Hdacs1,2. We found that loss of Hdacs1,2 functions increases histone acetylation (ac) on chromatin in S-phase cells and affects nascent chromatin structure, as evidenced by the altered sensitivity of newly synthesized DNA to nuclease digestion. Specifically, H4K16ac, a histone modification involved in chromatin decompaction, is increased on nascent chromatin upon abolishing Hdacs1,2 activities. It was previously shown that H4K16ac interferes with the functions of SMARCA5, an ATP-dependent ISWI family chromatin remodeler. We found SMARCA5 also associates with nascent DNA and loss of SMARCA5 decreases replication fork velocity similar to the loss or inhibition of Hdacs1,2.

**Conclusions:**

Our studies reveal important roles for Hdacs1,2 in nascent chromatin structure maintenance and regulation of SMARCA5 chromatin-remodeler function, which together are required for proper replication fork progression and genome stability in S-phase.

## Background

Histone deacetylase inhibitors (HDAC inhibitors/HDIs) are potent anticancer drugs. Several broad-spectrum inhibitors are in various stages of clinical trials for both solid tumors and hematopoietic malignancies [1]. Two of these compounds (SAHA/Vorinostat and Depsipeptide/Romidepsin) have gained FDA approval for their use against T-cell cutaneous lymphomas. SAHA and Depsipeptide target class I HDACs (Hdacs 1, 2, 3 and 8) [2]. Therefore, it is imperative to study and understand the specific functions of individual HDACs in order to ascertain drug specificity and design targeted therapeutics with increased potency and minimal side effects.

Hdac1 and Hdac2 are the core enzymes in three distinct protein complexes (Sin3, NURD and CoREST) that have diverse cellular functions [3]. Targeted deletion of Hdac1 led to embryonic lethality [4]. *Hdac2*-null pups die within a month due to cardiac defects and abnormalities in myocyte proliferation [5]. Knockout of either Hdac1 or Hdac2 had minimal effect on hematopoiesis and on the cell cycle, likely due to compensation for one by the other, as they are highly similar proteins. However, deletion of both genes dramatically impaired proliferation in multiple cell types by blocking cells at the G1 to S phase transition [6,7]. Additionally, double knockout of these enzymes caused mitotic catastrophe in fibrosarcoma cells [8]. While a role for HDACs in transcription is well established [9-11], these enzymes also function in DNA replication. Hdac1 interacts with proliferating cell nuclear antigen (PCNA, a DNA replication processivity factor) [12]. Also, Hdacs1,2 associate with newly replicated DNA [13]. However, the precise functions for Hdacs1,2 in DNA replication are still not fully understood. Broad-spectrum (‘pan’) HDAC inhibitors inhibit cell cycle progression and kill cancer cells by triggering DNA damage during DNA replication/S-phase [14]. Hence, it is important to understand how Hdacs1,2 function during replication and how these HDACs affect nascent chromatin.

In this study, we aimed to understand the mechanistic roles for Hdacs1,2 in DNA replication. Using novel selective inhibitors and genetic knockdown systems, we show that Hdacs1,2 functions are required for the proper progression of the DNA replication fork, and loss of Hdacs1,2 activities leads to the activation of replication stress and DNA damage response. This defect in replication is not simply caused by changes in transcription, as gene expression for factors involved in replication remain unchanged following Hdacs1,2-inhibitor treatment. Mechanistically, the defect in replication in the absence of Hdacs1,2 functions can be attributed to an altered chromatin structure, due to increased histone acetylation on S-phase chromatin, especially increased H4K12ac and H4K16ac levels. H4K12ac and H4K16ac antagonize substrate recognition and nucleosome remodeling activity of SMARCA5, an ISWI family chromatin remodeler [15,16]. In this study, we further show that SMARCA5 is present on nascent chromatin, and loss of SMARCA5 also leads to a decrease in the replication fork velocity and activation of the replication stress response. This demonstrates an important role for SMARCA5 in replication fork progression in mammalian cells. Overall, we provide a model wherein Hdacs1,2 affect DNA replication fork progression by regulating histone acetylation on nascent chromatin and SMARCA5 activity.

## Results

### Abrogating histone deacetylase 1 and 2 activities increases replication-associated histone deposition marks

**Figure 1:**
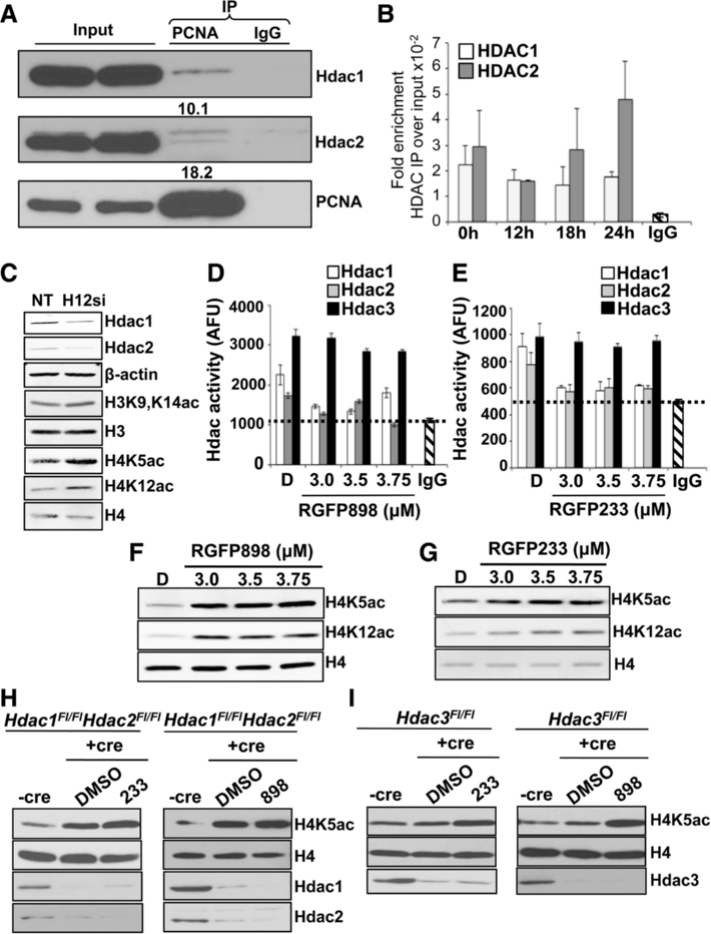
**Histone deacetylase 1 and 2 (Hdacs1,2) localize to replication origins and target histone deposition marks. A**. Western blot analysis of immunoprecipitated (IP) samples to determine interaction of Hdac1 and Hdac2 with proliferating cell nuclear antigen (PCNA). Nuclear extracts from HeLa cells were used in IP with anti-PCNA antibody or IgG (negative control). Percentage ratio of IP over input for Hdac1 and Hdac2 are shown. **B**. Hdac1 and Hdac2 occupancies at *α*-*globin* locus (early replicating origin) in NIH3T3 cells synchronized in S-phase were determined using chromatin immunoprecipitation (ChIP) assays. Average fold enrichment of immunoprecipitated DNA over input DNA from three independent experiments is shown. **C**. Western analysis of nuclear extracts prepared from NIH3T3 cells transfected with either non-targeting (NT) or Hdac1,2 siRNA (H12si) at 72 h post-siRNA transfection to determine changes in histone modifications. **D-E**. Hdac1 or Hdac2 or Hdac3 immunoprecipitated from nuclear extracts following treatment of NIH3T3 cells with either DMSO (D), 898 (panel **D**) or 233 (panel **E**) were used in enzyme assays. Enzyme inhibition was measured using Fluor-de-Lys HDAC fluorimetric activity assay. AFU, arbitrary fluorescence units; *dotted line*, denotes background/baseline signal obtained from using rabbit IgG in IP. **F-G**. Western analysis to determine changes in histone acetylation in NIH3T3 cells following 24 h treatment with DMSO or increasing concentrations of 898 **(F)** or 233 **(G)**. **H-I**. Western analysis of whole cell lysates prepared from *Hdac1*^*Fl*/*Fl*^*Hdac2*^*Fl*/*Fl*^ or *Hdac3*^*Fl*/*Fl*^ fibrosarcoma cells following Ad-Cre infection and treatment with 898 or 233. Cells were treated with 3 μM 898 or 233 for 24 h following a 48 hr Ad-Cre infection.

Both Hdac1 and Hdac2 localize to sites of DNA replication [13]. In HEK293 cells, Hdac1 interacts with PCNA, the replication-sliding clamp [12]. We sought to test whether Hdac2 also interacts with PCNA in human cells using co-immunoprecipitation. Hence, we used human HeLa cell extracts for this analysis. Indeed, we find that both Hdac1 and Hdac2 co-immunoprecipitate with PCNA (Figure 1A). We next examined whether Hdacs1,2 associate with replication origins in cells synchronized in S-phase. Given the efficiency of cell synchronization by serum-starvation and to obviate the need to use any chemical cell cycle blocking agents, we employed NIH3T3 cells for further experiments. NIH3T3 cells were serum-starved to arrest cells in the G0/G1 phase of the cell cycle. Cells were then released into S-phase by growing them in a serum-rich medium for various time points (12 h, 18 h, 24 h). Using chromatin immunoprecipitation (ChIP) assays, we found that Hdac1 and Hdac2 are enriched at candidate early (*α-globin*), mid-late (*pancreatic amylase*) and late (*β-globin*) replicating loci in cells synchronized in S-phase [17] (Figure 1B and Additional file 1: Figures S1A and S1B). Collectively, our findings confirm that Hdacs1,2 interact with PCNA and localize to sites of DNA replication. Newly synthesized histones are acetylated on histone H4 K5 and K12 residues prior to their deposition onto nascent chromatin, and are then removed during chromatin maturation [18]. One function for Hdacs1,2 during DNA replication might be to deacetylate these histone deposition marks. In primary mouse embryo fibroblasts (MEFs), deletion of Hdac1 and 2 leads to an increase in H4K5ac and H4K12ac [7]. We examined the global levels of H4K5ac and H4K12ac in nuclear extracts prepared from cells following siRNA-mediated knockdown of Hdacs1,2 in NIH3T3 cells. We also examined H3K9,K14ac, a mark associated with transcription [19,20]. Knockdown of both Hdacs1,2 led to an increase in H4K5ac, H4K12ac and H3K9,K14ac in NIH3T3 cells when compared to the control cells transfected with non-targeting siRNA (Figure 1C). In corroboration with previous studies in primary cells [7], deletion of Hdac1 alone or knockdown of Hdac2 alone in fibrosarcoma cells did not result in any increase in H4K5ac (Additional file 2: Figure S2). Therefore, both Hdac1 and Hdac2 target the histone deposition marks.

To further examine if histone acetylation marks increase upon inhibition of Hdacs1,2 activities, we chose to selectively inactivate these two enzymes using novel, benzymilic class small molecule inhibitors (RGFP898 and RGFP233, henceforth referred to as 898 and 233, respectively). We first determined the selectivity of these two molecules towards Hdacs1,2. The IC50 values obtained using *in vitro* HDAC assays showed 233 and 898 inhibit Hdacs1,2 activities at a low concentration (Additional file 3: Figure S3A). Unlike SAHA, the inhibitory activity of RGFP106 (another benzamide-type inhibitor similar to 898 or 233) was previously shown to remain unchanged even after 100-fold dilution of the inhibitor-enzyme mixture and histone acetylation did not return to basal levels even after washing away the inhibitor [21]. Therefore, these benzamide-type Hdacs1,2 inhibitors are slow and tight-binding compounds. We next examined the efficacy of 898 and 233 to inhibit Hdacs1,2 in NIH3T3 cells. An increase in histone acetylation was observed following treatment of NIH3T3 cells with 2 to 10 μM 898 (Additional file 3: Figure S3B). We then determined the minimum concentration range required to inhibit Hdac1,2 activities and to increase histone acetylation in NIH3T3 cells. A robust inhibition of only Hdacs1,2 activities was observed at lower concentrations of 898 or 233 (3.0 to 3.75 μM) (Figure 1D, 1E). To ensure the reduced enzyme activity is not due to differences in the enzyme concentrations used in the assay, we checked and confirmed that, indeed, equal amount of Hdac1, Hdac2 and Hdac3 were present in the immunoprecipitates (Additional file 4: Figure S4). Collectively, these characterization studies confirmed the efficacy of 898 and 233 as Hdac1,2-selective inhibitors, and provided us the minimal, effective concentration range for these two inhibitors to be used in our studies (3 to 3.75 μM).

Similar to the knockdown of Hdacs1,2 (Figure 1C), inhibition of Hdacs1,2 *in vivo* using the selective inhibitors (898 or 233) also resulted in an increase in H4K5ac, H4K12ac and H3K9,K14ac levels when compared to cells treated with vehicle alone (DMSO) (Figure 1F-G). Given their high sequence homology [22,23], we sought to further confirm the specificity of 233 and 898 towards only Hdacs1,2 and not Hdac3. To this end, we used fibrosarcoma cells containing floxed alleles of either Hdac1 and Hdac2 (*Hdac1*^*Fl*/*Fl*^*Hdac2*^*Fl*/*Fl*^) or Hdac3 (*Hdac3*^*Fl*/*Fl*^) to obtain conditional knockout of these enzymes upon expressing Cre recombinase [8]. Efficient depletion of Hdacs1,2 and Hdac3 were observed in these cells following infection with an adenovirus-containing Cre recombinase (Ad-Cre) (Figure 1H and 1I). Conditional deletion of Hdacs1,2 in fibrosarcoma cells led to a significant increase in H4K5ac (Figure 1H), whereas deletion of Hdac3 led to a subtle increase in H4K5ac (Figure 1I). Treatment of *Hdac1*,*2* knockout cells with 233 or 898 did not result in any further increase in H4K5ac (Figure 1H, Additional file 5: Figure S5A and S5B), confirming that these two inhibitors are selective for Hdacs1,2. Addition of 233 or 898 to *Hdac3* knockout cells resulted in a significant increase in H4K5ac (Figure 1I). This increase in H4K5ac is an additive effect obtained due to the inhibition of Hdacs1,2 activities by these two molecules combined with the loss of Hdac3 activity (Figure 1I and Additional file 5: Figures S5C and S5D). Taken together, our studies using genetic systems and selective inhibitors reveal a role for Hdacs1,2 in the removal of histone deposition marks.

### Inhibition of histone deacetylase 1 and 2 activities does not affect the progression of cells through S-phase, but decreases bromodeoxyuridine incorporation

**Figure 2:**
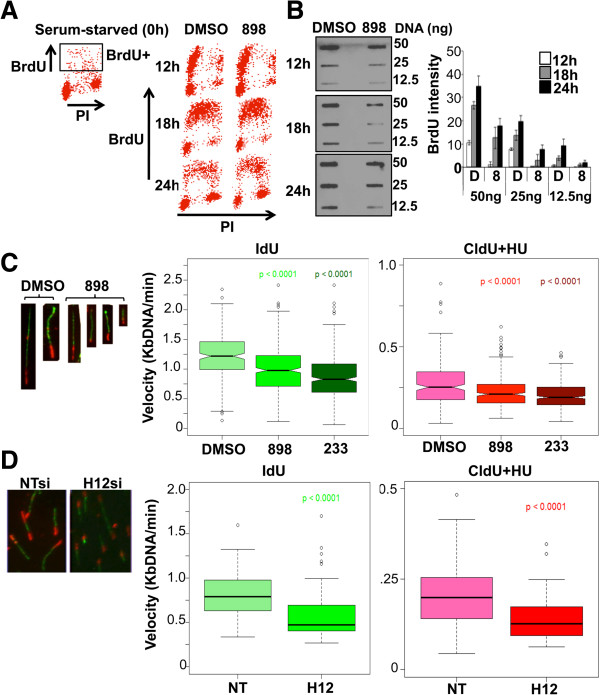
**Inhibition of histone deacetylase 1 and 2 activities within S-phase or their knockdown reduces replication fork velocity. A**. Serum-starved NIH3T3 cells were released into S-phase in the presence of DMSO or 3 μM 898. Fluorescence-activated cell sorting analysis following bromodeoxyuridine (BrdU)-propidium iodide labeling and staining was performed at 0 h, 12 h, 18 h and 24 h following release into S-phase. **B**. Slot blot analysis was performed using the indicated amount of BrdU-labeled genomic DNA from DMSO or 3 μM 898 treated cells. BrdU incorporation was quantitated using densitometry. **C**. Fork velocity was measured by DNA combing. After 20 h following release into S-phase in the presence of DMSO or 3 μM 898, NIH3T3 cells were labeled with IdU (green) for 15 min and then with CldU (red) in the presence of 250 μM hydroxyurea for 20 min. DNA fibers were prepared and analyzed as described (Methods section). Box plots show average fork velocity of DNA fibers prepared from four independent 898 or 233 treatments. At least 100 fibers in different areas of the slide from three different slides were analyzed per experiment. **D**. Fork velocity was measured in NIH3T3 cells transfected with non-targeting (NT) or Hdacs1,2 (H12) siRNA. Box plots show average fork velocity from two independent experiments. The ‘box’ in each box plot (C-D) extends from the first to third quartile of the data, heavy dark line inside the box represents the median, and the ‘whiskers’ extend to data points no more than 1.5 times the interquartile range from the ends of the box. Open circles represent data points further from the ends of the box than 1.5 times the interquartile range. Two sided *P* values were determined using Welch’s two-sample *t*-test. R statistical computing software (version 2.15.0) was used to construct box plots and for *t*-tests.

Deletion of both Hdac1 and Hdac2 in primary mouse embryo fibroblasts using a tamoxifen-inducible conditional knockout system resulted in G1 arrest and a dramatic decrease in BrdU incorporation, as cells failed to enter and progress through the S-phase [6,7]. However, these phenotypes are evident only following progression of knockout cells through a few rounds of the cell cycle [6,7]. The G1 arrest caused upon abrogation of Hdacs1,2 functions has restricted studying the functions for these two enzymes within S-phase and in DNA replication. However, treatment of NIH3T3 cells with 898 or 233 did not arrest cells in G1 phase following 24 h treatment (Additional file 6: Figure S6A-B). Therefore, we serum-starved cells to induce G0/G1 arrest. Cells were then released into S-phase by growing them in a serum-rich medium supplemented with either vehicle (DMSO) or the Hdacs1,2-selective inhibitor (898 or 233) and treated for various time (12 h, 18 h, 24 h). S-phase cells were measured by fluorescence-activated cell sorting (FACS) analysis following BrdU and propidium iodide staining. We did not observe any accumulation of cells, indicative of a block within S-phase, at the three different time points of treatment with 898 (Figure 2A). We did not observe G1 arrest in NIH3T3 cells transfected with Hdacs1,2 siRNAs at 72 h post-transfection, even though we observed increased histone acetylation at this time point (Additional file 6: Figure S6C). Additionally, treatment with 3 μM 898 or 233 did not affect the progression of cells from G1 to early S-phase (12 h post-release), early to mid S-phase (18 h post-release) and mid- to late S-phase-G2/M (24 h post-release) (Figure 2A and Additional file 7: Figure S7). Continuous inhibition of Hdac1,2 activities with 898 for 48 h in NIH3T3 cells led to a G1 arrest of cells and decrease in S-phase population (Additional file 8: Figure S8). Hence, the availability of small molecule inhibitors (898 or 233) and treatment of cells for short duration (12 to 24 h) allowed us to selectively inhibit Hdacs1,2 within S-phase cells and test whether inhibition of Hdacs1,2 affects progression of cells through the S-phase. Importantly, these findings provided us a window to study Hdacs1,2 functions within S-phase following their inhibition using a selective inhibitor (12 to 24 h) or the knockdown system (72 h).

To examine if Hdacs1,2 activities are required for DNA replication, we released serum-starved cells into S-phase and treated them for 12 h, 18 h or 24 h with either the vehicle (DMSO) or the Hdac1,2-selective inhibitor (898 or 233). Nascent DNA was labeled with BrdU prior to harvesting. Changes in BrdU incorporation were assessed by slot blot analysis using equal amounts of genomic DNA isolated from DMSO-, 898- or 233-treated cells. We observed a two-fold reduction in BrdU incorporation in cells treated with the 898 or 233 when compared to the untreated control cells (Figure 2B and Additional file 9: Figure S9). Importantly, this finding suggests that Hdacs1,2 activities are required for efficient synthesis of nascent DNA during DNA replication.

### Abrogating histone deacetylase 1 and 2 functions affects replication fork velocity and activates the replication stress response

Defective DNA replication in the absence of Hdacs1,2 activities might be a cause for the reduced BrdU incorporation in 898- or 233-treated S-phase cells (Figure 2B and Additional file 9: Figure S9). To test this possibility, we used the molecular combing assay to examine if loss or inhibition of Hdacs1,2 affects replication fork progression. In the combing assay, cells are sequentially pulse-labeled with two different halogenated thymidine analogs (IdU, iodo-deoxyuridine and CldU, choloro-deoxyuridine) to independently mark initiation/early elongation events and subsequent progression of the fork at replicating origins. After spreading the DNA fibers on a slide (combing), newly replicated regions are detected using fluorescent dye-labeled antibodies that specifically recognize the two different incorporated thymidine analogs. Length of the fluorescence signal and the labeling time are then used to calculate the replication fork velocity (Kb/min). In this assay, defects in replication fork progression or elongation can be further exacerbated via stalling the fork using a dose of hydroxyurea (HU, a ribonucleotide reductase inhibitor) that does not cause fork collapse [24].

To measure changes, if any, in the replication fork velocity upon abrogation of Hdacs1,2 activities, we released serum-starved cells into S-phase and treated them with DMSO (vehicle) or with the Hdacs1,2-selective inhibitor (898 or 233) followed by pulse labeling of nascent DNA with IdU (Figure 2C). After removing any free IdU, we performed the second pulse labeling with CldU in the presence of HU. We then measured the length of the two pulse-labels to obtain replication fork velocities, which were further classified into categories based on the distance travelled by the fork. We performed box plot analysis to measure the average fork velocity (Figure 2C, 2D). We also classified fibers into bins of increasing velocities to examine if loss of Hdacs1,2 activities affects fibers of a particular velocity range (Additional file 10: Figure S10A). With IdU labeling, replication fork velocities were reduced in the presence of Hdacs1,2-selective inhibitor (898 or 233) compared to the control (Figure 2C and Additional file 10: Figure S10A, see 0.95 to 1.6 Kb/min). Defects in replication fork progression due to inhibition of Hdacs1,2 were also evident for CldU labeling in the presence of hydroxyurea (HU), which slows/stalls fork progression (Figure 2C and Additional file 10: Figure S10A). In addition, we observed a severe reduction in the fork velocity upon treatment of NIH3T3 cells with SAHA that inhibits Hdacs1, 2 and 3 (Additional file 11: Figure S11). We further measured the replication fork velocities in NIH3T3 cells following knockdown of Hdacs1,2. Loss of Hdacs1,2 caused a decrease in fork velocity (Figure 2D, IdU label and Additional file 10: Figure S10B), which was further affected in the presence of hydroxyurea (Figure 2D, CldU label). This decrease in fork velocities found upon loss of Hdacs1,2 correlates well with that observed upon preventing their activities using selective inhibitors (Figure 2C). Collectively, our findings demonstrate that Hdac1,2 functions are required for maintaining normal replication fork rates.

**Figure 3:**
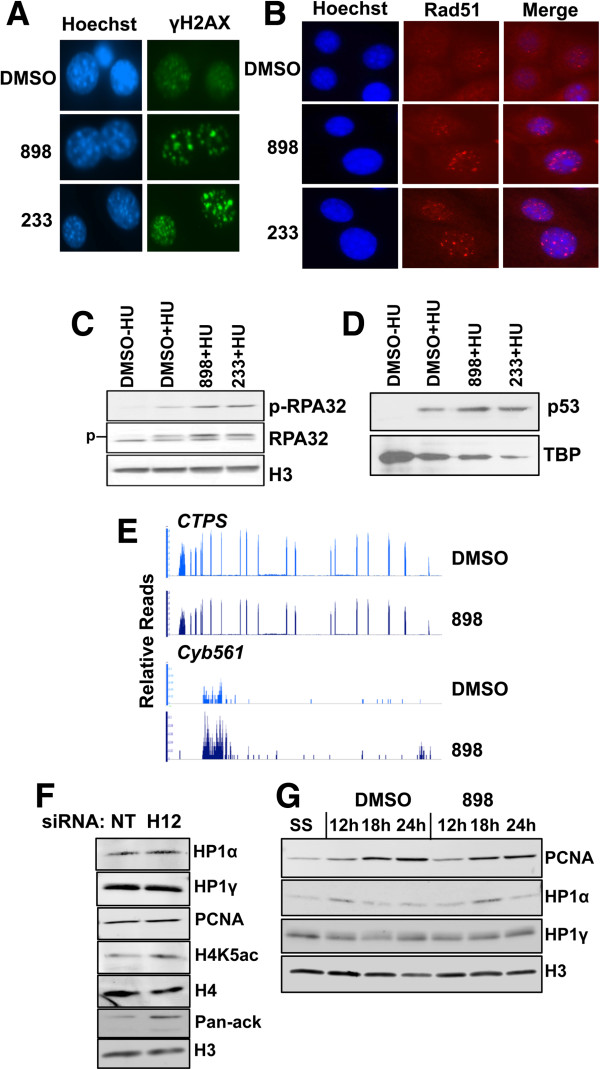
**Abolishing histone deacetylase 1 and 2 (Hdacs1,2) activities causes DNA damage and replication stress, but transcription of genes for replication factors is not affected. A**. NIH3T3 cells were treated with either DMSO or 3 μM 898 or 233 for 48 h and immunofluorescence with anti-γH2AX antibody was performed to examine activation of DNA damage response. **B**. Immunofluorescence using anti-Rad51 antibody was performed on serum-starved NIH3T3 cells following a 20 h release into S-phase and treatment with DMSO or 3 μM 898 or 3 μM 233 in the presence of 500 μM HU. A representative image from multiple treatments is shown. **C-D**. HeLa cells were treated with 3 μM 898 or 233 in the presence of 500 μM hydroxyurea for 48 h prior to chromatin preparation. Western analysis of chromatin was done to determine changes in RPA32, modified form of RPA32 (RPA32 S4/S8 phosphorylation, p-RPA32) and p53. *p*, phosphorylated and slow migrating species of RPA32. H3 and TBP (TATA binding protein) serve as loading controls. **E**. Total RNA was isolated from NIH3T3 cells released into S-phase following serum starvation and treated for 20 h with either DMSO or 3 μM 898. RNA samples obtained from three independent DMSO- or 898-treated cells were subjected to sequencing using the Illumina Hiseq2000 sequencer. Relative reads for *CTP synthetase* (*CTPS*) and *Cyb561* genes in control and treated samples are shown. **F**. Chromatin was prepared from non-targeting (NT) or Hdacs1,2 (H12) siRNA transfected cells at 72 h time point for western analyses. H3 and H4 serve as loading controls. **G**. Serum-starved NIH3T3 cells were released into S-phase in the presence of DMSO or 3 μM 898. Chromatin extracts were prepared at 0 h (SS, serum-starved), 12 h, 18 h and 24 h following release for western analyses.

If stalled forks are not restarted in a timely fashion, it could result in fork collapse, formation of double strand breaks and activation of the DNA damage response, which involves recruitment of ATM and ATR (damage-activated sensor kinases) to break sites and activation of the intra-S-phase checkpoint [25,26]. To examine if inhibition of Hdacs1,2 activities triggers a DNA damage response, we performed immunofluorescence analysis to detect γH2AX (phosphorylated form of H2AX and a marker of DNA damage [25]) following treatment of cells with 898 or 233. We observed an increased number of γH2AX foci, and brighter foci, in 898- or 233-treated cells compared to the untreated control cells (Figure 3A, Additional file 12: Figure S12). In addition to replication stress-induced breaks, loss of Hdacs1,2 also impairs double-strand DNA break repair [27]. The observed DNA damage response following inhibition of Hdacs1,2 activities could be due to a cumulative effect of continuous replication stress response and also failure to repair breaks induced during S-phase. Knockdown of Hdacs1,2 also caused an increase in γH2AX foci in NIH3T3 cells at 72 h post-siRNA transfection (Additional file 13: Figure S13). In addition, treatment of wild-type primary mouse embryo fibroblasts with either 898 or 233 led to an increase in the percentage of cells with big and bright γH2AX foci (Additional file 14: Figure S14). Hence, loss of Hdacs1,2 activates DNA damage response in different cell types. During replication, the RecA homolog Rad51 foci formation is triggered when forks get inactivated and collapse under conditions of intense replication stress [28]. To examine if loss of Hdacs1,2 leads to collapsed forks, we performed immunofluorescence to detect Rad51 in S-phase cells in the presence of 898 or 233. To intensify the replication stress, we used hydroxyurea in addition to the Hdacs1,2-selective inhibitors. Under these treatment conditions, a significant increase in the percentage of cells with Rad51 foci was observed (Figure 3B and Additional file 15: Figure S15). Replication protein A (RPA) is a single-strand DNA binding, trimeric protein complex comprised of 14, 32 and 70 kDa subunits [29]. RPA has a well-characterized role in DNA replication, and RPA32 is phosphorylated by ATM/ATR kinases in response to replication stress, such as, hydroxyurea treatment [29]. We found the chromatin-associated levels of phospho-RPA32 induced by hydroxyurea treatment are further increased upon inhibition of Hdac1,2 activities with 898 or 233 treatment (Figure 3C). This finding correlates well with the reduced replication fork velocities observed upon abrogating Hdacs1,2 activities in the presence of hydroxyurea (Figure 2C, 2D). Moreover, it suggests that abolishing Hdacs1,2 activities adversely affects replication fork movement and causes replication stress. In addition, we found increased level of chromatin-associated p53 upon hydroxyurea treatment in cells treated with 898 or 233 compared to the DMSO-treated control cells (Figure 3D), further confirming the activation of DNA damage response in cells lacking Hdacs1,2 functions. We used HeLa cells to look at phoshorylated-RPA32 and p53 levels, as antibodies that recognize these antigens failed to work in mouse NIH3T3 cells. Collectively, reduced replication fork velocity and activation of replication stress response were observed upon inhibition or loss of Hdacs1,2, which confirm that these two enzymes are crucial for the efficient progression of the replication fork.

### Inhibiting histone deacetylase 1 and 2 increases histone acetylation on S-phase chromatin without drastically altering S-phase gene transcription

We next sought to explore the molecular mechanism(s) by which Hdacs1,2 might promote replication fork progression. Reduced fork velocity might be due to a shortage of cellular dNTP pool, as seen upon hydroxyurea treatment [30], or alternatively, due to a decrease in transcription of genes involved in nucleotide biosynthesis. Treatment of cells with trichostatin A (TSA), a pan-HDAC inhibitor, reduced fork velocity, due to its effect on pyrimidine biosynthesis. TSA treatment decreased the expression of *CTP synthetase 1* and *thymidylate synthetase* genes, which in turn reduced pyrimidine biosynthesis [31]. To examine whether reduced fork velocity in 898-treated cells is linked to defects in transcription, we used RNA-seq to determine differential gene expression in three independent DMSO or 898-treated S-phase cells. We observed differential expression of 70 genes, including upregulation of cytochrome *Cyb561* gene (Figure 3E, Additional file 16: Figure S16A, Additional file 17: Table S1). However, transcript levels for *CTP synthetase 1* gene were not affected in all three independently treated samples (Figure 3E). Also, expression of genes coding for factors involved in DNA replication or DNA damage response remained unchanged (Additional file 16: Figure S16B). Therefore, our gene-expression analysis suggests that the reduced fork velocity and DNA damage observed upon inhibition of Hdac1,2 activities are not likely due to altered gene transcription.

It is conceivable that Hdacs1,2 might target a non-histone protein(s) with an important role in DNA replication. Smc3, a subunit of the cohesin complex, regulates replication and is acetylated by Eco1 acetyl transferase [32]. To test whether loss or inhibition of Hdacs1,2 affects Smc3 acetylation, we used an antibody that specifically recognizes the acetylated form of Smc3. While Smc3ac levels on chromatin increased in S-phase cells, we did not observe any change in the levels of Smc3 or its acetylated form following treatment with 898 (Additional file 18: Figure S17A) or following siRNA-mediated knockdown of Hdacs1,2 (Additional file 18: Figure S17B). These results suggest that Hdacs1,2 are not involved in regulating Smc3 or its acetylation, and this agrees well with the recent finding that Hdac8 is involved in Smc3 deacetylation [33]. It is possible that Hdacs1,2 target some other non-histone protein(s) involved in DNA replication.

Heterochromatin protein 1 (HP1) has been linked to assembly and maintenance of heterochromatin and to DNA replication [34]. HP1 binds methylated H3K9 [35]. Since H3K9,K14ac is increased in the absence of Hdacs1,2 functions (Figure 1C, Additional file 3: Figure S3B), we tested whether Hdacs1,2 play a role in the chromatin binding of replication-associated forms of HP1 (that is, HP1α and HP1γ) [36] indirectly via their regulation of histone modifications. We found chromatin-bound levels of HP1α and HP1γ in 898-treated S-phase cells were not affected compared to the untreated control cells (Figure 3F). Similar results were obtained following knockdown of Hdacs1,2 (Figure 3G). These findings suggest that global heterochromatin is not affected upon transient inhibition of Hdacs1,2 activities, and rule out reduced HP1 binding to chromatin as a reason for the DNA replication defects observed upon abrogating Hdacs1,2 functions.

**Figure 4:**
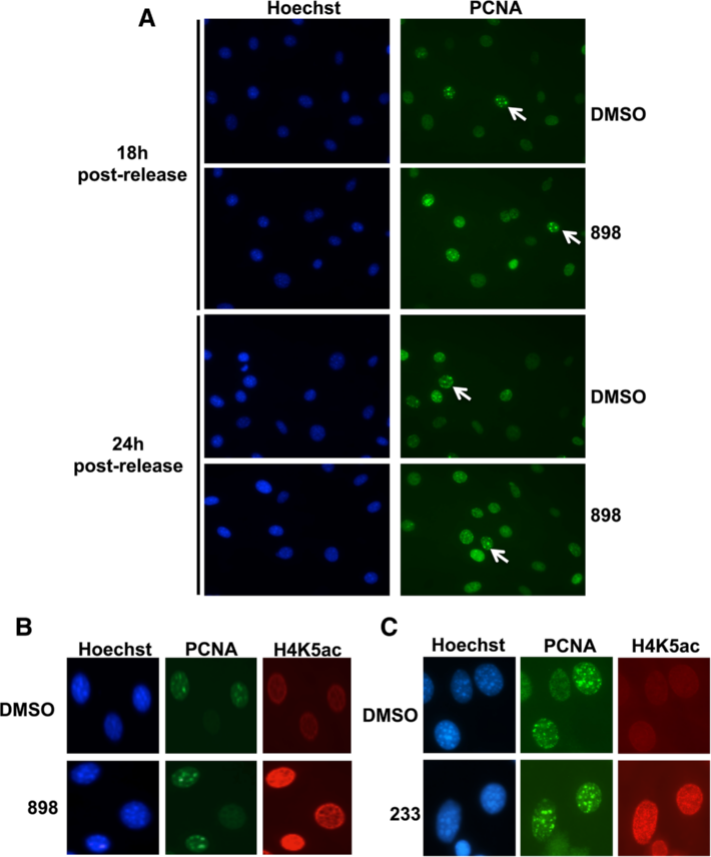
**Inhibition of histone deacetylase 1 and 2 (Hdac1,2) activities does not affect proliferating cell nuclear antigen (PCNA) localization but leads to increased H4K5ac in S-phase cells. A**. Serum-starved NIH3T3 cells were released into S-phase in the presence of DMSO or 3 μM 898 for 18 h or 24 h prior to staining of PCNA for immunofluorescence analysis. Punctate pattern of PCNA staining (arrows) indicative of replicating cells was seen in both DMSO and 898-treated cells. **B**. Serum-starved NIH3T3 cells were released into S-phase in the presence of DMSO or 3 μM 898 **(B)** or 233 **(C)** for 24 h and double immunofluorescence was performed using antibodies to stain PCNA and H4K5ac. Punctate pattern of PCNA staining was used to track cells in S-phase.

Hdacs1,2 interact with PCNA (Figure 1A) and Hdac1 deacetylates PCNA [37]. Hence, we tested if loss of Hdacs1,2 affects the chromatin association of PCNA due to its increased acetylation in S-phase. Western analysis of chromatin isolated from S-phase cells showed no defects in the recruitment of PCNA onto chromatin following treatment with 898 (Figure 3G). Given that PCNA moves with the fork [38], we performed immunofluorescence analysis to examine if the punctate pattern of PCNA foci, indicative of replication factories, is still seen in the absence of Hdac1,2 activities. We observed no defects in PCNA foci formation at both 18 h and 24 h following release from serum starvation in 898-treated S-phase cells (Figure 4A). Therefore, reduced fork velocity in the absence of Hdacs1,2 functions is not due to any change in PCNA loading onto chromatin during replication.

**Figure 5:**
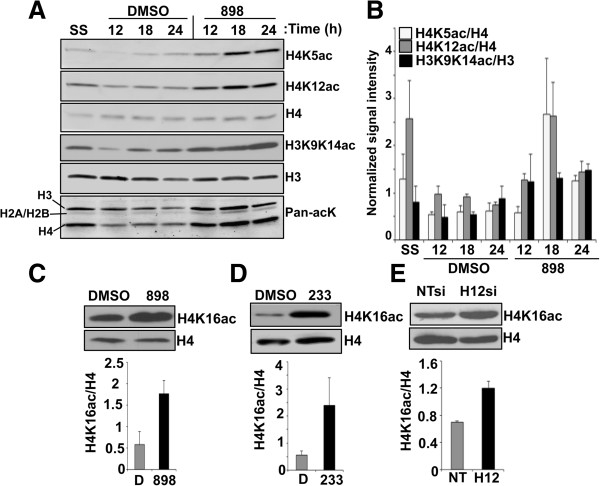
**Loss of histone deacetylase 1 and 2 (Hdacs1,2) function increases chromatin-associated histone acetylation. A**. Serum-starved (SS) NIH3T3 cells were released into S-phase in the presence of DMSO or 3 μM 898. Chromatin extracts were prepared at 0 h (SS), 12 h, 18 h, and 24 h following release into S-phase for western analyses to look at changes in histone acetylation. A representative blot from three independent experiments is shown. Pan-acK, anti-pan-acetyllysine antibody. **B**. Quantitation for histone acetylation shown in panel A. Average intensity for a histone acetylation normalized to the total histone level were calculated from three independent experiments. **C-D**. Western analysis of H4K16ac using chromatin extracts prepared from NIH3T3 cells treated with DMSO or 898 **(C)** or 233 **(D)** for 24 h. **E**. Western analysis of H4K16ac using chromatin extracts prepared from non-targeting or Hdacs1,2-siRNA transfected cells. H4 levels serve as loading control. For panels **C-E**, quantitation for H4K16ac levels normalized to total H4 level from three independent experiments is shown.

We then hypothesized that the stalling of the replication fork in the absence of Hdacs1,2 might be due to changes in the chromatin template, probably as a result of altered histone modifications. To test this possibility, we evaluated whether histone acetylation is increased on chromatin upon loss of Hdacs1,2 functions. As mentioned above, newly synthesized histone H4 is acetylated on K5 and K12 residues. Following their deposition onto nascent chromatin these histone marks are deacetylated, as part of the chromatin maturation process during replication. Chromatin maintenance during replication occurs by the concerted action of histone chaperones, which carry and deposit histones onto nascent DNA, and HDACs that remove these acetylation marks following their deposition [18]. Double immunofluorescence analysis showed a robust global increase in H4K5ac in cells with punctate PCNA foci following either 898 or 233 treatment (Figure 4B, 4C). This result suggests that Hdacs1,2 deacetylate H4K5 in S-phase cells. We further examined the levels of the histone deposition marks (H4K5ac and H4K12ac) and other histone acetylation marks on chromatin in S-phase cells following treatment with DMSO or 898. Indeed, H4K5ac, H4K12ac and H3K9,K14ac were all increased on chromatin in S-phase cells upon inhibition of Hdacs1,2 activities (Figure 5A, 5B). In addition to an increase in the acetylation at specific histone H3 or H4 residues, we found a general increase in the acetylation of all histones (H3, H4 and H2A or H2B) on chromatin in S-phase cells using an antibody that recognizes pan-acetyl lysine following inhibition of Hdacs1,2 activities (Figure 5A, Additional file 19: Figure S18). Addition of an acetyl group onto lysine residue neutralizes the positive charge. Therefore, histone acetylation can alter chromatin structure and packaging by affecting histone-DNA and histone-histone interactions. For instance, H4K16ac regulates the higher-order chromatin packaging, and knockdown of Hdacs1,2 increases H4K16ac in U2OS cells [27,39]. Hence, we tested whether H4K16ac levels increased upon inhibition of Hdacs1,2 activities. Indeed, chromatin-associated H4K16ac increased upon knockdown or inhibition of Hdacs1,2 in NIH3T3 cells (Figure 5C, 5D, 5E). Taken together, our findings show that Hdacs1,2 are required for maintaining histone acetylation levels on chromatin during S-phase. Furthermore, these results put forth a hypothesis that the stalling of replication forks in the absence of Hdacs1,2 activities might be due to an altered structure of the chromatin template.

### ISWI-family chromatin remodeler SMARCA5 associates with nascent DNA

**Figure 6:**
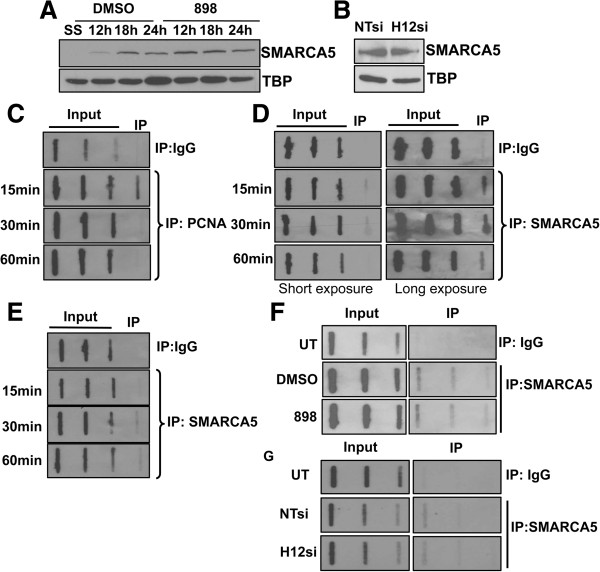
**SMARCA5 associates with nascent DNA. A**. Serum-starved NIH3T3 cells were released into S-phase in the presence of DMSO or 3 μM 898. Chromatin extracts were prepared at 0, 12 h, 18 h, and 24 h following release into S-phase for western analyses to look at SMARCA5 levels. **B**. Chromatin was prepared from either non-targeting (NT) siRNA or Hdac1,2 (H12) siRNA transfected cells at 72 h time-point to look at SMARCA5 levels. TATA binding protein (TBP) serves as a loading control. **C**. HeLa cells were labeled with bromodeoxyuridine (BrdU) for 30 min. Cells were then washed to remove unincorporated BrdU and cultured in media without BrdU for indicated periods of time (chase). Chromatin immunoprecipitation (ChIP) was performed with anti-proliferating cell nuclear antigen (PCNA) antibody. BrdU labeled DNA present in input DNA and those associated with PCNA were assessed in slot blot analysis using an anti-BrdU antibody. **D-E**. BrdU-pulse chase coupled to slot blot analysis in HeLa **(D)** or NIH3T3 **(E)** cells to determine the kinetics of SMARCA5 association to nascent DNA at the indicated chase time points. **F-G**. NIH3T3 cells were treated with either DMSO or 898 for 24 h or transfected with non-targeting or Hdac1,2 siRNA and labeled with 20 μM BrdU for 1 h before harvesting. ChIP of SMARCA5 or rabbit IgG control was done and increasing volumes of ChIP DNA were spotted onto a membrane using slot blot and probed with anti-BrdU antibody. In F-G, a representative blot from three independent experiments is shown. UT refers to untreated.

**Figure 7:**
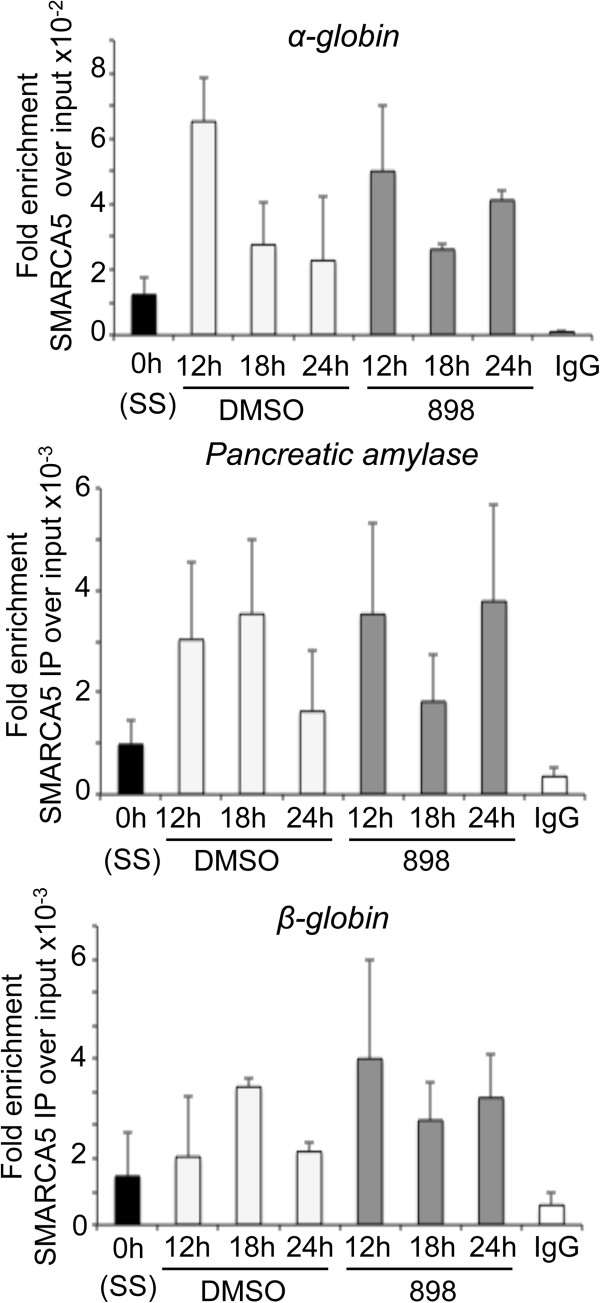
**SMARCA5 associates with candidate replication origins during S-phase.** Serum-starved (SS, 0 h) NIH3T3 cells were released into S-phase in the presence of DMSO or 3 μM 898. SMARCA5 occupancy at *α-globin* (early replicating origin), pancreatic amylase (mid-late replicating) and *β-globin* (late replicating) in synchronous NIH3T3 cells were determined using chromatin immunoprecipitation (ChIP) assays. Average fold enrichment for SMARCA5 relative to input from two independent experiments is shown.

In addition to histone modifications, nascent chromatin structure can also be modulated by the action of ATP-dependent chromatin remodeling enzymes. Indeed, chromatin-remodeling enzymes are recruited to replication sites, and are important for repositioning nucleosomes during DNA replication [40,41]. One such chromatin remodeler, ISWI/SNF2H/SMARCA5, is targeted to heterochromatic replication foci as part of the WICH chromatin-remodeling complex [42]. Additionally, SMARCA5 interacts with Hdac2, and a SMARCA5-Hdac1,2 complex has been proposed to play a role in the initiation of DNA replication at origins [43]. Therefore, we hypothesized that loss or inhibition of Hdacs1,2 might affect SMARCA5 recruitment and/or activity at the replication forks, which in turn might adversely affect nascent chromatin structure and replication fork progression. SMARCA5 levels on chromatin increased in S-phase cells, but no dramatic changes were observed following inhibition of Hdacs1,2 activities using 898 (Figure 6A). Similarly, chromatin-bound SMARCA5 levels were not affected upon knockdown of Hdacs1,2 (Figure 6B). To further demonstrate SMARCA5 association with replicating regions, we used a modified ChIP assay to directly detect BrdU-labeled nascent DNA following immunoprecipitation. Cells were pulse-labeled with BrdU prior to formaldehyde crosslinking. Following chromatin immunoprecipitation with anti-SMARCA5 antibody, nascent DNA was detected using an anti-BrdU antibody in slot blot analysis. To confirm the validity of this modified ChIP assay, we performed BrdU-pulse chase analysis to look at the kinetics of PCNA loading onto nascent DNA in HeLa cells. In agreement with previously published results [13], PCNA loading to nascent DNA occurred rapidly within 15 min and disappeared after a 30-min chase (Figure 6C). In mouse NIH3T3 cells, we failed to obtain significant enrichment for the chromatin-bound PCNA relative to the background signal using the same antibody (data not shown). We examined the kinetics of SMARCA5 loading in these pulse-chase experiments in both HeLa and NIH3T3 cells. Our results showed that BrdU-labeled nascent DNA was present in the immunoprecipitate obtained using anti-SMARCA5 antibody at all chase time-points in both NIH3T3 and HeLa cells (Figure 6D-E). Additionally, inhibition or loss of Hdacs1,2 did not affect SMARCA5 association with nascent DNA (Figure 6F-G). It is conceivable that these bulk chromatin analyses might not allow detection of local changes at the replication forks. Therefore, we tested SMARCA5 occupancy at candidate replicating loci using ChIP assays. We found an increase in SMARCA5 occupancy at candidate early, mid- and late-replicating loci in S-phase cells when compared to its occupancy at these loci in the G0/G1 phase (Figure 7). We did not observe any significant change in SMARCA5 occupancy at these loci following treatment with Hdacs1,2-selective inhibitor (Figure 7). Taken together, these results demonstrate that SMARCA5 associates with sites of replication and its binding to nascent DNA and/or S-phase chromatin is not dependent on Hdacs1,2 activities.

### Loss of histone deacetylase 1 and 2 activities increases H4K16ac on nascent DNA and alters nascent chromatin structure

**Figure 8:**
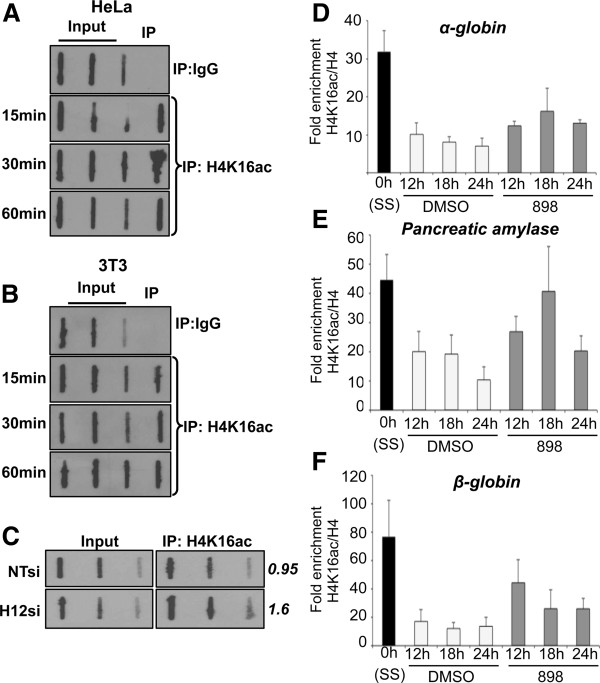
**Loss of histone deacetylase 1 and 2 (Hdacs1,2) increases H4K16ac on nascent DNA and at replication origins. A-B**. Bromodeoxyuridine (BrdU) pulse chase was performed to determine association of H4K16ac with nascent DNA at the indicated time points. **C**. BrdU-labeled nascent DNA associated with H4K16ac was measured by slot blot using input or immunoprecipitated DNA from cells transfected with non-targeting siRNA (NTsi) or Hdacs1,2-specific siRNA (H12si). BrdU-labeled nascent DNA was detected using an anti-BrdU antibody. Average BrdU signal of high and medium volume of chromatin immunoprecipitation (ChIP) DNA spotted on the slot was quantified by the Image J software and is shown on the right hand side of the panel. **D-F**. Changes in H4K16ac levels at candidate origins were measured by ChIP assays using NIH3T3 cells either serum-starved (SS, 0 h) or released into S-phase for the indicated time periods in the presence of either DMSO or 3.75 μM 898. Fold enrichment for H4K16ac was determined after normalization of H4K16ac occupancy to the occupancy of H4 at a given locus from three independent treatments.

ISWI-family chromatin remodelers have a C-terminal SANT domain that binds histones and the histone H4 N-terminus is required to stimulate ISWI ATPase activity [44]. H4K16ac inhibits the ISWI-family chromatin remodeling ATPases [15]. Therefore, H4K16ac can inhibit chromatin remodeling in addition to its role in preventing chromatin compaction. Since H4K16ac is increased upon loss or inhibition of Hdacs1,2 (Figure 5C-E), we hypothesized that increased H4K16ac at replication forks might alter nascent chromatin structure by affecting SMARCA5 activity and/or by affecting nascent chromatin compaction/packaging. Given our bulk chromatin analysis (Figure 5C-E), we first tested whether H4K16ac was present on nascent DNA. Indeed, using the modified ChIP assay, we found H4K16ac associates with BrdU-labeled nascent DNA in both HeLa and NIH3T3 cells (Figure 8A-B). Additionally, inhibition or loss of Hdacs1,2 increased H4K16ac levels associated with newly synthesized DNA (Figure 8C, Additional file 20: Figure S19). Standard ChIP analysis showed the presence of H4K16ac at candidate replication origins (Figure 8D-F). Interestingly, H4K16ac levels at these loci are higher in G0/G1 phase and are reduced once cells enter and progress through the S-phase, likely due to deacetylation by HDACs. Indeed, H4K16ac levels are increased at these origins following treatment with Hdacs1,2-inhibitor in S-phase cells (Figure 8D-F). Taken together, these results show that H4K16ac occurs at sites of replication and emphasizes a direct role for Hdacs1,2 in the deacetylation of this mark during replication in mammalian cells. Furthermore, it puts forth the possibility that increased H4K16ac at replication forks might inhibit SMARCA5 activity and/or chromatin packaging, which in turn might adversely affect nascent chromatin structure and replication fork rates.

**Figure 9:**
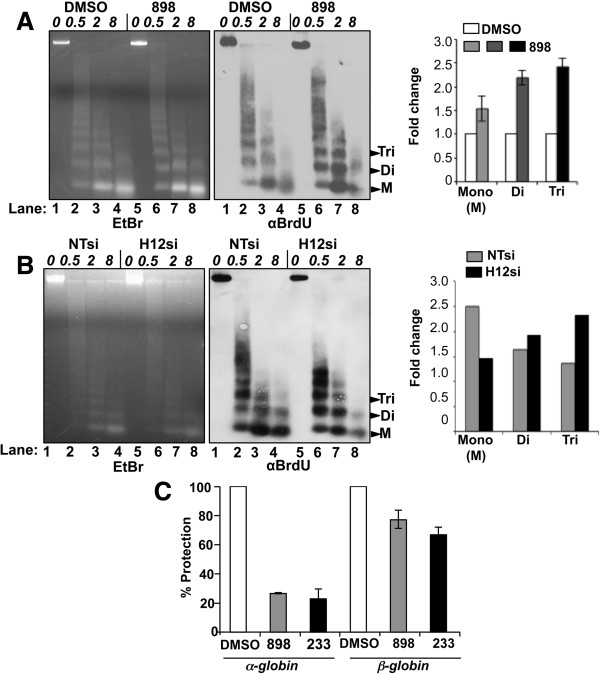
**Loss of histone deacetylase 1 and 2 (Hdacs1,2) affects nascent chromatin structure. A-B**. Nuclei were isolated from cells treated with either DMSO or 898 **(A)**, or from cells transfected with non-targeting (NT) siRNA or Hdacs1,2-siRNA **(B)**, bromodeoxyuridine (BrdU)-labeled nuclei were digested performed with various micrococcal nuclease (MNase) concentrations (0, 0.5, 2 and 8 units) for 5 min at 37°C. Equal amount of purified DNA was subjected to agarose gel electrophoresis. Pixel intensity of mono, di- and trinucleosomes obtained from 0.5 units MNase for control and knockdown samples and 2 units MNase for DMSO or inhibitor-treated samples were measured using the ImageJ software. Quantitative data for nucleosome levels and input levels were obtained using densitometry from four independent experiments for inhibitor treated samples and two independent experiments for knockdown samples. Nucleosome intensities were normalized to input intensities. The average intensity for control was set as 1 and fold-increase in nucleosome intensities in treated samples were determined in comparison to the control. Error bars denote standard error of the mean from multiple experiments. NTsi and H12si refer to non-targeting and Hdacs1,2-siRNA, respectively. **C**. Mononucleosome sensitivity was determined in S-phase synchronized cells as described in the Methods section. Fold-change in MNase sensitivity for a given region in S-phase cells was determined by qRT-PCR and normalized to the sensitivity of the locus in serum-starved samples. The plotted data represents an average +/− standard error of 3 independent treatments and the experiment was repeated two times. **D**, 8 and 2 refer to DMSO, 898 and 233 treatments, respectively. EtBr, ethidium bromide.

Given the increase in H4K16ac on nascent chromatin, we sought to determine whether Hdacs1,2 play a role in maintaining chromatin structure during replication. Therefore, we tested the sensitivity of nascent DNA to micrococcal nuclease (MNase) digestion in the absence of Hdacs1,2 functions. Cells were treated with either DMSO (control) or 898 to inhibit Hdacs1,2 (Figure 9A), or transfected with non-targeting siRNA (control) or Hdacs1,2-specific siRNA to knockdown the two enzymes (Figure 9B). Newly synthesized DNA was pulse-labeled with BrdU prior to nuclei isolation and MNase digestion. BrdU-labeled nascent DNA was detected using an anti-BrdU antibody. No defects in the global chromatin structure were observed following inhibition or loss of Hdacs1,2 functions, as evidenced by the ethidium bromide staining of MNase-digested DNA (Figure 9A-B, left panels). For BrdU-labeled nascent DNA, we observed a consistent increase in dinucleosomes and trinucleosomes released at lower MNase concentrations following treatment with 898 compared to DMSO alone (Figure 9A, compare lanes 6 and 7 to 2 and 3). Similar results were obtained following knockdown of Hdacs1,2 when compared to the control (Figure 9B, compare lanes 6 and 7 to 2 and 3). At high MNase concentration, nascent DNA associated with mononucleosomes appears to be very sensitive to nuclease digestion in the absence of Hdacs1,2 functions compared to the control (Figure 9A-B, compare lanes 4 and 8). This apparent increased sensitivity of nascent mononucleosomal DNA might be due to reduced BrdU incorporation in the absence of Hdacs1,2 functions (Figure 2B). Alternatively, the structure of nascent DNA associated mononucleosomes might indeed be altered in the absence of Hdacs1,2 activities due to increased histone acetylation, and thus, rendering them more sensitive to MNase digestion. To test this possibility, we digested nuclei isolated from S-phase cells treated with DMSO or 898 extensively with MNase and purified the DNA associated with mononucleosomes (approximately 146 bp). This purified DNA was then used as the template in quantitative PCR to assess sensitivity of nucleosomes at candidate replicating loci to nuclease digestion. We found nucleosomes at *α-globin* and *β-globin* loci to be sensitive to nuclease digestion following treatment with 898 to inhibit Hdacs1,2 activities (Figure 9C). We were unable to amplify across the pancreatic amylase locus following MNase digestion, probably because this region might be nucleosome-deficient or contains labile nucleosomes that are hypersensitive to nuclease digestion. Collectively, our MNase digestion assays show that Hdacs1,2 activities are required to maintain normal structure of nascent chromatin during DNA replication.

### Loss of ISWI-family chromatin remodeler SMARCA5 inhibits replication fork velocity

**Figure 10:**
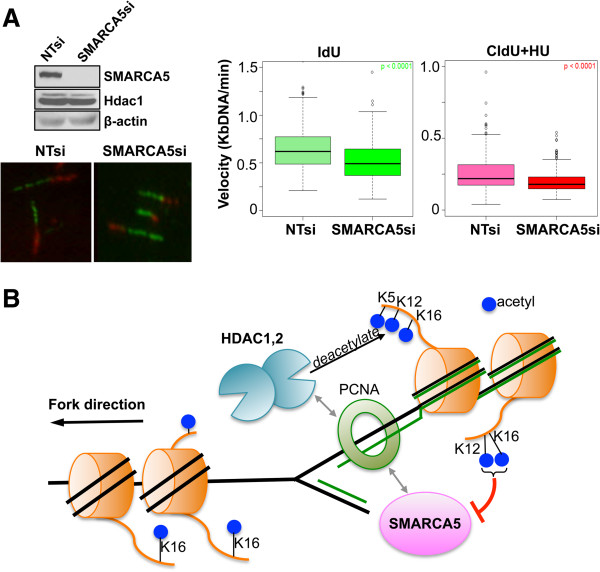
**Knockdown of SMARCA5 decreases replication fork velocity. A**. Western analysis of whole cell extract prepared from HeLa cells transfected with either non-targeting (NT) siRNA or SMARCA5 siRNA at 72 h time point following transfection. Histone deacetylase 1 (Hdac1) and β-actin serve as loading controls. Replication fork velocity was measured in HeLa cells transfected with either non-targeting (NT) or SMARCA5 siRNA at 72 hr time point following transfection. Box plots from were derived from data obtained in three independent experiments. Representative DNA fibers are shown in the left panel. **B**. Model for the functions of Hdacs1,2 during DNA replication. Hdacs1,2 and SMARCA5 interact with proliferating cell nuclear antigen (PCNA) and recruited to forks during replication. Hdacs1,2 deacetylate H4K5ac, H4K12ac and H4K16ac. H4K12ac and H4K16ac inhibit SMARCA5 nucleosome remodeling activity. Deacetylation restores chromatin structure allowing proper inter-nucleosomal interactions and remodeler-driven nucleosome positions to support the progression of the replication fork. Green line, newly replicated DNA. Orange disk, nucleosome with histone tail(s). Blue circle, acetyl group.

We next sought to determine whether SMARCA5 plays a role in the proper progression of DNA replication in mammalian cells. To this end, we measured replication fork velocity following knockdown of SMARCA5. We could achieve efficient knockdown of SMARCA5 in HeLa cells (Figure 10A), and no significant cell death was observed at 72 h post-transfection. As described for Hdacs1,2 (Figure 2C-D), we performed molecular combing analysis to determine changes in the replication fork rate following knockdown of SMARCA5 in the absence or presence of hydroxyurea. We found a consistent reduction in the replication fork velocity in the absence of SMARCA5 (Figure 10A, IdU label, Additional file 21: Figure S20, see high velocity 0.63 to 1.6 Kb/min rates), which was further exaggerated in the presence of hydroxyurea (Figure 10A, CldU label, Additional file 21: Figure S20, see 0.32 to 1.6 Kb/min rates). Taken together, our results show that SMARCA5 is required for maintaining normal replication fork rates similar to Hdacs1,2. We further examined if DNA damage response is activated in the absence of SMARCA5. Loss of SMARCA5 alone caused a very modest increase in the percentage of cells with γH2AX foci in HeLa cells (approximately 10%) (Additional file 22: Figure S21). However, loss of SMARCA5 combined with hydroxyurea treatment led to an increase in the percentage of cells with bright γH2AX staining (Additional file 22: Figure S21). These results together suggest that SMARCA5 is required for the proper progression of DNA replication and maintaining genome stability during S-phase similar to Hdacs1,2.

## Discussion

### Histone deacetylase 1 and 2 control nascent chromatin structure

Modulation of chromatin structure around a replication fork is achieved by the concerted action of histone variants, histone modifying enzymes, chromatin remodelers, histone chaperones and numerous chromatin-binding factors. It is conceivable that histone acetylation might be required to maintain a permissive chromatin conformation for the replication fork to progress. During S-phase, newly synthesized histone H4 is acetylated at K5 and K12 residues and deposited onto nascent chromatin by CAF-1, a histone chaperone [18]. Also, H4K16ac is enriched at initiation zones and at early replication regions [45]. Removal of H4K5ac and H4K12ac by HDACs following their deposition onto nascent chromatin was proposed to be an event in chromatin maturation during DNA replication [18]. Using selective inhibitors, we now show that Hdacs1,2 target histone deposition marks within S-phase cells and on nascent DNA (Figure 5A). Since H4K16ac prevents chromatin compaction, we propose a model, wherein Hdacs1,2 remove H4K16ac to allow chromatin restoration/reassembly step following DNA replication (Figure 10B). Indeed, we find that in the absence of Hdacs1,2 activities the nascent chromatin and nucleosomes at candidate replication loci are more susceptible to nuclease digestion (Figure 9), indicative of a more ‘permissive’ or ‘loose’ chromatin conformation. Hence, it is tempting to speculate that this atypical chromatin structure signals the replication machinery to stall, collapse, and trigger the DNA damage response. The resulting double-strand break and S-phase lesions could cause severe chromosome segregation defects. We find DNA damage and stress response activation upon inhibiting Hdacs1,2 (Figures 3A-B, Additional files 12, 13, 14: Figures S12, S13, S14), which corroborates previous findings that loss of Hdacs1,2 causes severe mitotic catastrophe [8].

### Role for histone deacetylase 1 and 2 in DNA replication via regulation of SMARCA5 function

Chromatin maturation not only involves histone deacetylation, but also nucleosome remodeling. Human cells contain two isoforms of ISWI: SNF2H and SNF2L [46]. The SNF2H/SMARCA5 complex remodels nucleosomes to allow smooth movement of the fork, especially through the heterochromatin [47]. SMARCA5 interacts with PCNA and with Hdacs1,2 [43,48]. In yeast, loss of Iswi2 and Ino80 causes defects in replication fork progression [49]. Mammalian ACF1-SNF2H is required for replication through heterochromatin, and depletion of SMARCA5 decreases BrdU incorporation in HeLa cells [47]. However, function for SMARCA5 in fork elongation in mammalian cells was not known. In this study, we show that SMARCA5 associates with nascent DNA (Figure 6D), and its occupancy on chromatin and at candidate replication origins increases in S-phase (Figure 6A and Figure 7). Importantly, we show that SMARCA5 has a direct role in controlling fork elongation, as its deletion reduces replication fork rates (Figure 10A).

The ISWI family chromatin remodelers have the catalytic ATPase domain and a SANT domain that binds the H4 tail. H4K16ac and H4K12ac were shown to interfere with the ability of ISWI to interact with the H4 tail and they inhibit the ATPase activity of the ISWI complex [15,16]. We show Hdacs1,2 target H4K16ac on newly synthesized DNA (Additional file 20: Figure S19). Also, we show that H4K12ac and H4K16ac are targeted by Hdacs1,2 in S-phase cells (Figure 5A, Figure 8D-F). Interestingly, we find an inverse correlation for the occupancy of H4K16ac and SMARCA5 at candidate replication origins (*α*-*globin*, *β*-*globin* and *pancreatic amylase*) in S-phase cells (Figure 7, Figure 8D-F). While H4K16ac levels are high at these loci in the non-replicating G0/G1 phase, they are reduced by Hdacs1,2 as cells enter and progress through the S-phase (Figure 8D-F). On the other hand, SMARCA5 levels are low at these loci in G0/G1 phase and increase during the S-phase. Therefore, these findings support a model (Figure 10B), wherein Hdacs1,2 might regulate SMARCA5 activity at the replication forks via removal of H4K12ac and H4K16ac. Increase in H4K12ac and H4K16ac around the fork upon loss or inhibition of Hdacs1,2 functions might inhibit SMARCA5-mediated chromatin remodeling, which is necessary for fork progression (Figure 10A), resulting in fork stalling and collapse. Collectively, we favor a model (Figure 10B), wherein Hdacs1,2 control nascent chromatin structure in two modes: one, affecting nucleosome structure and chromatin packaging by directly regulating histone acetylation and two, by regulating nucleosome remodeling via modulation of chromatin remodeler activity. Since abrogation of Hdacs1,2 functions alone is sufficient to impair DNA replication and compromise chromatin and genome stability during S-phase, selective inhibition of Hdacs1,2 might be an efficient therapeutic strategy to minimize the side effects of pan-HDIs that are currently used in cancer treatment.

## Conclusions

In this study, we report functions for Hdacs1,2 in maintaining normal replication fork progression and in nascent chromatin maintenance using novel Hdacs1,2-selective inhibitors and siRNA-mediated genetic knockdown systems. SAHA, a pan-inhibitor that targets all class I HDACs, was shown to affect replication fork velocity in cancer cells without affecting transcription [50]. Here, we show that Hdacs1,2 (a subset of SAHA targets) play a direct role in DNA replication without disrupting transcription of genes involved in DNA replication, repair or nucleotide biosynthesis. We further show that inhibiting Hdacs1,2 alters nascent chromatin architecture (histone acetylation and compaction), reduces replication fork velocity and triggers DNA damage response. These findings highlight the important role for Hdacs1,2 in genome stability maintenance. In addition, we show that SMARCA5, an ISWI-family chromatin remodeler, is present on nascent chromatin and is required for proper progression of DNA replication. Therefore, in this study, we have connected the functions of a chromatin remodeler (SMARCA5), histone modifications (H4K12ac and H4K16ac) and histone deacetylases (Hdacs1,2 that target H4K12ac and H4K16ac) to the progression of replication fork.

## Methods

### Cell culture

HeLa and HEK 293 cells were cultured in DMEM containing 10% fetal bovine serum (Hyclone, Logan, UT, USA), 1% penicillin-streptomycin and 1% glutamine. NIH3T3 cells were serum starved for 72 h in 0.5% serum containing media. NIH3T3 cells were cultured in DMEM (Cellgro™, Tewksbury, MA, USA) containing 10% fetal calf serum, 1% penicillin-streptomycin and 1% glutamine. NIH3T3 cells were serum starved for 72 h in 0.5% serum containing media. Fibrosarcoma cells for conditional knockout of Hdac1,2 or Hdac3 were cultured as described previously [8].

### siRNA knockdown

Cells were transfected with siGenome SMART pool for mouse Hdac1, or siGenome SMART pool for mouse Hdac2, siGenome SMART pool for human SMARCA5, or with non-specific control pool (siRNA negative control) as described previously [14]. All siRNAs were purchased from Dharmacon (Lafayette, CO, USA).

### Replication fork velocity measurement

NIH3T3 cells transfected with non-targeting (NT) or Hdac1 & Hdac2 siRNAs were labeled with 20 μM IdU (iodo-deoxyuridine) for 15 min following 72 h post-transfection. Cells were washed with PBS and labeled with 100 μM CldU (chloro-deoxyuridine) in the presence of 250 μM hydroxyurea for 20 min. Cells were lysed with spreading buffer (0.5% SDS in 200 mM Tris–HCl, pH 7.4 and 50 mM EDTA) and DNA fibers were spread on silane-coated slides. Following fixation and DNA denaturation, immunofluorescence (IF) was performed using anti-IdU and anti-CldU antibodies and mouse anti-BrdU-conjugated to Alexa 488 and rat anti-CldU conjugated to Alexa 594 (secondary antibodies). Fiber images were captured using an Axioscope microscope. The lengths of approximately 100 fiber tracks were measured using the ImageJ software. The fiber length (μm) was converted into Kb DNA length after taking the stretching factor (1 μm = 2 Kb DNA) into consideration. The resulting value was then divided by the incubation time to obtain the fork velocity.

### Chromatin immunoprecipitation assay

ChIP assays were performed as described previously [51].

### Immunofluorescence analysis

Immunofluorescence was performed as described previously [14].

### Bromodeoxyuridine-propidium fluorescence-activated cell sorting analysis

BrdU-PI analysis was performed as described previously [14].

### PCR primers

PCR primers for ChIP analysis at replication origins were described previously [17].

### Nuclei isolation for micrococcal nuclease digestion

NIH3T3 cells were labeled with 20 μM BrdU, washed with ice-cold phosphate-buffered saline (PBS). Cell lysis buffer (10 mM Tris–HCl, pH 7.4, 300 mM sucrose, 3 mM CaCl_2_, 2 mM Mg(CH3COO)2, 0.5% NP-40, 5 mM dithiothreitol (DTT) and 1X Roche protease inhibitor cocktail) was added to the plate and left on ice for 5 min. Cells were scraped following lysis, Dounce homogenized fifty times, spun at 1000 rpm for 5 min at 4°C. Nuclei were stored in nuclei storage buffer (50 mM Tris- HCl, pH 8.3, 40% glycerol, 0.1 mM EDTA, 5 mM Mg(CH3COO)2, 5 mM DTT and 1X Roche protease inhibitor cocktail) and stored at −80°C until use.

### Immunoprecipitation

Nuclear extract was prepared in RIPA buffer supplemented with protease inhibitors (Roche) and precleared with Protein A-agarose beads (Millipore, Billerica, MA, USA) for 20 min at 4°C with constant rotation. The precleared lysate was then incubated with anti-PCNA antibody for 12 h at 4°C with constant rotation. Protein A-agarose beads equilibrated in RIPA buffer was then added to the samples, and pull-down was done at 4°C for 1 h with constant rotation. The beads were then washed with RIPA buffer for three times and resuspended in 1X SDS sample buffer before Western analysis.

### Nuclear and chromatin extract preparation for immunoprecipitation and western blot analyses

NIH3T3 cells were washed with ice-cold PBS. Cells were scraped and spun at 3000 rpm at 4°C for 5 min. The cell pellet was resuspended in buffer A (10 mM HEPES, pH 7.9, 10 mM KCl, 1.5 mM MgCl2, 0.34 M sucrose, 10% glycerol, 1 mM dithiothreitol (DTT), and protease inhibitor cocktail). Triton X-100 (0.1% final concentration) was added to the extract, and incubated on ice for 8 min. Nuclei (fraction P1) were collected by centrifugation (5 min, 8000 rpm, 4°C). The nuclear pellet was resuspended in RIPA buffer containing protease inhibitor cocktail and sonicated to solubilize the chromatin. To prepare chromatin extract, the isolated nuclei (fraction P1) were resuspended in hypotonic buffer (3 mM EDTA, 0.2 mM EGTA, 1 mM DTT) supplemented with protease inhibitor cocktail (Roche, Penzberg, Upper Bavaria, Germany) and incubated on ice for 30 min. Following centrifugation, the pellet was resuspended in RIPA buffer containing protease inhibitors and sonicated briefly to solubilize the chromatin. Protein concentrations were measured using Bio-Rad Protein Assay or Bio-Rad DC™ protein assay kits (Bio-Rad, Hercules, CA, USA)’.

### Histone deacetylase assay

NIH3T3 cells were treated with DMSO (control) or the selective HDAC inhibitors for 24 h. Nuclei were isolated as described above and resuspended in HERR buffer (20 mM HEPES pH 7.9, 150 mM KCl, 0.1% NP40, 10% glycerol, 2 mM EDTA). Extracts were sonicated and precleared with Protein A-agarose beads (Millipore) for 20 min at 4°C with constant rotation. The precleared lysate was then incubated with anti-Hdac1 or -Hdac2 or -Hdac3 antibodies for 4 hr at 4°C with constant rotation. Protein A-agarose beads were then added to samples and pull down was done at 4°C for 1 hr with constant rotation. The beads were then washed with HERR buffer for three times and HDAC assay was performed as per the recommended protocol provided with the Fluor-de-Lys™ HDAC fluorometric activity assay kit (Enzo Life Sciences, Farmingdale, NY, USA). The activity was measured using the 2104 EnVision™ Multilabel Reader (PerkinElmer; excitation at 340 nm and emission at 495 and 520 nm). *In vitro* HDAC assays were performed using recombinant HDAC enzymes and the Fluor-de-Lys™ HDAC fluorometric activity assay kit.

### Micrococcal nuclease sensitivity analysis of nascent DNA

Nuclei were isolated from cells treated with DMSO (control) or Hdacs1,2-selective inhibitor (898), or from cells transfected with non-targeting siRNA or Hdacs1,2-specific siRNA followed by labeling with 20 μM BrdU for 1 hr. MNase digestion of nuclei was performed essentially as described previously [51]. Briefly, equal numbers of nuclei were digested with 0, 0.5, 2 or 8 units of MNase (Worthington Biochemical Co., Lakewood, NJ, USA) for 5 min at 37°C. Nuclease digestion was terminated following addition of equal volume of 2X stop buffer (20 mM Tris. Cl, pH 7.5, 200 mM NaCl, 2% SDS and 20 mM EDTA). Following nuclease digestion, genomic DNA was isolated using the Easy-DNA™ Kit (Invitrogen) as per the manufacturer’s instructions. DNA concentration was measured using NanoDrop 1000 spectrophotometer (ThermoScientific). Undigested DNA (0.5 μg) or MNase-digested DNA (1 μg) were resolved in a 1.5% agarose gel and stained with ethidium bromide. The DNA was then transferred onto a charged membrane (Hybond™-N+, GE Healthcare, Pittsburgh, PA, USA) using capillary transfer following the standard Southern blotting procedure. The membrane was subjected to UV crosslinking to immobilize DNA. Membrane-bound BrdU-labeled nascent DNA was detected by Western blotting using an anti-BrdU antibody (used at 1:2000 dilution).

### Locus-specific micrococcal nuclease sensitivity assay

NIH3T3 cells were serum starved for 72 h and released into serum-rich medium to progress into S-phase in the presence of DMSO or 3 μM Hdacs1,2-selective inhibitor (898) and treated for 20 h prior to crosslinking with 0.1% formaldehyde for 10 min. Serum-starved NIH3T3 cells prior to release into S-phase were also crosslinked similarly with formaldehyde. Crosslinking was quenched by adding glycine (125 mM final concentration) and incubating for 5 min at room temperature. Nuclei were isolated as described above and counted. Equal number of nuclei from different treatments (0.5 × 106) were washed once in Buffer D (50 mM Tris- HCl, pH 8.0, 25% glycerol, 0.1 mM EDTA, 5 mM Mg(CH3COO)2, 5 mM DTT) and then resuspended in 0.2 ml of Buffer MN (15 mM Tris, pH 7.4, 60 mM KCl, 15 mM NaCl, 0.5 mM DTT, 0.25 M sucrose and 1 mM CaCl2). Nuclei (0.1 ml) were digested with micrococcal nuclease (10 units, Worthington Biochemical Co.) by incubation for 7 min at 37°C and 400 μg RNase A (Qiagen, Vinlo, Limberg, Germany) was added and digestion was continued for an additional 3 min. Digestion was stopped by adding an equal volume of 2X stop buffer (20 mM Tris. Cl, pH 7.5, 200 mM NaCl, 2% SDS and 20 mM EDTA). To the remaining nuclei (0.1 ml, set aside for undigested DNA control) an equal volume of 2X stop buffer was added. DNA from undigested and MNase-digested nuclei was isolated using Qiagen DNeasy™ Kit. MNase-digested DNA was resolved in a 1.8% agarose gel. Mononucleosomal DNA (approximately 146 bp) was excised and extracted from the gel using Qiagen Gel Extraction Kit. The yield of undigested DNA and purified mononucleosomal DNA was measured using Quant-iT™ PicoGreen™ dsDNA Assay Kit (Invitrogen, Carlsbad, CA, USA) in a Qubit™ 2.0 Fluorometer. DNA amounts obtained from various samples were normalized to make them equal. Equal amount of DNA was then used as template in qPCR in real time using the primers for *α-globin*, *β-globin* and *pancreatic amylase* loci. For each primer, percentage protection was calculated by comparing Ct values of undigested DNA versus mononucleosomal DNA for DMSO or 898 treatments and further normalized to Ct values of undigested DNA versus mononucleosomal DNA obtained from the serum starved cells.

### Slot blot analysis of bromodeoxyuridine-labeled DNA

For measuring BrdU incorporation, NIH3T3 cells in S-phase were treated with DMSO or 3 μM Hdacs1,2-selective inhibitor (898) for 12 h, 18 h or 24 h followed by labeling of cells with 20 μM BrdU for 1 hr. Genomic DNA was isolated using Qiagen DNeasy™ Kit and extensively digested with RNaseA. DNA was quantified in a Qubit™ 2.0 Fluorometer using Quant-iT™ PicoGreen™ dsDNA Assay Kit (Invitrogen). Equal amount DNA (500 ng) from the different samples was resuspended in 40 μl water and denatured by adding 10 volumes of 0.4 N NaOH and incubation at room temperature for 30 min. Equal volume of 1 M Tris. Cl, pH 6.8 was added to neutralize and samples were placed on ice. Aliquots were made to obtain varying amounts of DNA (50, 25 and 12.5 ng) for each sample in a total volume of 100 μl. DNA was then transferred on to a Zeta-Probe GT Membrane (Bio-Rad, Hercules, CA, USA) using a Slot Blot apparatus (Schleicher and Schuell Minifold II). DNA was immobilized to the membrane using a UV crosslinker and western blotting was done with anti-BrdU antibody. The linear range for detecting BrdU-labeled DNA was initially determined using DNA isolated from the DMSO control. Using a two-fold serial dilution of DNA, we determined that 50 ng to 6.25 ng to be in the linear range of detection in western blotting using the anti-BrdU antibody (1:500 dilution) and ECL2 Western Blotting Substrate (Thermo Scientific Pierce, Waltham, MA, USA).

For the analysis of BrdU-labeled DNA obtained from ChIP assays, input and ChIP DNA were eluted in 50 μl water. The input DNA yield was measured using NanoDrop 1000 spectrophotometer (ThermoScientific) to ensure that the DNA amount to be used in Slot Blot assay remains in the linear range of detection (<50 ng). The input and immunoprecipitated DNA (50 μl) were denatured by adding 2.5 volumes of 0.4 N NaOH and incubating at room temperature for 30 min. Equal volume of 1 M Tris–HCl, pH 6.8 (175 μl) was then added to neutralize and samples were placed on ice. A serial dilution of DNA from input and immunoprecipitated DNA for the Slot Blot assay were prepared as follows: For input DNA, 100 μl denatured DNA; 50 μl denatured DNA + 50 μl H2O; 25 μl denatured DNA + 75 μl H2O. For ChIP DNA, 200 μl denatured DNA; 100 μl denatured DNA + 100 μl H2O; 50 μl denatured DNA + 150 μl H2O. The DNA were then transferred on to a Zeta-Probe GT Membrane (Bio-Rad, Hercules, CA, USA) using a Slot Blot apparatus (Schleicher and Schuell Minifold II) and processed as described above. For the modified ChIP assays with Brdu pulse-chase, NIH3T3 or HeLa cells were labeled with 20 μM BrdU for 30 min. Following BrdU-labeling, cells were washed twice with PBS to remove unincorporated BrdU and grown in fresh medium. For the chase, cells were fixed with formaldehyde for ChIP analyses at 15 min, 30 min and 60 min time points.

### RNA-Seq analysis

Total RNA was isolated from NIH3T3 cells that were released into S-phase from serum starvation for 20 h either in the presence of DMSO or 3 μM 898 using the Versagene RNA isolation kit (5 Prime). RNA was prepared from three different sets of DMSO- and 898-treated cells and sequenced using the Illumina Hiseq2000 sequencer. Standard gene analysis was performed using the open source USeq/DESeq analysis packages. In brief, this involves aligning each replica dataset to a genome index that has the standard mm10 chromosomes plus an artificial chromosome containing all known and all theoretical splice junctions. After alignment splice junction coordinates are converted to genomic coordinates, counts for each gene was collected, and a multi-replica treatment versus control comparison was performed using DESeq (http://genomebiology.com/2010/11/10/R106). Genes passing two thresholds, an FDR of <10% and absolute log2 ratio of 1, were considered differentially expressed and used in subsequent analysis. A window scanning, no known annotation analysis was also performed using the USeq MultipleReplicaScanSeqs application. This analysis generated the genome wide log2 ratio and FDR window summary tracks.

### Protein analysis

For preparation of whole cell extracts, cell pellets were washed with PBS and sonicated in RIPA buffer with protease inhibitors (Roche protease inhibitor cocktail) prior to western analyses. Antibodies used in this study are listed in the supplementary table (Additional file 23: Table S2).

### Adenovirus-containing Cre recombinase infection

Ad-Cre infection of fibrosarcoma cells was performed as described previously [14].

## Abbreviations

ac: Acetylation; Ad-CRE: Adenovirus-containing Cre recombinase; AFU: Arbitrary fluorescence units; BrdU: Bromodeoxyuridine; CAF-1: Chromatin assembly factor-1; ChIP: Chromatin immunoprecipitation; CldU: Chlorodeoxyuridine; FACS: Fluorescence-activated cell sorting; HDAC: Histone deacetylase; Hdacs1,2: Histone deacetylase 1 and 2; HU: Hydroxyurea; HP1: Heterochromatin protein 1; IdU: Iododeoxyuridine; IP: Immunoprecipitated; MNase: Micrococcal nuclease; NT: Non-targeting; NTsi: Non-targeting siRNA; PCNA: Proliferating cell nuclear antigen; PCR: Polymerase chain reaction; RPA: Replication protein A; SAHA: Suberoylanilide hydroxamic acid; SMARCA5: SWI/SNF-related matrix associated actin-dependent regulator of chromatin subfamily A member 5; TBP: TATA binding protein; TSA: Trichostatin A.

## Competing interests

Vincent Jacques and James Rusche work for Repligen Corporation, which has developed the HDAC selective compounds used in this manuscript, and they have financial interests in this company. All other authors declare that they have no competing interests.

## Authors’ contributions

SB designed and performed majority of the experiments, analyzed the data and wrote the manuscript. VJ and JR provided SB with novel Hdac1,2 selective inhibitors. EO provided Hdac1,2 and Hdac3 knockout fibrosarcoma cells. BC provided intellectual input on SMARCA5 experiments and did a critical reading of the manuscript. MC performed chromatin structure experiments and provided critical comments on the manuscript. All authors read and approved the final manuscript.

## Supplementary Material

Additional file 1: Figure S1 Hdac1 and Hdac2 are present at candidate mid- and late-replication origins during S-phase. A-B. Serum-starved NIH3T3 cells were released into S-phase and cells were cross-linked at 12 h, 18 h and 24 h time points following release. Serum starved cells (0 h) were also cross-linked and used as a control. The levels of Hdac1 and Hdac2 at *pancreatic amylase* (mid-late replicating) and *β-globin* (late replicating) in synchronized NIH3T3 cells were determined using ChIP assays. Data represents average of three independent ChIP experiments. Error bars, standard error of the mean. Click here for file

Additional file 2: Figure S2 Loss of Hdac1 alone or Hdac2 alone has no effect on H4K5ac. Fibrosarcoma cells containing floxed alleles of *Hdac1* (*Hdac1*^*Fl*/*Fl*^) were infected with Ad-Cre (*Hdac1*^*Fl*/*Fl*^ + Cre) to deplete Hdac1. Cells without Ad-Cre infection (*Hdac1*^*Fl*/*Fl*^ –Cre) were used as wild type control. Also, cells without Ad-Cre infection were transfected with *Hdac2*-specific siRNA (*Hdac1*^*Fl*/*Fl*^ + Hdac2siRNA) to deplete only Hdac2. Western blot of chromatin fractions was performed to check depletion of either Hdac1 or Hdac2 and the levels of H4K5ac. H4 was used as the loading control. KO, knockout; KD, knockdown; WT, wild type. Click here for file

Additional file 3: Figure S3 Characterization of novel Hdac1,2-selective inhibitors. A. *In vitro* enzyme assays using recombinant HDAC proteins to determine the specificity of 233 and 898 towards Hdacs1 and 2. Numbers in the table represent IC50 values obtained for an inhibitor-enzyme combination in the *in vitro* HDAC assays. AFU refers to arbitrary fluorescent units. B. Dose range of RGFP898 that induces increase in histone acetylation was determined by western analysis of whole cell extracts prepared from NIH3T3 cells following treatment for 24 h with DMSO or increasing concentrations of 898 (μM). H3 and H4 levels serve as loading controls. Click here for file

Additional file 4: Figure S4 Western analysis of extracts used *in vivo* HDAC enzyme assays. Hdac1 or Hdac2 or Hdac3 were immunoprecipitated from nuclear extracts following treatment of NIH3T3 cells with either DMSO (D), 3 μM 898 (panel A) or 3 μM 233 (panel B). Immunoprecipitated HDACs were then used in enzyme assays to measure inhibition by 898 or 233 (data shown in Figure 1D-E). Western blots were performed using antibodies against each HDAC to confirm the presence of the enzyme and the efficiency of individual immunoprecipitation. Click here for file

Additional file 5: Figure S5 Effect of 898 and 233 on H4K5ac levels in cells following conditional knockout of Hdac1,2 or Hdac3. Quantitative data for western blots shown in Figure 1: H-I. A-B. Fibrosarcoma cells containing floxed alleles of *Hdac1* and *Hdac2* (*Hdac1*^*Fl*/*Fl*^*Hdac2*^*Fl*/*Fl*^) were infected with Ad-Cre for 48 h to deplete both Hdac1 and Hdac2, and then treated with either DMSO or 3 μM 233 (A) or 3 μM 898 (B) for 24 h. C-D. Fibrosarcoma cells containing floxed alleles of *Hdac3* (*Hdac3*^*Fl*/*Fl*^) were infected with Ad-Cre and treated with DMSO or 3 μM 233 (C) or 3 μM 898 (D) as described for panels A-B. Western analysis of H4K5ac and H4 was performed with whole cell lysates. For each treatment, signals for H4K5ac were normalized to the signals for H4 (loading control). Normalized signals for H4K5ac obtained from two independent experiments are shown. Click here for file

Additional file 6: Figure S6 Inhibition of Hdac1,2 activities for 24 h or knockdown of Hdac1,2 for 72 h does not affect the cell cycle. A and B. FACS analysis of Propidium iodide (PI) stained NIH3T3 cells treated with DMSO or 3 μM 898 (A) or 3 μM 233 (B) for 24 h. C. FACS analysis of Propidium iodide (PI) stained NIH3T3 cells transfected with either non-targeting (NT) or Hdac1,2 siRNA transfected cells at 72 h time point following transfection. Data shown is a representative of two independent experiments. Click here for file

Additional file 7: Figure S7 Inhibition of Hdac1,2 activities with 233 does not affect S-phase progression. Serum-starved NIH3T3 cells were released into S-phase in the presence of DMSO or 3 μM 233. FACS analysis following BrdU-propidium iodide labeling and staining of cells was performed at 0 h, 12 h, 18 h and 24 h time points following release into S-phase. Click here for file

Additional file 8: Figure S8 Prolonged treatment of NIH3T3 cells with 898 causes a G1 arrest. A. FACS analysis following propidium iodide staining of NIH3T3 cells was performed at 24 h and 48 h time points following treatment with DMSO or 3 μM 898. B. FACS analysis of BrdU-propidium iodide stained cells following 24 h or 48 h treatment with 3 μM 898. Percentage of BrdU-positive S-phase cells is indicated. Click here for file

Additional file 9: Figure S9 BrdU incorporation is reduced following treatment with 233. Slot blot analysis of BrdU-labeled genomic DNA following treatment with 233 was performed using the indicated amounts of purified DNA. For quantitation, BrdU incorporation in various samples was measured using densitometry. Average BrdU incorporation from three independent experiments are shown. Click here for file

Additional file 10: Figure S10 Replication fork velocity is affected following treatment with Hdac1,2-selective inhibitors or knockdown of Hdacs1,2. DNA combing to measure replication fork velocity in NIH3T3 cells was performed 20 h following release into S-phase from serum starvation in the presence of DMSO or 3 μM 898 (A) or following siRNA knockdown of Hdacs1,2 (B). Cells were labeled with IdU (green) for 15 min and then with CldU (red) in the presence of 250 μM hydroxyurea for 20 min. DNA fibers were prepared and analyzed as described in the Methods section. Data represent average fork velocity of DNA fibers prepared from four independent experiments with 898 treatment or 3 independent experiments with 233. Data from one knockdown experiment is shown in the figure and the analysis was repeated twice. In each experiment, at least 100 fibers in different areas of the slide from three different slide preparations were analyzed. Error bars, standard error of the mean from independent experiments. Box plot representation of these data is shown in Figure 2C and 2D. Click here for file

Additional file 11: Figure S11 Replication fork velocity is reduced upon treatment with SAHA. A. NIH3T3 cells were treated with either DMSO or 10 μM SAHA for 24 h. Fibers were prepared and analyzed as described earlier in the methods section. Box-plot analysis was done with data obtained from two independent experiments. B. Percentage fibers with varying velocities are shown in panel B. Graph shows data from a representative experiment. In each experiment, at least 100 fibers in different areas of the slide from three different slide preparations were analyzed. Click here for file

Additional file 12: Figure S12 Quantitation of γH2AX foci in NIH3T3 cells following treatment with 3 μM 898 or 3 μM 233 for 48 hr. Cells with various amount of big and bright foci in a population of 100 cells were counted. Average percent of cells for each category obtained from four independent experiments is shown. Representative immunofluorescence image of γH2AX foci in the nuclei of control and Hdac1,2 inhibitor-treated cells is shown in Figure 3A. Click here for file

Additional file 13: Figure S13 DNA damage response is activated following knockdown of Hdacs1,2. DNA damage in non-targeting (NT) siRNA or Hdacs1,2-siRNA transfected NIH3T3 cells was measured at 72 h time point by immunofluorescence analysis by staining for γH2AX. Hoechst staining was performed to visualize nuclei. Cells with >5 γH2AX foci in a population of 100 cells were counted. Average of two independent experiments is shown. Click here for file

Additional file 14: Figure S14 DNA damage response is activated in primary mouse embryo fibroblast cells following 898 or 233 treatment. Wild-type mouse embryo fibroblasts (MEF) isolated from 13.5 (p.c.) embryos were treated with 3 μM 898 or 3 μM 233 for 48 hr. Immunofluorescence of γH2AX was performed and percentage of cells with big bright foci were determined in two independent experiments. Majority of cells had 1–5 foci. Data represents average number of cells with γH2AX foci obtained from two different MEF preparations. Click here for file

Additional file 15: Figure S15 Quantitation of Rad51 foci formed in S-phase cells following inhibition of Hdacs1,2. Serum-starved NIH3T3 cells were released into S-phase and treated with either DMSO or 3 μM 233 or 3 μM 898 in the presence of 500 μM hydroxyurea. Immunofluorescence staining for Rad51 was performed and percentage of cells with greater than 5 foci were determined from two independent experiments, with each experiment containing duplicate treatments. Representative immunofluorescence images with Rad51 foci are shown in Figure 3B. Click here for file

Additional file 16: Figure S16. Inhibition of Hdac1,2 activities in S-phase cells does not alter expression of genes coding for replication-associated factors. Serum-starved NIH3T3s were released into S-phase in the presence of DMSO or 3 μM 898 for 20 h. Three independent DMSO and 898 treatments were performed for total RNA isolation. Purified RNA was subjected to RNA-seq analyses. A. Four genes that were consistently up-regulated in all three 898 treated samples compared to the DMSO control. B. Levels of DNA damage-associated genes (ATM, Rad21) and replication-associated genes (MCM2, CTPS, RPA1, RPA2, DNA polymerase γ (Polg) and SMARCAD1) remain unchanged in all the three 898-treated samples compared to DMSO control. Click here for file

Additional file 17: Table S1 List of genes that are differentially expressed in S-phase NIH3T3 cells following 3 μM 898 treatment. Click here for file

Additional file 18: Figure S17 Hdacs1,2 do not target Smc3 acetylation. A. Chromatin from serum-starved NIH3T3 cells that were released into S-phase in the presence of DMSO or 3 μM 898 for 0 h, 12 h, 18 h and 24 h was prepared for western analysis. Smc3 levels serve as the loading control. B. NIH3T3 cells were transfected with either non-targeting (NT) or Hdac1,2 (H12) siRNA prior to chromatin extract preparation, and levels of Smc3ac and Smc3 were examined using western analysis. Click here for file

Additional file 19: Figure S18 Inhibition of Hdac1,2 activities increases histone acetylation in S-phase cells. Quantitation of the western blot performed with pan-acetyllysine (pan-AcK) antibody shown in Figure 5B. Chromatin was prepared from NIH3T3 cells following release of cells into S-phase from serum starvation and western blot analysis with pan-AcK antibody was done to look at histone acetylation. Signals obtained for histones H3, H2A, H2B and H4 were measured by densitometry. Data represents cumulative signal for all four histones and the average signal for histone +/− standard error from three independent experiments is shown. Click here for file

Additional file 20: Figure S19 Loss of Hdacs1,2 increases H4K16ac on nascent chromatin. ChIP of H4K16ac was done and increasing volumes of ChIP DNA were spotted onto a membrane using Slot Blot and probed with anti-BrdU antibody. In A-B, a representative blot shown and the experiment with 3.75 μM Hdac1,2 inhibitor and siRNAs was repeated at least 3 times. Average BrdU signal of high and medium volume of ChIP DNA spotted on the slot was quantified by the Image J software and is shown on the right hand side of the figure. Click here for file

Additional file 21: Figure S20 Knockdown of SMARCA5 reduces replication fork velocity. Replication fork velocity was measured in HeLa cells transfected with either non-targeting siRNA (NTsi) or SMARCA5 siRNA at 72 h time point following transfection. Data represent average fork velocity calculated from three independent experiments. Error bars, standard error of the mean. Click here for file

Additional file 22: Figure S21 DNA damage response is activated in HeLa cells following knockdown of SMARCA5. A. HeLa cells were transfected with non-targeting (NT) or SMARCA5 siRNA and immunofluorescence was performed to measure γH2AX foci formation. A. Quantitation of percentage of cells with γH2AX foci in control (non-targeting siRNA transfected) or SMARCA5 siRNA transfected cells in the absence of hydroxyurea (HU) is shown in the left panel and cells with pan-nuclear γH2AX staining in control (non-targeting siRNA transfected) or SMARCA5 siRNA transfected cells in the presence of hydroxyurea (HU) is shown in the right panel. Average data from two independent siRNA transfections are shown. B. Representative images for pan-nuclear γH2AX staining in control (non-targeting siRNA-transfected) or SMARCA5 siRNA-transfected cells in the presence of hydroxyurea are shown. Click here for file

Additional file 23: Table S2 Details of the antibodies used in this study are listed in the supplementary table. Click here for file
