# Supplementary figures and images for: Histone deacetylases 1 and 2 maintain S-phase chromatin and DNA replication fork progression
Source: Epigenetics Chromatin. 2013 Aug 15;6:27. doi: 10.1186/1756-8935-6-27 (PMC3765969; doi:10.1186/1756-8935-6-27)

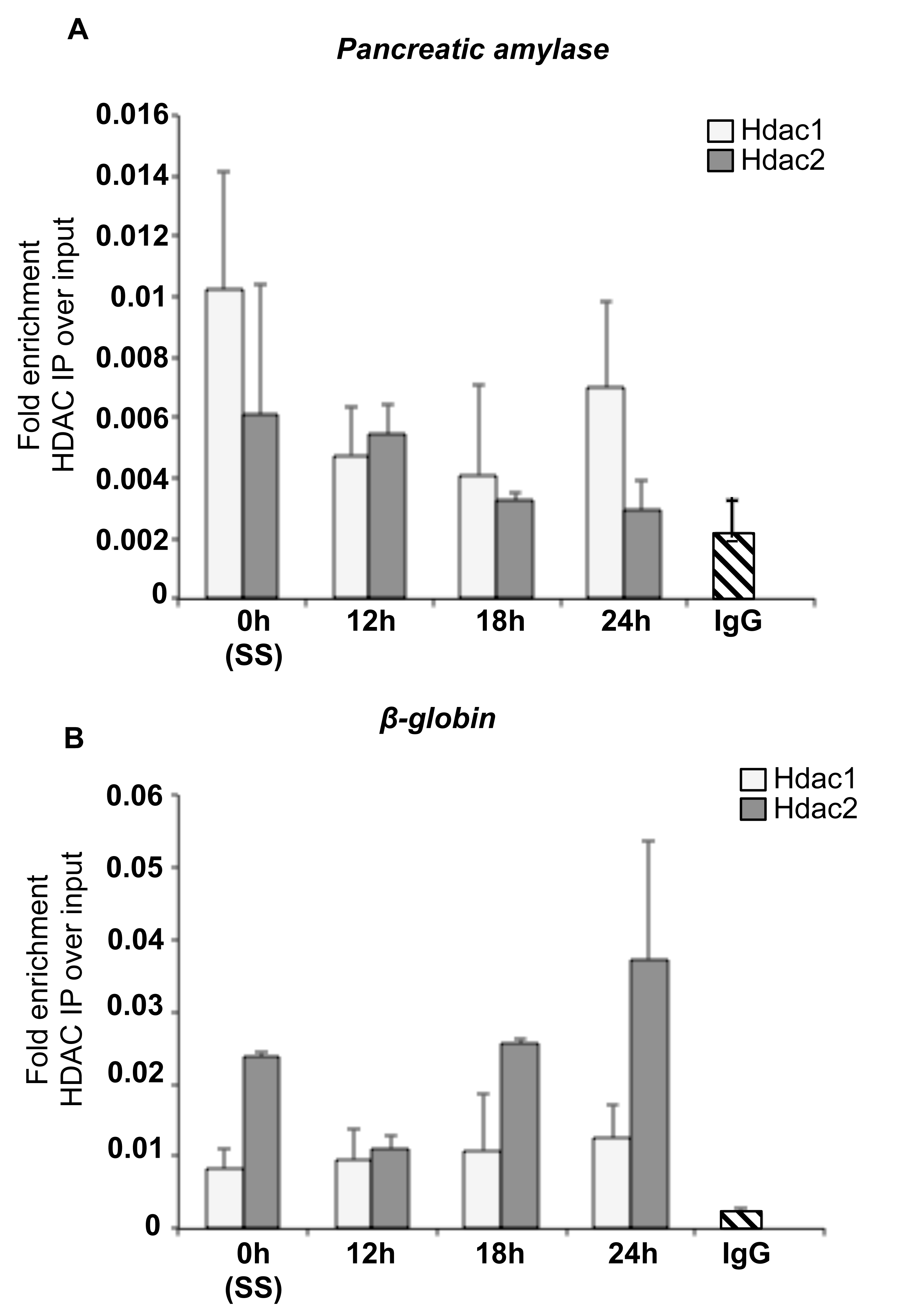

Supplement: Additional file 1: Figure S1 — Hdac1 and Hdac2 are present at candidate mid- and late-replication origins during S-phase. A-B. Serum-starved NIH3T3 cells were released into S-phase and cells were cross-linked at 12 h, 18 h and 24 h time points following release. Serum starved cells (0 h) were also cross-linked and used as a control. The levels of Hdac1 and Hdac2 at pancreatic amylase (mid-late replicating) and β-globin (late replicating) in synchronized NIH3T3 cells were determined using ChIP assays. Data represents average of three independent ChIP experiments. Error bars, standard error of the mean. [file 1756-8935-6-27-S1.tiff]

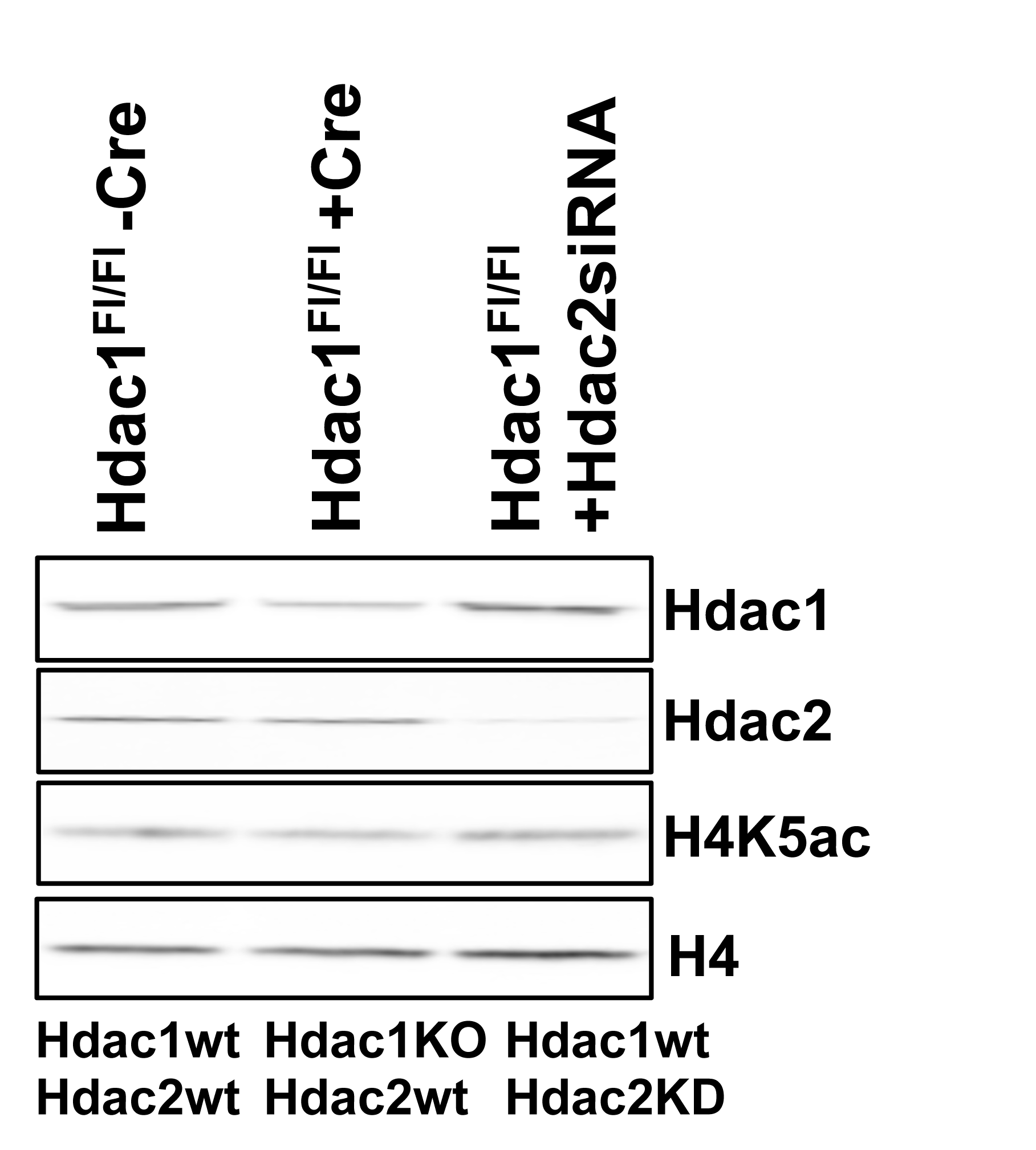

Supplement: Additional file 2: Figure S2 — Loss of Hdac1 alone or Hdac2 alone has no effect on H4K5ac. Fibrosarcoma cells containing floxed alleles of Hdac1 (Hdac1Fl/Fl) were infected with Ad-Cre (Hdac1Fl/Fl + Cre) to deplete Hdac1. Cells without Ad-Cre infection (Hdac1Fl/Fl –Cre) were used as wild type control. Also, cells without Ad-Cre infection were transfected with Hdac2-specific siRNA (Hdac1Fl/Fl + Hdac2siRNA) to deplete only Hdac2. Western blot of chromatin fractions was performed to check depletion of either Hdac1 or Hdac2 and the levels of H4K5ac. H4 was used as the loading control. KO, knockout; KD, knockdown; WT, wild type. [file 1756-8935-6-27-S2.tiff]

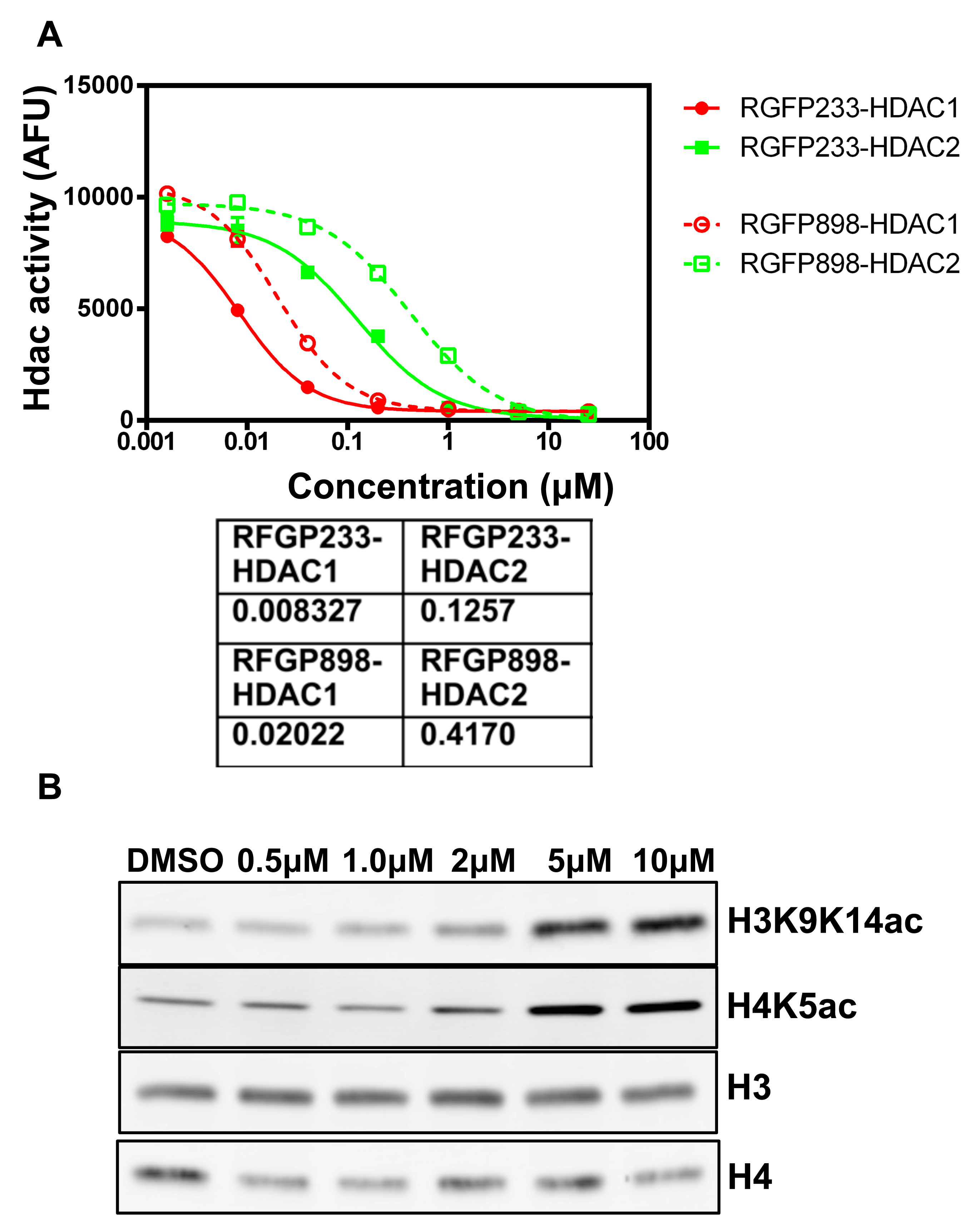

Supplement: Additional file 3: Figure S3 — Characterization of novel Hdac1,2-selective inhibitors. A. In vitro enzyme assays using recombinant HDAC proteins to determine the specificity of 233 and 898 towards Hdacs1 and 2. Numbers in the table represent IC50 values obtained for an inhibitor-enzyme combination in the in vitro HDAC assays. AFU refers to arbitrary fluorescent units. B. Dose range of RGFP898 that induces increase in histone acetylation was determined by western analysis of whole cell extracts prepared from NIH3T3 cells following treatment for 24 h with DMSO or increasing concentrations of 898 (μM). H3 and H4 levels serve as loading controls. [file 1756-8935-6-27-S3.tiff]

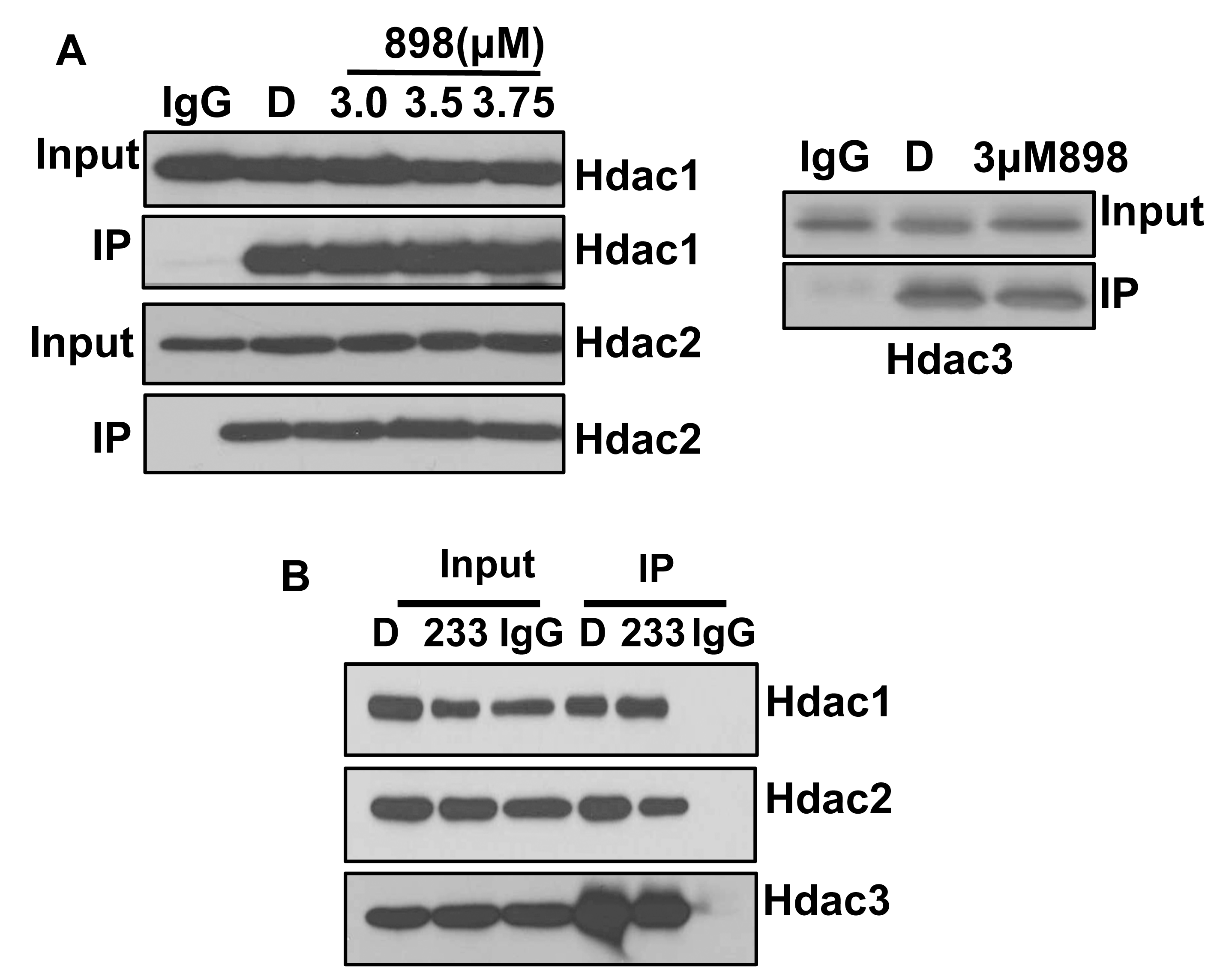

Supplement: Additional file 4: Figure S4 — Western analysis of extracts used in vivo HDAC enzyme assays. Hdac1 or Hdac2 or Hdac3 were immunoprecipitated from nuclear extracts following treatment of NIH3T3 cells with either DMSO (D), 3 μM 898 (panel A) or 3 μM 233 (panel B). Immunoprecipitated HDACs were then used in enzyme assays to measure inhibition by 898 or 233 (data shown in Figure 1D-E). Western blots were performed using antibodies against each HDAC to confirm the presence of the enzyme and the efficiency of individual immunoprecipitation. [file 1756-8935-6-27-S4.tiff]

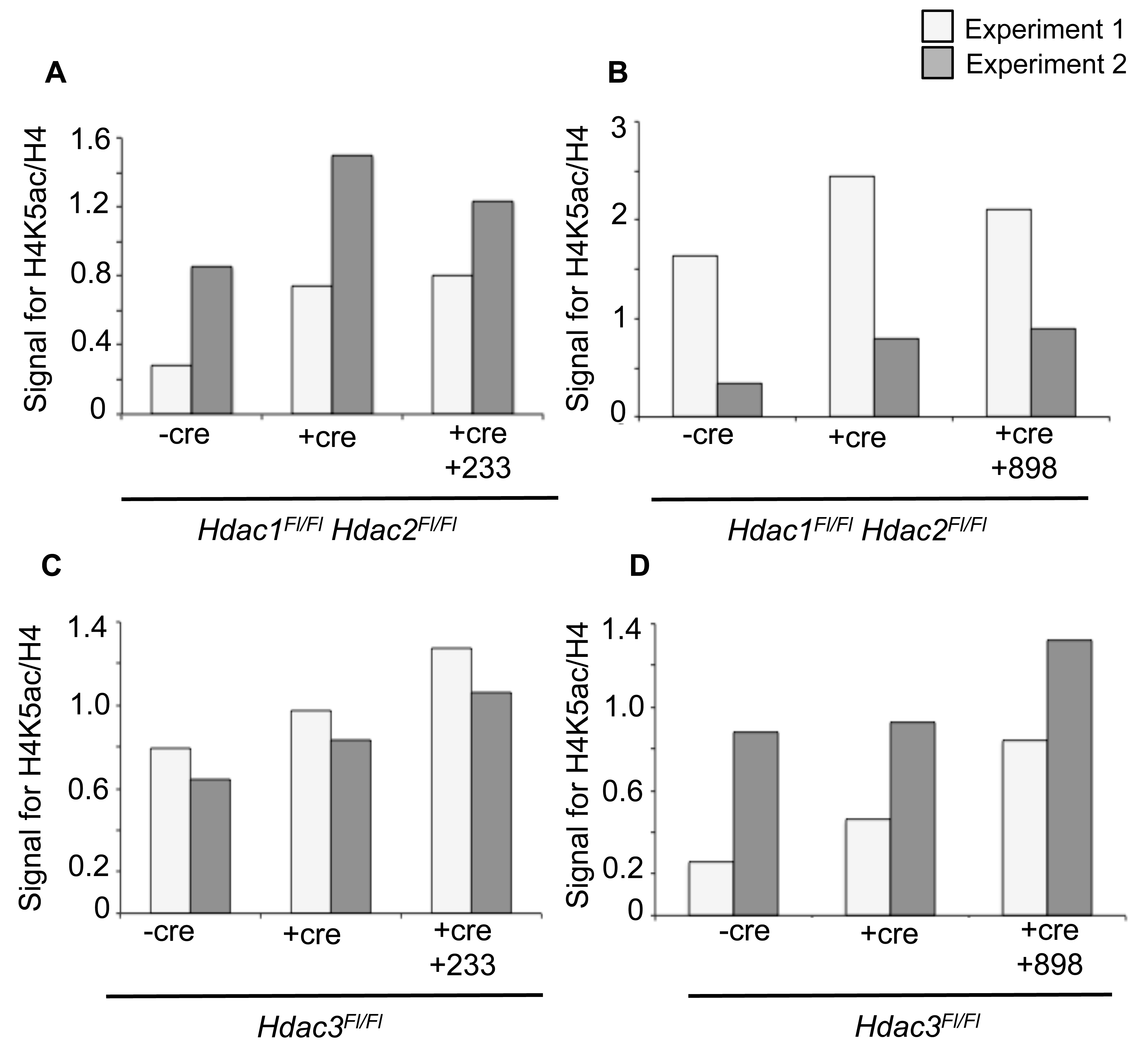

Supplement: Additional file 5: Figure S5 — Effect of 898 and 233 on H4K5ac levels in cells following conditional knockout of Hdac1,2 or Hdac3. Quantitative data for western blots shown in Figure 1: H-I. A-B. Fibrosarcoma cells containing floxed alleles of Hdac1 and Hdac2 (Hdac1Fl/FlHdac2Fl/Fl) were infected with Ad-Cre for 48 h to deplete both Hdac1 and Hdac2, and then treated with either DMSO or 3 μM 233 (A) or 3 μM 898 (B) for 24 h. C-D. Fibrosarcoma cells containing floxed alleles of Hdac3 (Hdac3Fl/Fl) were infected with Ad-Cre and treated with DMSO or 3 μM 233 (C) or 3 μM 898 (D) as described for panels A-B. Western analysis of H4K5ac and H4 was performed with whole cell lysates. For each treatment, signals for H4K5ac were normalized to the signals for H4 (loading control). Normalized signals for H4K5ac obtained from two independent experiments are shown. [file 1756-8935-6-27-S5.tiff]

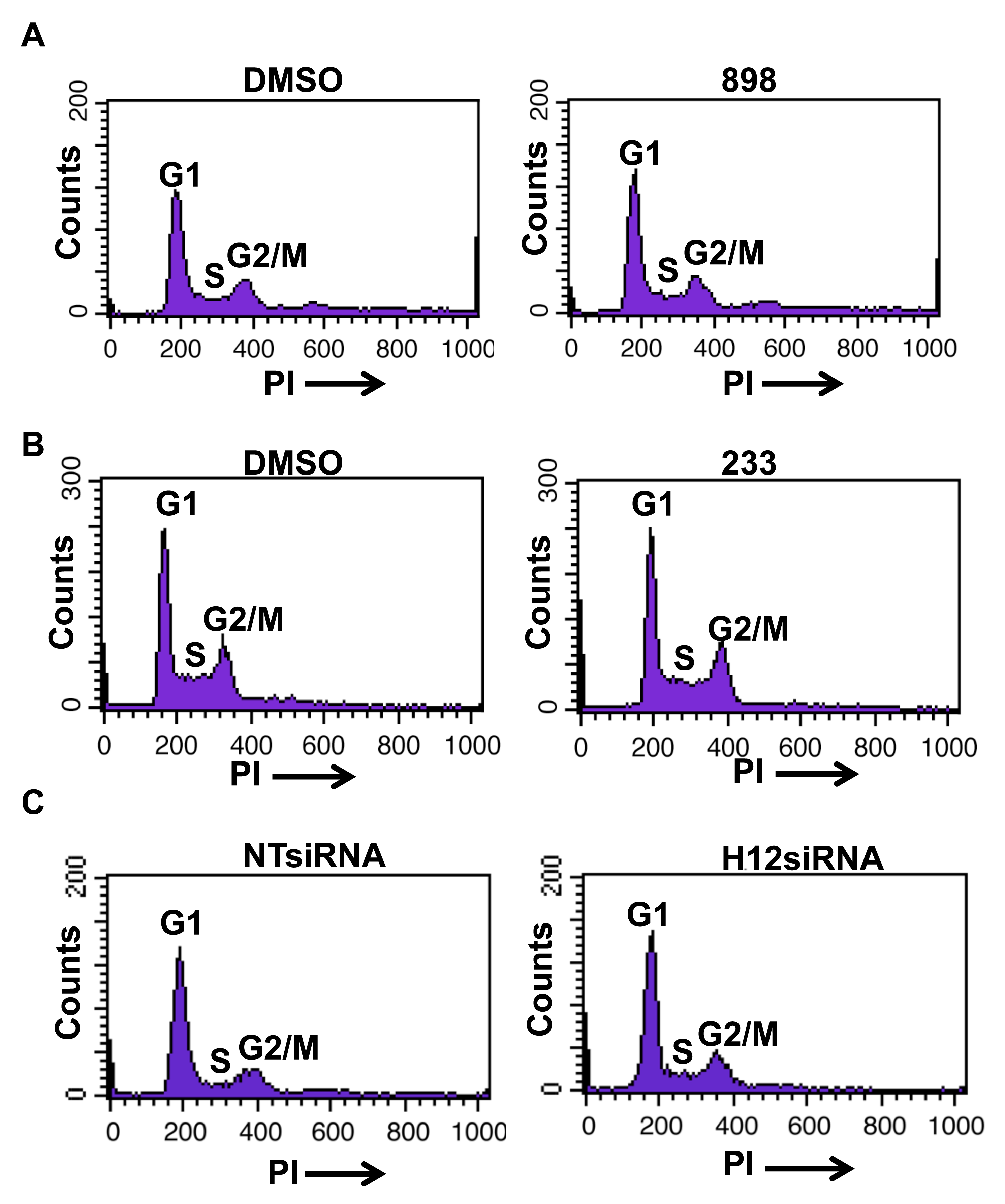

Supplement: Additional file 6: Figure S6 — Inhibition of Hdac1,2 activities for 24 h or knockdown of Hdac1,2 for 72 h does not affect the cell cycle. A and B. FACS analysis of Propidium iodide (PI) stained NIH3T3 cells treated with DMSO or 3 μM 898 (A) or 3 μM 233 (B) for 24 h. C. FACS analysis of Propidium iodide (PI) stained NIH3T3 cells transfected with either non-targeting (NT) or Hdac1,2 siRNA transfected cells at 72 h time point following transfection. Data shown is a representative of two independent experiments. [file 1756-8935-6-27-S6.tiff]

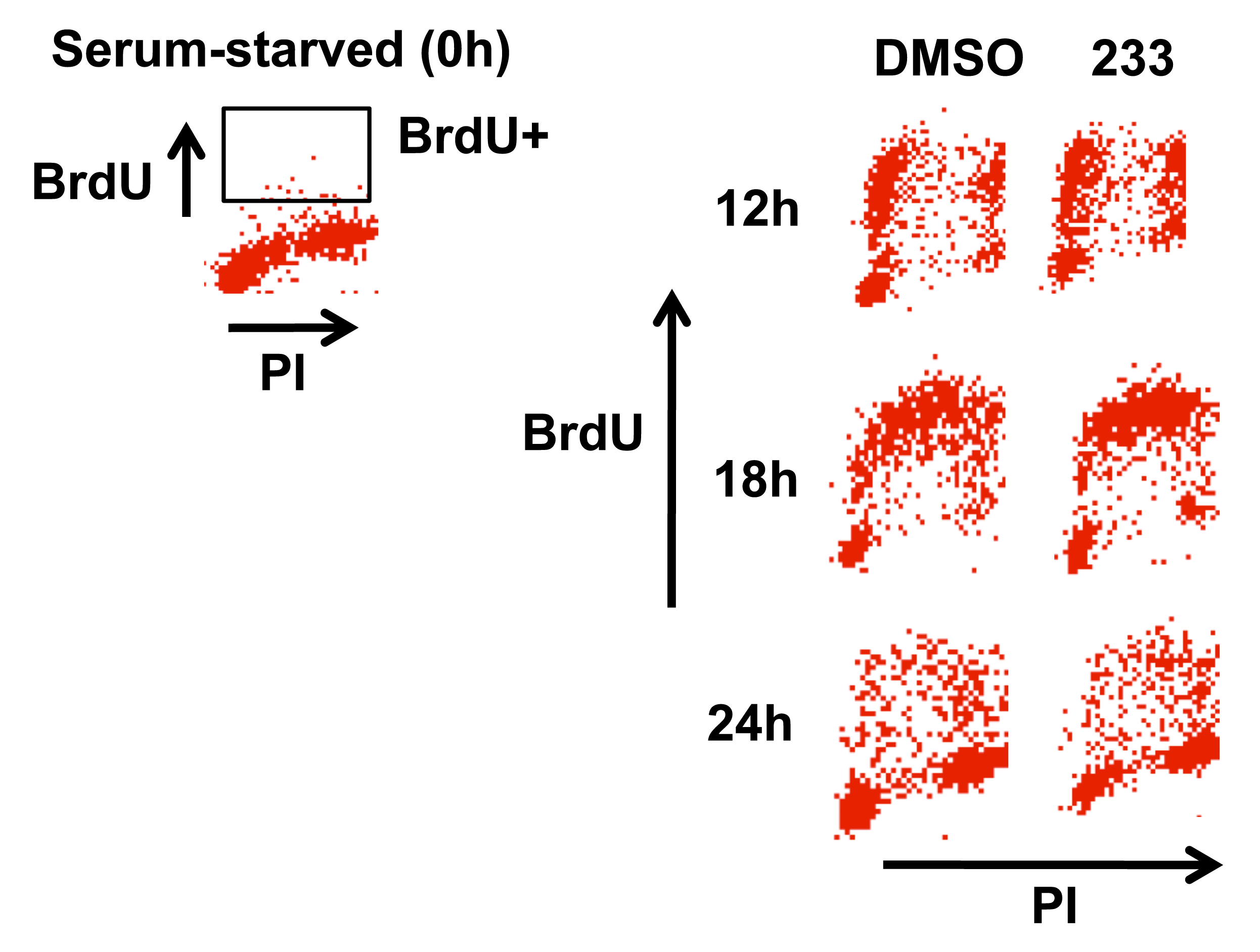

Supplement: Additional file 7: Figure S7 — Inhibition of Hdac1,2 activities with 233 does not affect S-phase progression. Serum-starved NIH3T3 cells were released into S-phase in the presence of DMSO or 3 μM 233. FACS analysis following BrdU-propidium iodide labeling and staining of cells was performed at 0 h, 12 h, 18 h and 24 h time points following release into S-phase. [file 1756-8935-6-27-S7.tiff]

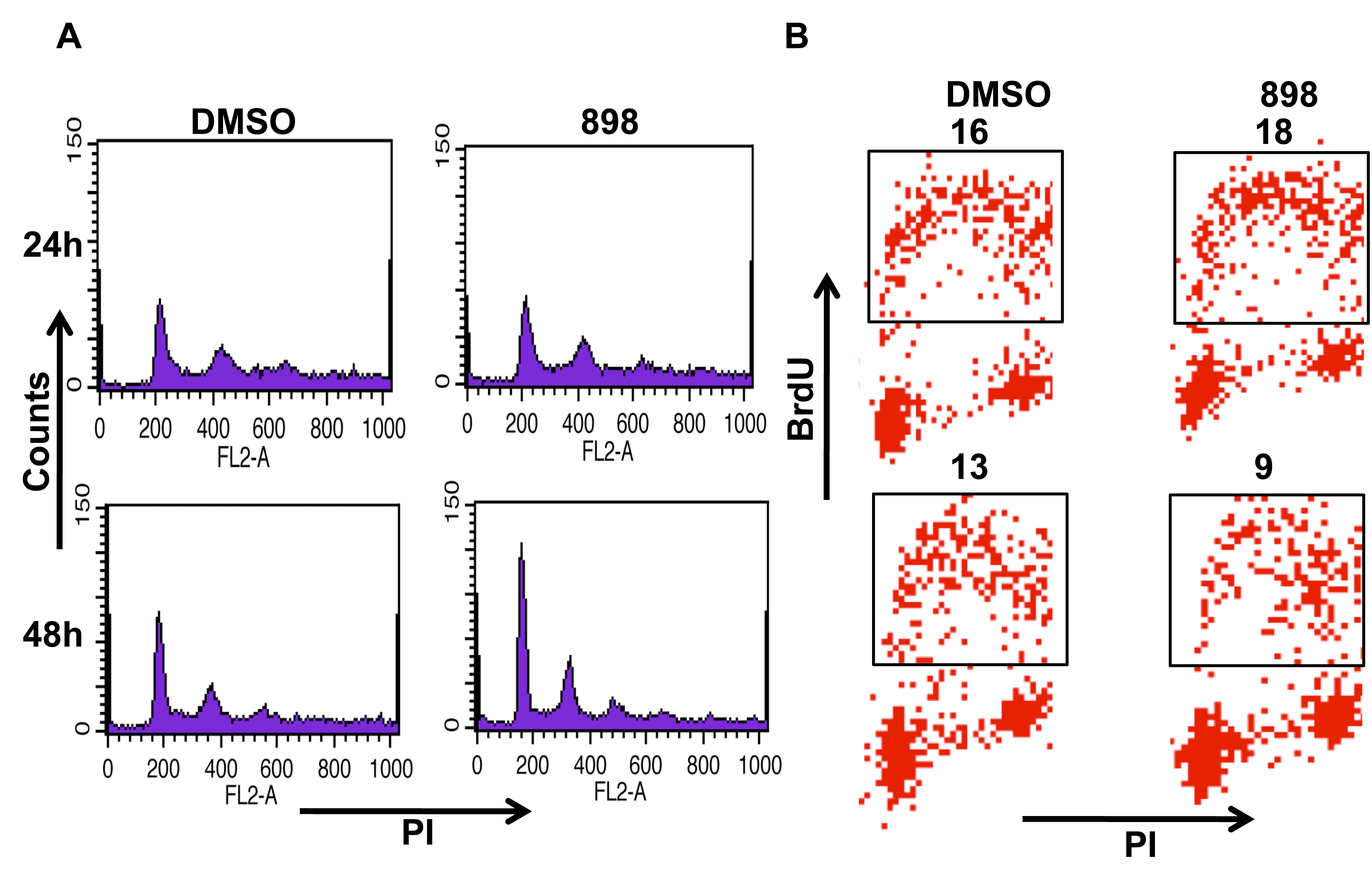

Supplement: Additional file 8: Figure S8 — Prolonged treatment of NIH3T3 cells with 898 causes a G1 arrest. A. FACS analysis following propidium iodide staining of NIH3T3 cells was performed at 24 h and 48 h time points following treatment with DMSO or 3 μM 898. B. FACS analysis of BrdU-propidium iodide stained cells following 24 h or 48 h treatment with 3 μM 898. Percentage of BrdU-positive S-phase cells is indicated. [file 1756-8935-6-27-S8.tiff]

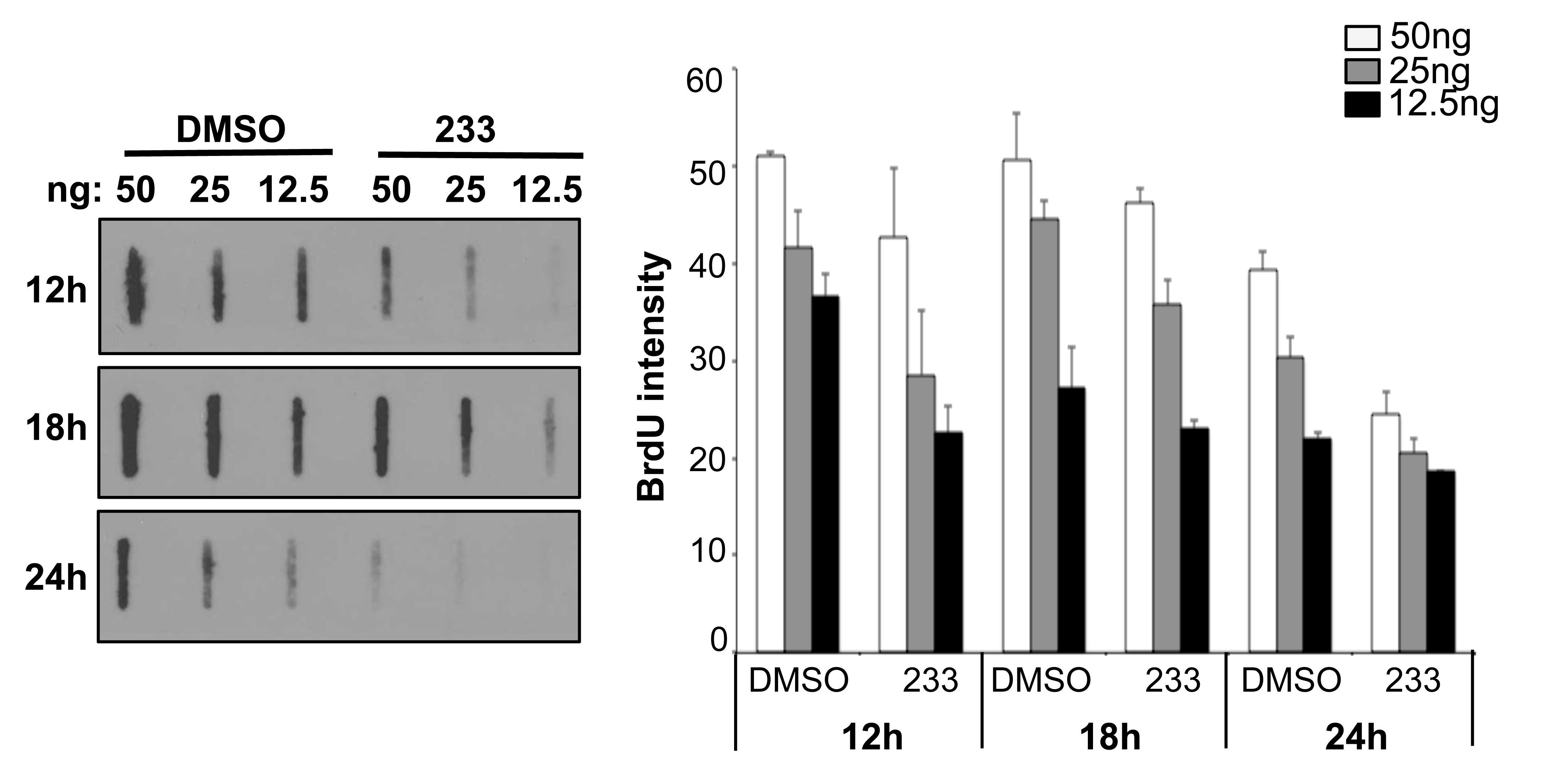

Supplement: Additional file 9: Figure S9 — BrdU incorporation is reduced following treatment with 233. Slot blot analysis of BrdU-labeled genomic DNA following treatment with 233 was performed using the indicated amounts of purified DNA. For quantitation, BrdU incorporation in various samples was measured using densitometry. Average BrdU incorporation from three independent experiments are shown. [file 1756-8935-6-27-S9.tiff]

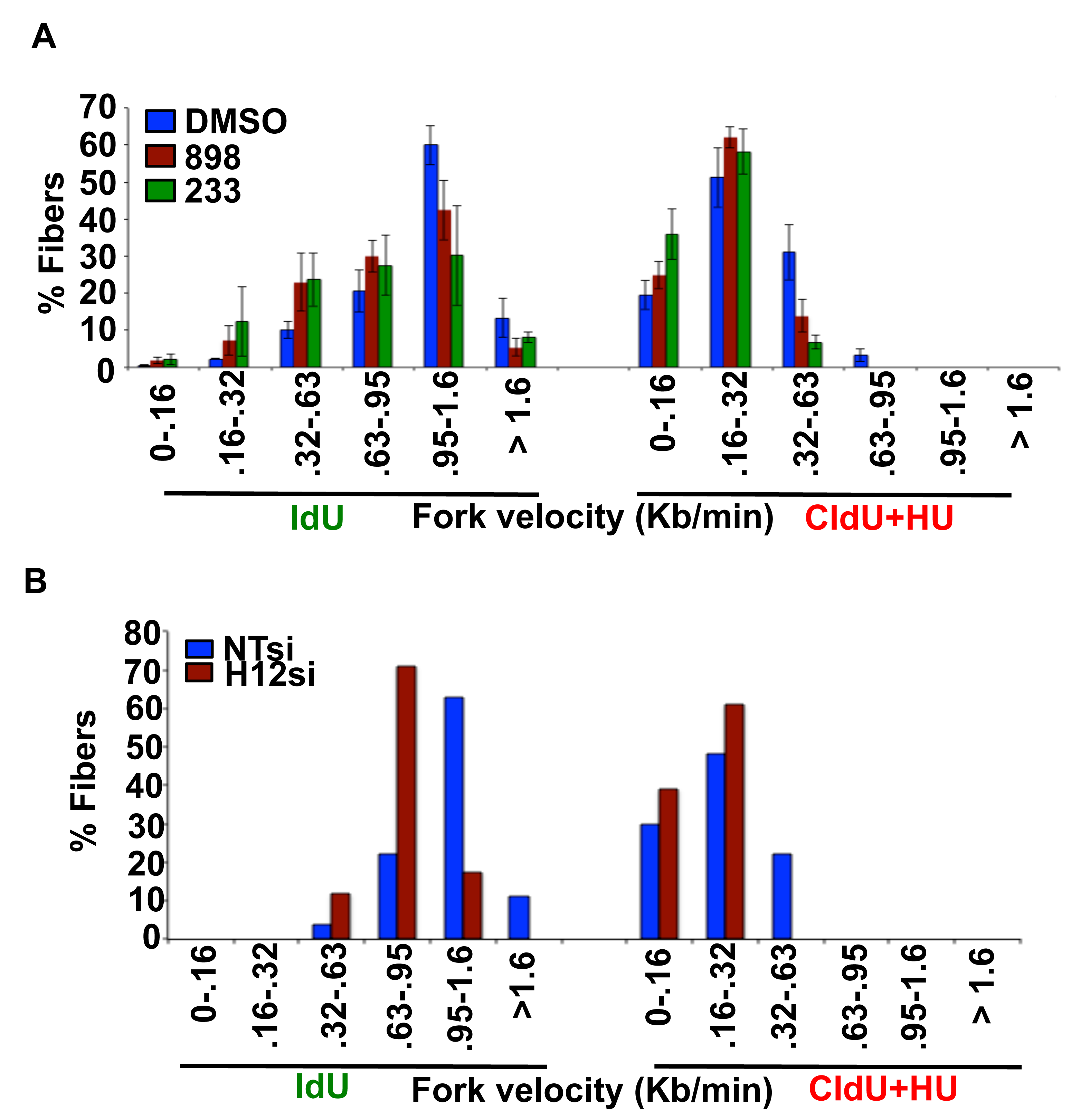

Supplement: Additional file 10: Figure S10 — Replication fork velocity is affected following treatment with Hdac1,2-selective inhibitors or knockdown of Hdacs1,2. DNA combing to measure replication fork velocity in NIH3T3 cells was performed 20 h following release into S-phase from serum starvation in the presence of DMSO or 3 μM 898 (A) or following siRNA knockdown of Hdacs1,2 (B). Cells were labeled with IdU (green) for 15 min and then with CldU (red) in the presence of 250 μM hydroxyurea for 20 min. DNA fibers were prepared and analyzed as described in the Methods section. Data represent average fork velocity of DNA fibers prepared from four independent experiments with 898 treatment or 3 independent experiments with 233. Data from one knockdown experiment is shown in the figure and the analysis was repeated twice. In each experiment, at least 100 fibers in different areas of the slide from three different slide preparations were analyzed. Error bars, standard error of the mean from independent experiments. Box plot representation of these data is shown in Figure 2C and 2D. [file 1756-8935-6-27-S10.tiff]

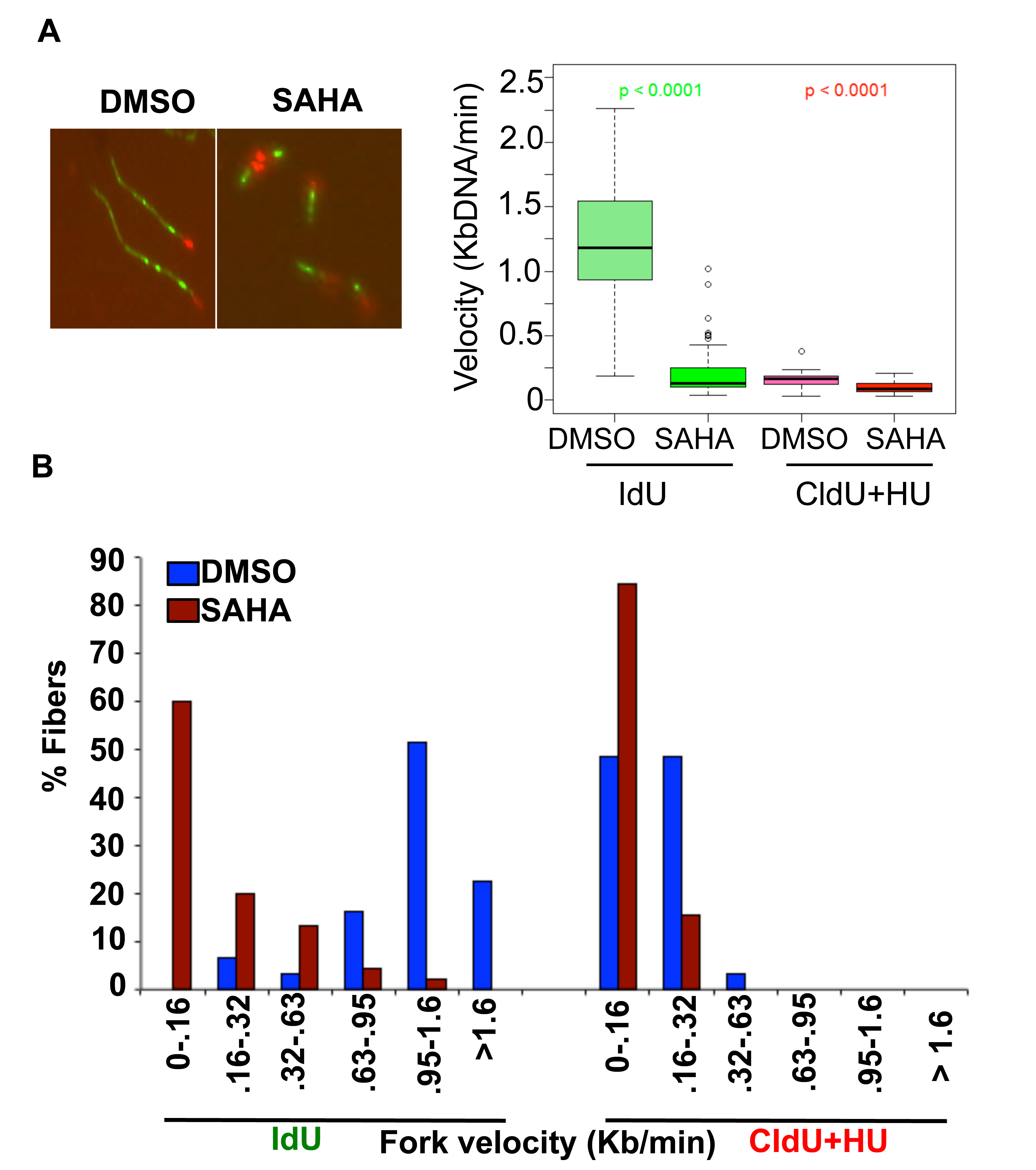

Supplement: Additional file 11: Figure S11 — Replication fork velocity is reduced upon treatment with SAHA. A. NIH3T3 cells were treated with either DMSO or 10 μM SAHA for 24 h. Fibers were prepared and analyzed as described earlier in the methods section. Box-plot analysis was done with data obtained from two independent experiments. B. Percentage fibers with varying velocities are shown in panel B. Graph shows data from a representative experiment. In each experiment, at least 100 fibers in different areas of the slide from three different slide preparations were analyzed. [file 1756-8935-6-27-S11.tiff]

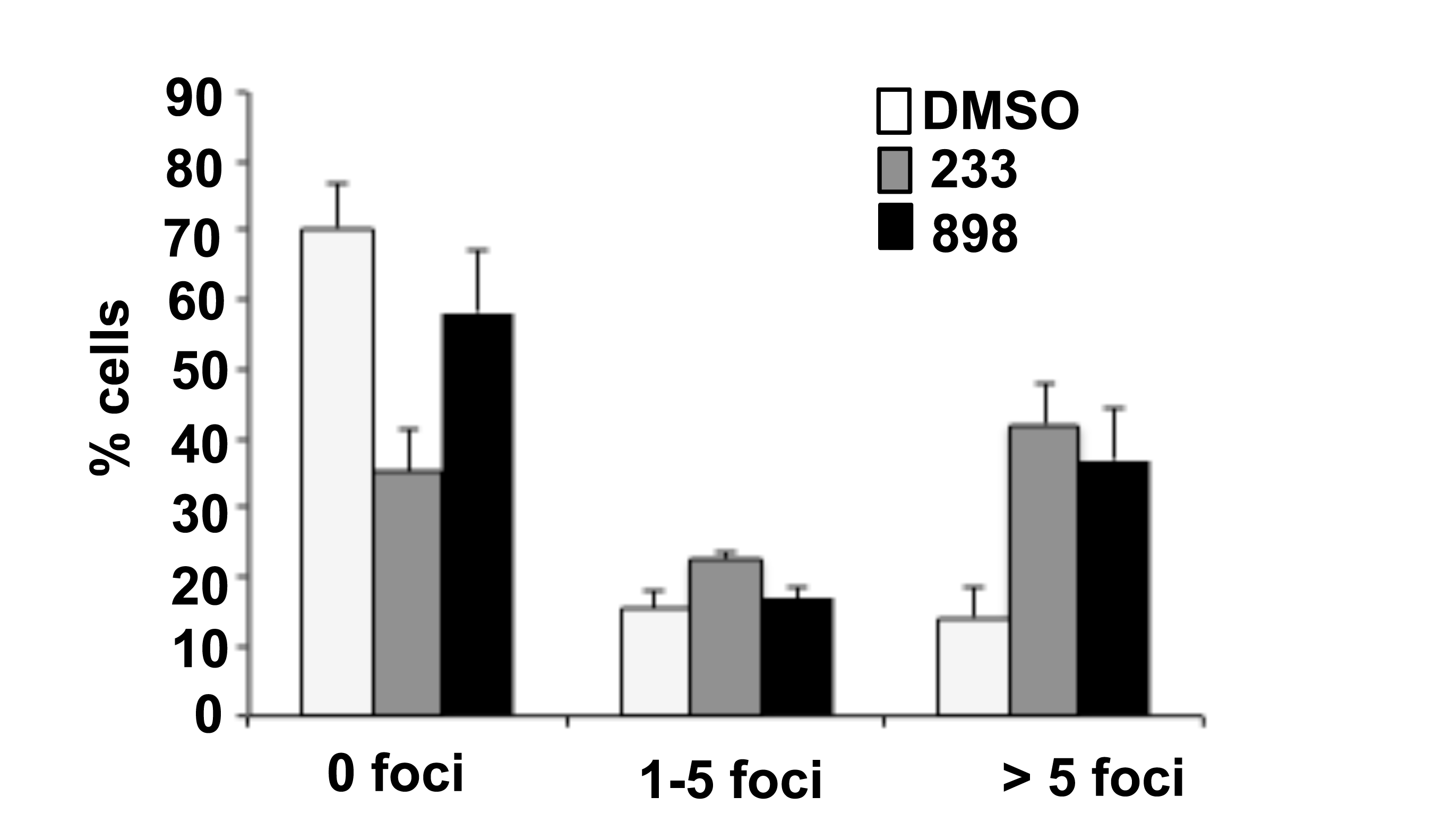

Supplement: Additional file 12: Figure S12 — Quantitation of γH2AX foci in NIH3T3 cells following treatment with 3 μM 898 or 3 μM 233 for 48 hr. Cells with various amount of big and bright foci in a population of 100 cells were counted. Average percent of cells for each category obtained from four independent experiments is shown. Representative immunofluorescence image of γH2AX foci in the nuclei of control and Hdac1,2 inhibitor-treated cells is shown in Figure 3A. [file 1756-8935-6-27-S12.tiff]

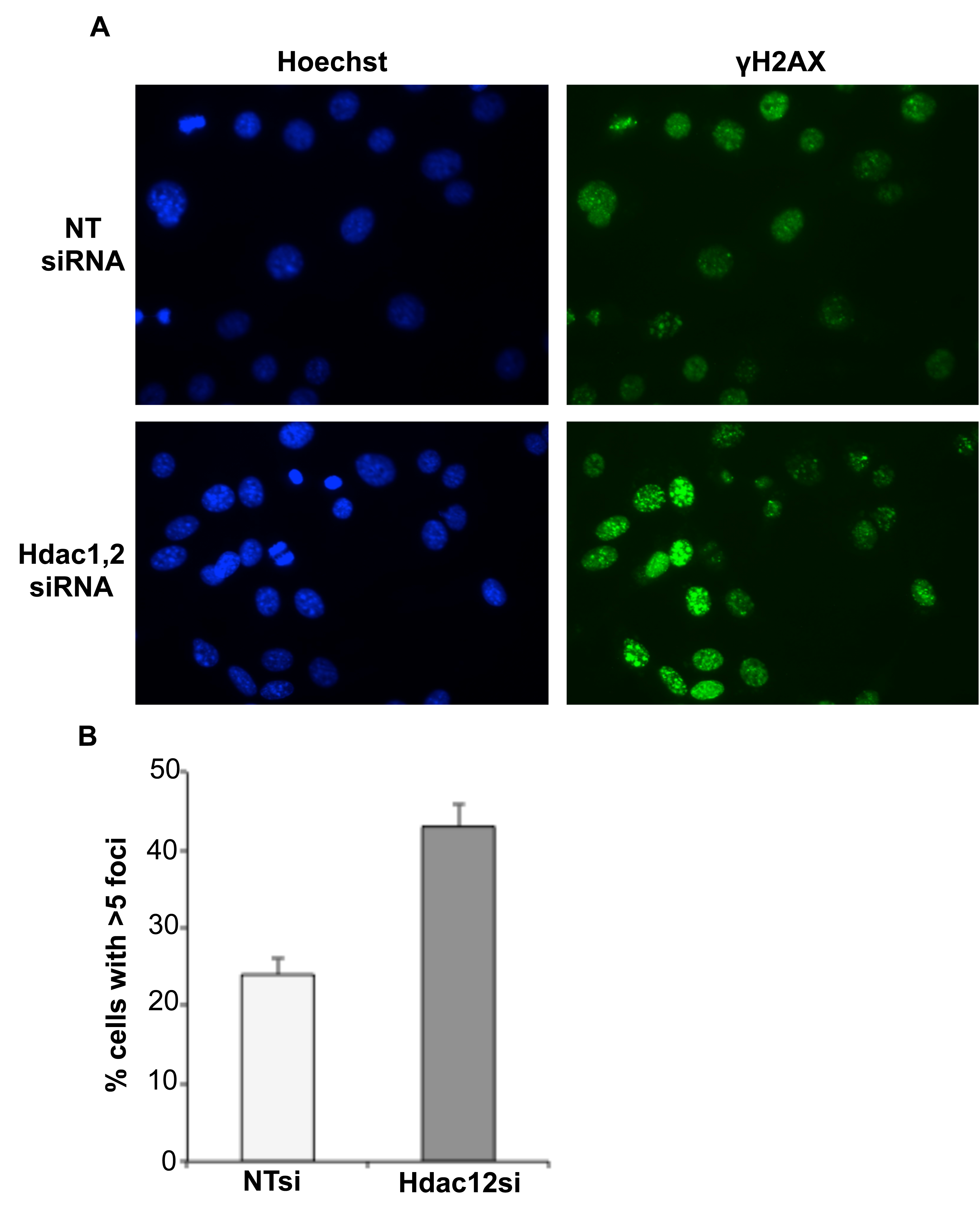

Supplement: Additional file 13: Figure S13 — DNA damage response is activated following knockdown of Hdacs1,2. DNA damage in non-targeting (NT) siRNA or Hdacs1,2-siRNA transfected NIH3T3 cells was measured at 72 h time point by immunofluorescence analysis by staining for γH2AX. Hoechst staining was performed to visualize nuclei. Cells with >5 γH2AX foci in a population of 100 cells were counted. Average of two independent experiments is shown. [file 1756-8935-6-27-S13.tiff]

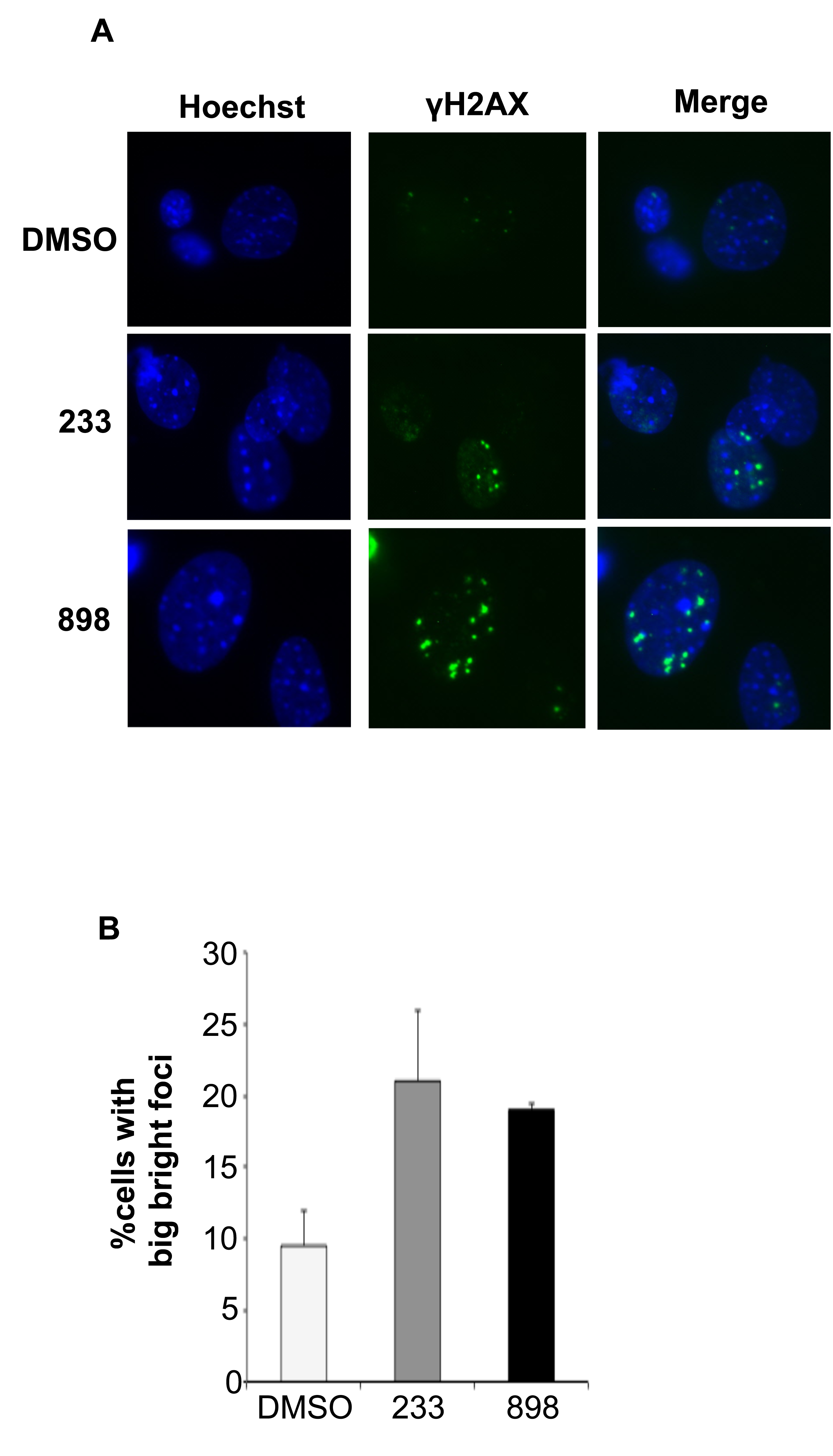

Supplement: Additional file 14: Figure S14 — DNA damage response is activated in primary mouse embryo fibroblast cells following 898 or 233 treatment. Wild-type mouse embryo fibroblasts (MEF) isolated from 13.5 (p.c.) embryos were treated with 3 μM 898 or 3 μM 233 for 48 hr. Immunofluorescence of γH2AX was performed and percentage of cells with big bright foci were determined in two independent experiments. Majority of cells had 1–5 foci. Data represents average number of cells with γH2AX foci obtained from two different MEF preparations. [file 1756-8935-6-27-S14.tiff]

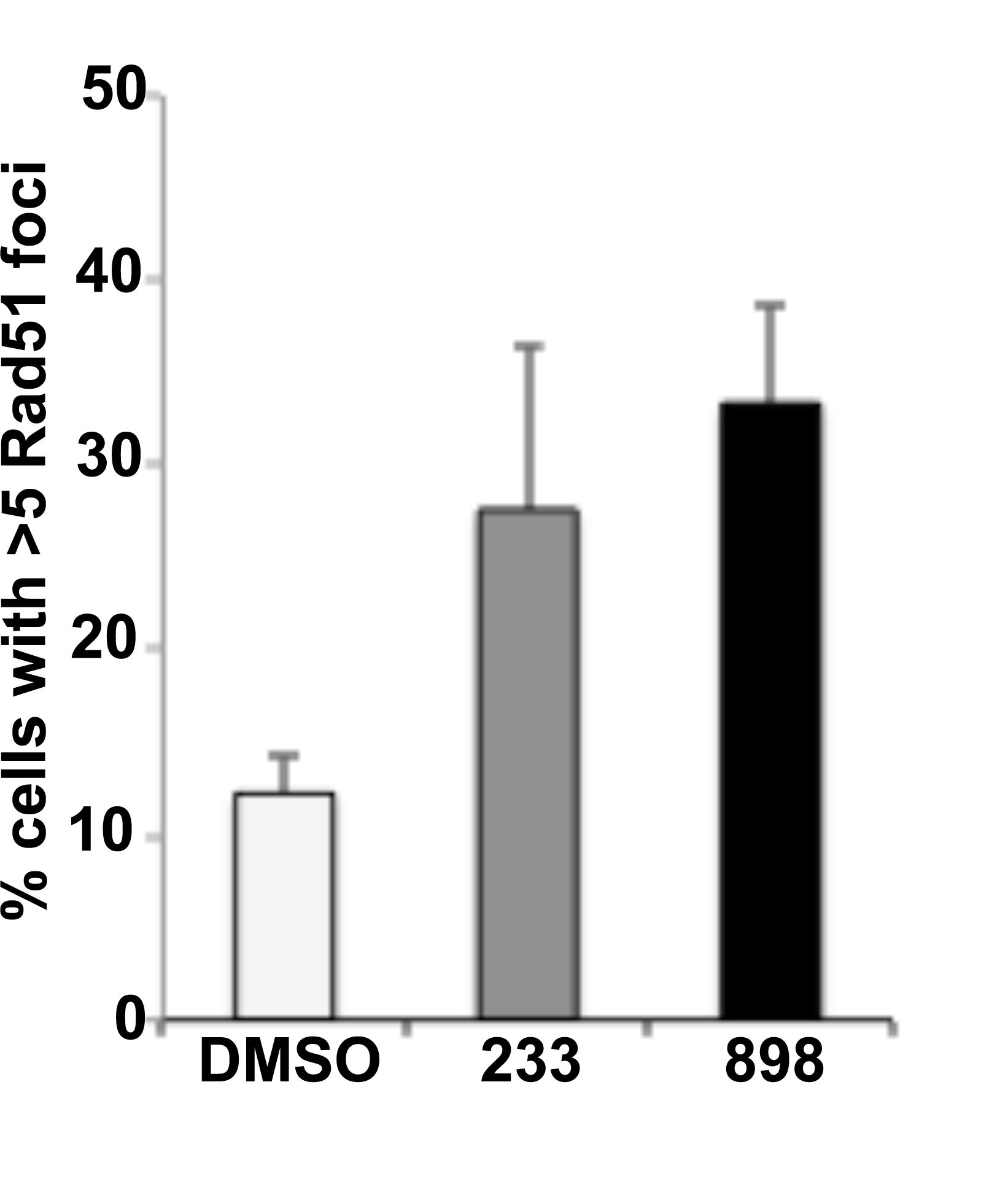

Supplement: Additional file 15: Figure S15 — Quantitation of Rad51 foci formed in S-phase cells following inhibition of Hdacs1,2. Serum-starved NIH3T3 cells were released into S-phase and treated with either DMSO or 3 μM 233 or 3 μM 898 in the presence of 500 μM hydroxyurea. Immunofluorescence staining for Rad51 was performed and percentage of cells with greater than 5 foci were determined from two independent experiments, with each experiment containing duplicate treatments. Representative immunofluorescence images with Rad51 foci are shown in Figure 3B. [file 1756-8935-6-27-S15.tiff]

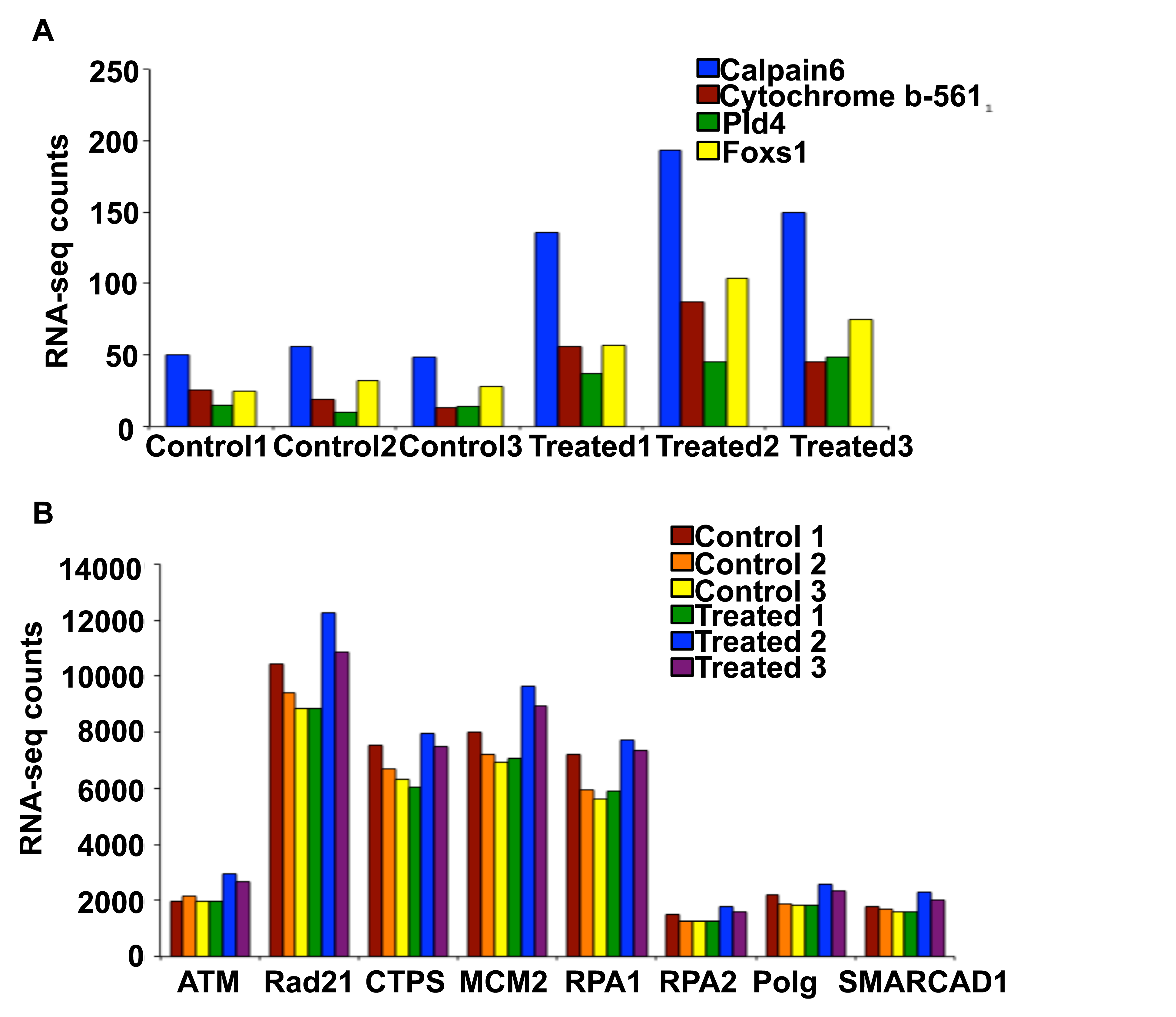

Supplement: Additional file 16: Figure S16. — Inhibition of Hdac1,2 activities in S-phase cells does not alter expression of genes coding for replication-associated factors. Serum-starved NIH3T3s were released into S-phase in the presence of DMSO or 3 μM 898 for 20 h. Three independent DMSO and 898 treatments were performed for total RNA isolation. Purified RNA was subjected to RNA-seq analyses. A. Four genes that were consistently up-regulated in all three 898 treated samples compared to the DMSO control. B. Levels of DNA damage-associated genes (ATM, Rad21) and replication-associated genes (MCM2, CTPS, RPA1, RPA2, DNA polymerase γ (Polg) and SMARCAD1) remain unchanged in all the three 898-treated samples compared to DMSO control. [file 1756-8935-6-27-S16.tiff]

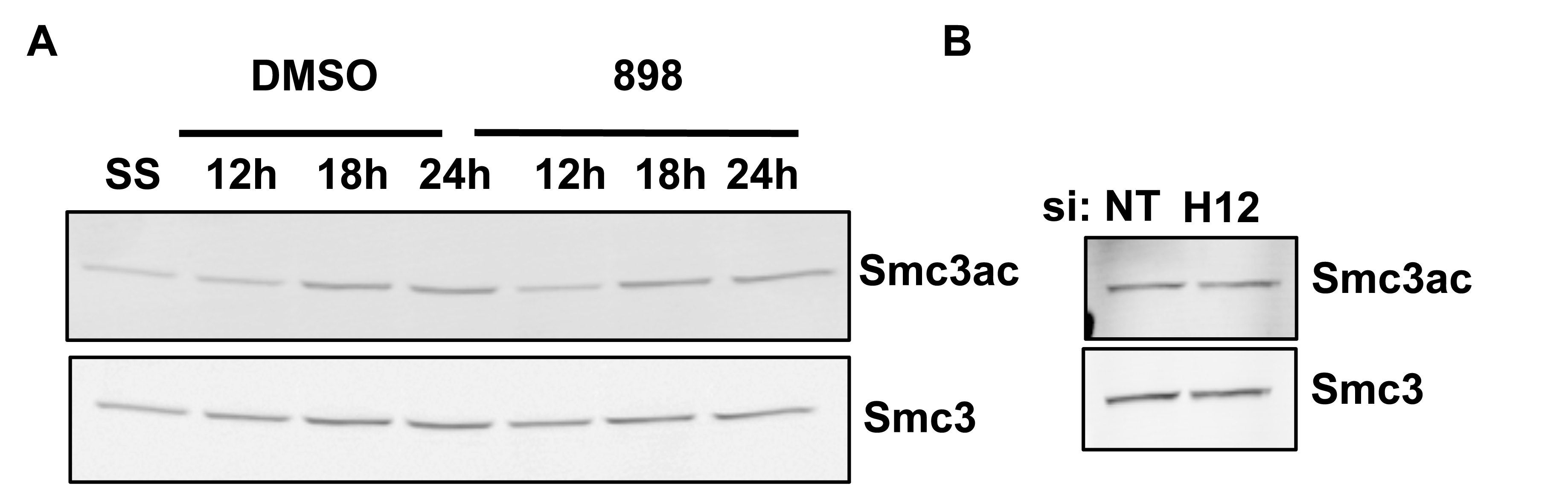

Supplement: Additional file 18: Figure S17 — Hdacs1,2 do not target Smc3 acetylation. A. Chromatin from serum-starved NIH3T3 cells that were released into S-phase in the presence of DMSO or 3 μM 898 for 0 h, 12 h, 18 h and 24 h was prepared for western analysis. Smc3 levels serve as the loading control. B. NIH3T3 cells were transfected with either non-targeting (NT) or Hdac1,2 (H12) siRNA prior to chromatin extract preparation, and levels of Smc3ac and Smc3 were examined using western analysis. [file 1756-8935-6-27-S18.tiff]

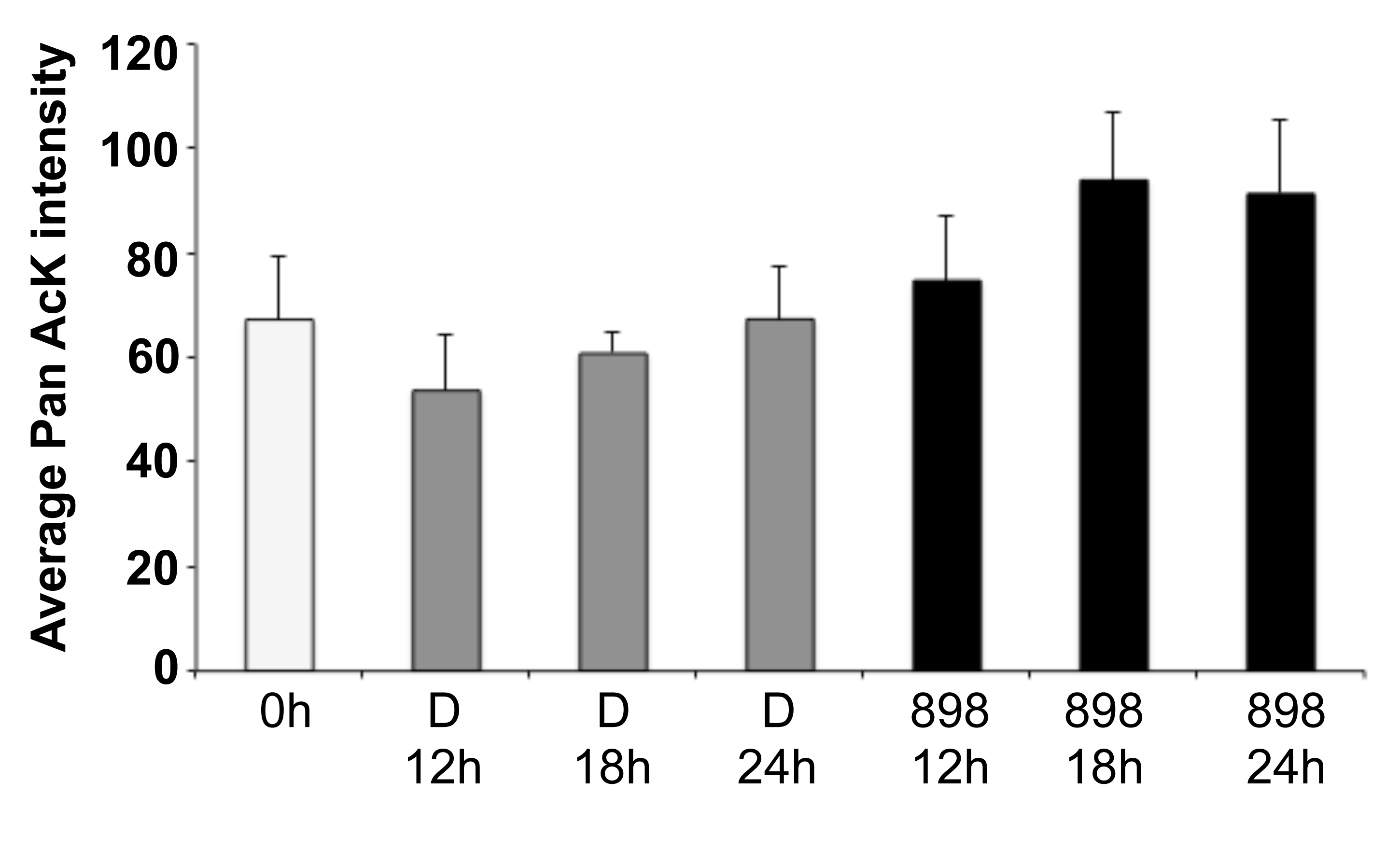

Supplement: Additional file 19: Figure S18 — Inhibition of Hdac1,2 activities increases histone acetylation in S-phase cells. Quantitation of the western blot performed with pan-acetyllysine (pan-AcK) antibody shown in Figure 5B. Chromatin was prepared from NIH3T3 cells following release of cells into S-phase from serum starvation and western blot analysis with pan-AcK antibody was done to look at histone acetylation. Signals obtained for histones H3, H2A, H2B and H4 were measured by densitometry. Data represents cumulative signal for all four histones and the average signal for histone +/− standard error from three independent experiments is shown. [file 1756-8935-6-27-S19.tiff]

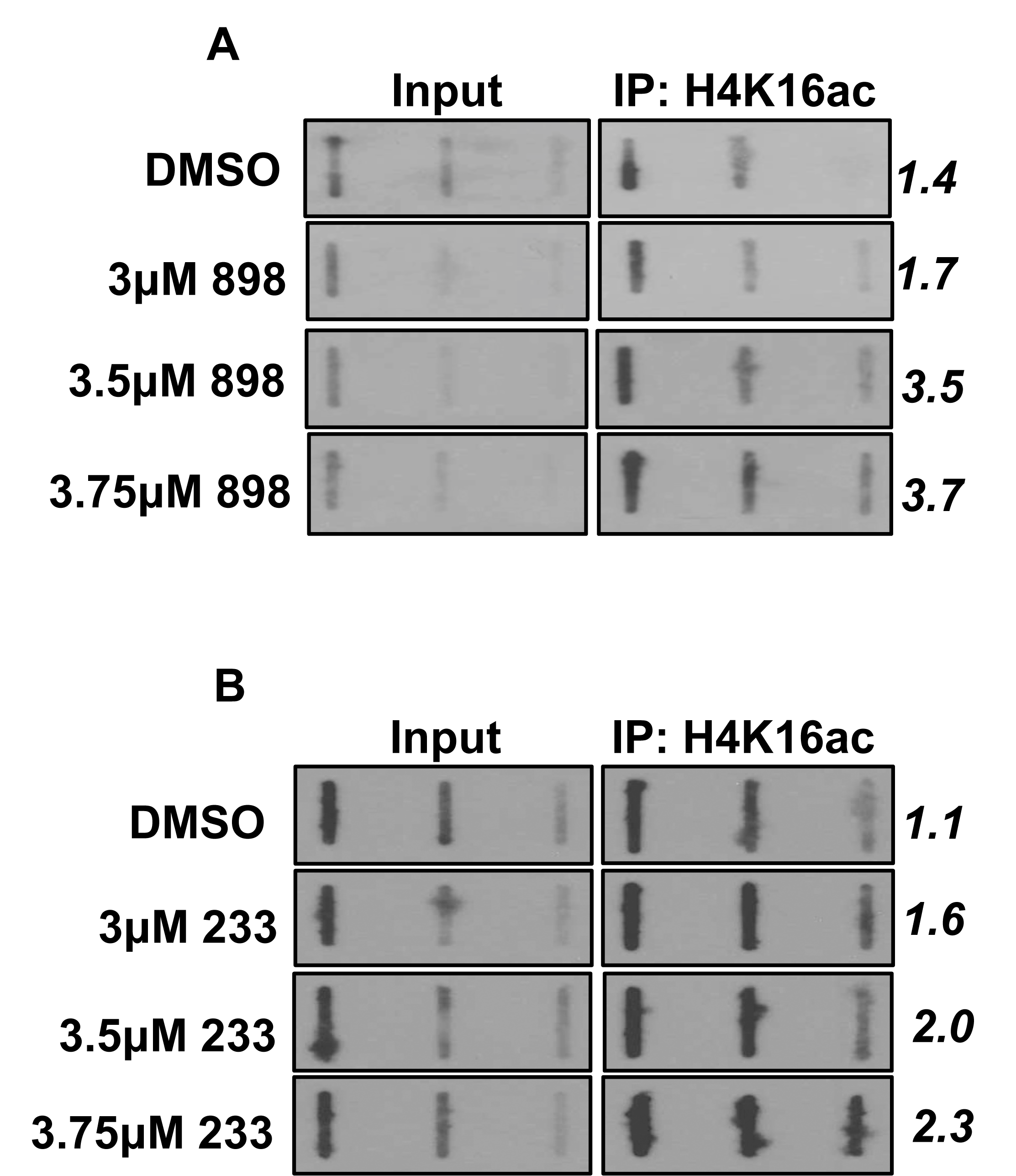

Supplement: Additional file 20: Figure S19 — Loss of Hdacs1,2 increases H4K16ac on nascent chromatin. ChIP of H4K16ac was done and increasing volumes of ChIP DNA were spotted onto a membrane using Slot Blot and probed with anti-BrdU antibody. In A-B, a representative blot shown and the experiment with 3.75 μM Hdac1,2 inhibitor and siRNAs was repeated at least 3 times. Average BrdU signal of high and medium volume of ChIP DNA spotted on the slot was quantified by the Image J software and is shown on the right hand side of the figure. [file 1756-8935-6-27-S20.tiff]

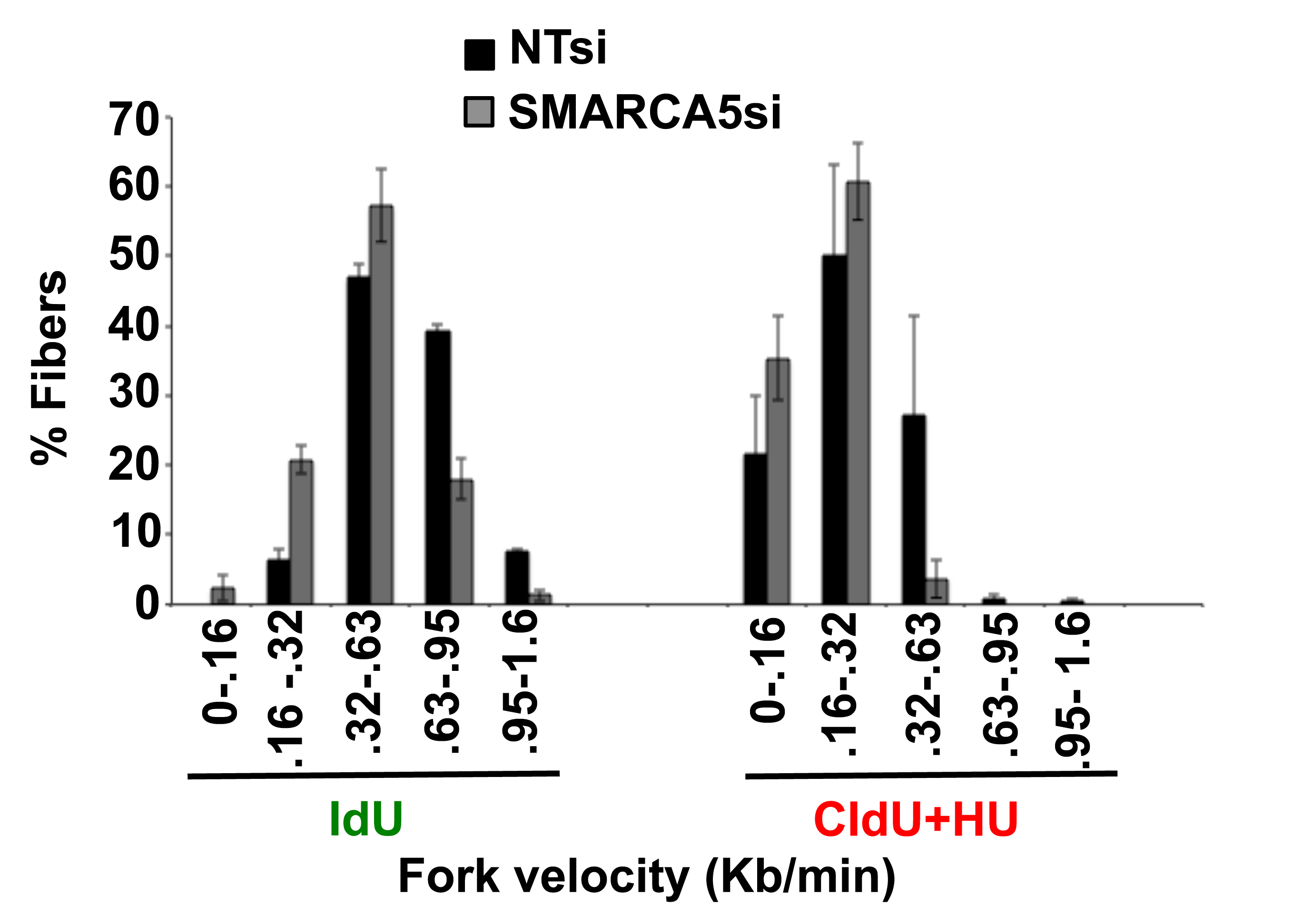

Supplement: Additional file 21: Figure S20 — Knockdown of SMARCA5 reduces replication fork velocity. Replication fork velocity was measured in HeLa cells transfected with either non-targeting siRNA (NTsi) or SMARCA5 siRNA at 72 h time point following transfection. Data represent average fork velocity calculated from three independent experiments. Error bars, standard error of the mean. [file 1756-8935-6-27-S21.tiff]

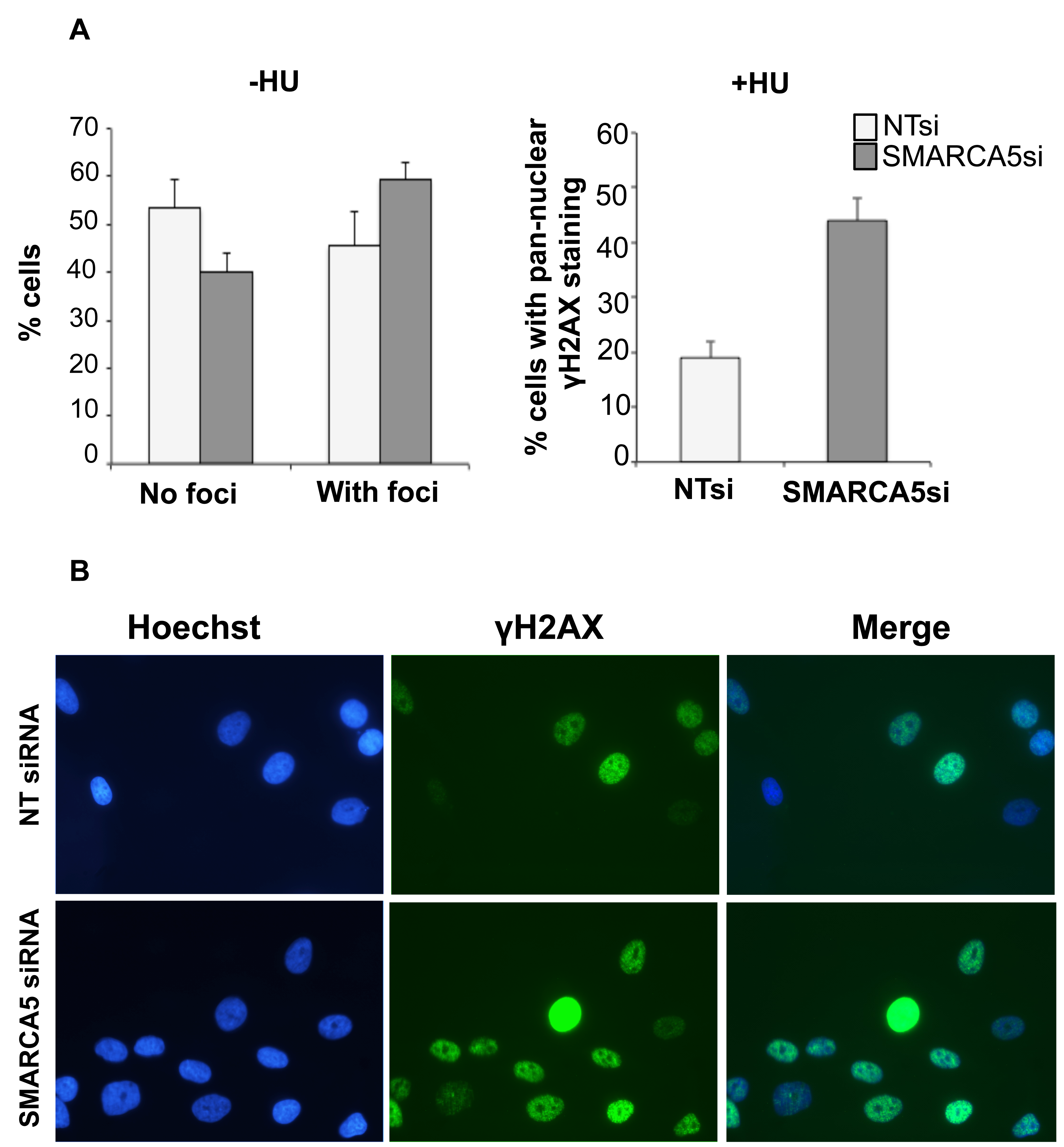

Supplement: Additional file 22: Figure S21 — DNA damage response is activated in HeLa cells following knockdown of SMARCA5. A. HeLa cells were transfected with non-targeting (NT) or SMARCA5 siRNA and immunofluorescence was performed to measure γH2AX foci formation. A. Quantitation of percentage of cells with γH2AX foci in control (non-targeting siRNA transfected) or SMARCA5 siRNA transfected cells in the absence of hydroxyurea (HU) is shown in the left panel and cells with pan-nuclear γH2AX staining in control (non-targeting siRNA transfected) or SMARCA5 siRNA transfected cells in the presence of hydroxyurea (HU) is shown in the right panel. Average data from two independent siRNA transfections are shown. B. Representative images for pan-nuclear γH2AX staining in control (non-targeting siRNA-transfected) or SMARCA5 siRNA-transfected cells in the presence of hydroxyurea are shown. [file 1756-8935-6-27-S22.tiff]
