# Supplementary material for: Histone deacetylases 1 and 2 maintain S-phase chromatin and DNA replication fork progression
Source: Epigenetics Chromatin. 2013 Aug 15;6:27. doi: 10.1186/1756-8935-6-27 (PMC3765969; doi:10.1186/1756-8935-6-27)
Supplement: Additional file 17: Table S1 — List of genes that are differentially expressed in S-phase NIH3T3 cells following 3 μM 898 treatment. [file 1756-8935-6-27-S17.docx]

**Additional File 17: Table S1**

**Genes that are differentially expressed in S-phase 3T3 cells following 3μM 898 treatment**

| GeneName | Log2Ratio | GO Term Accession |
| --- | --- | --- |
| Il12rb1 | 1.0392818 | GO:0032729 |
| Il12rb1 | 1.0392818 | GO:0042104 |
| Il12rb1 | 1.0392818 | GO:0018108 |
| Il12rb1 | 1.0392818 | GO:0071346 |
| Il12rb1 | 1.0392818 | GO:0035722 |
| Il12rb1 | 1.0392818 | GO:0038155 |
| Il12rb1 | 1.0392818 | GO:0009897 |
| Il12rb1 | 1.0392818 | GO:0042022 |
| Il12rb1 | 1.0392818 | GO:0072536 |
| Il12rb1 | 1.0392818 | GO:0005143 |
| Il12rb1 | 1.0392818 | GO:0016517 |
| Il12rb1 | 1.0392818 | GO:0042019 |
| Il12rb1 | 1.0392818 | GO:0042020 |
| Il12rb1 | 1.0392818 | GO:0019955 |
| Il12rb1 | 1.0392818 | GO:0005515 |
| B3gnt3 | 1.8244967 | GO:0006486 |
| B3gnt3 | 1.8244967 | GO:0008150 |
| B3gnt3 | 1.8244967 | GO:0000139 |
| B3gnt3 | 1.8244967 | GO:0005575 |
| B3gnt3 | 1.8244967 | GO:0016021 |
| B3gnt3 | 1.8244967 | GO:0016020 |
| B3gnt3 | 1.8244967 | GO:0003674 |
| B3gnt3 | 1.8244967 | GO:0008378 |
| Hcrtr1 | 1.0169458 | GO:0007218 |
| Hcrtr1 | 1.0169458 | GO:0007186 |
| Hcrtr1 | 1.0169458 | GO:0005886 |
| Hcrtr1 | 1.0169458 | GO:0016021 |
| Hcrtr1 | 1.0169458 | GO:0016499 |
| Hcrtr1 | 1.0169458 | GO:0017046 |
| Mrvi1 | 1.2826147 | GO:0016021 |
| Mrvi1 | 1.2826147 | GO:0016529 |
| Mrvi1 | 1.2826147 | GO:0048471 |
| Apoa5 | 1.0056314 | GO:0006641 |
| Apoa5 | 1.0056314 | GO:0006869 |
| Apoa5 | 1.0056314 | GO:0010898 |
| Apoa5 | 1.0056314 | GO:0010902 |
| Apoa5 | 1.0056314 | GO:0009725 |
| Apoa5 | 1.0056314 | GO:0045723 |
| Apoa5 | 1.0056314 | GO:0031100 |
| Apoa5 | 1.0056314 | GO:0034370 |
| Apoa5 | 1.0056314 | GO:0042632 |
| Apoa5 | 1.0056314 | GO:0042157 |
| Apoa5 | 1.0056314 | GO:0042246 |
| Apoa5 | 1.0056314 | GO:0019433 |
| Apoa5 | 1.0056314 | GO:0051006 |
| Apoa5 | 1.0056314 | GO:0055090 |
| Apoa5 | 1.0056314 | GO:0070328 |
| Apoa5 | 1.0056314 | GO:0050996 |
| Apoa5 | 1.0056314 | GO:0034361 |
| Apoa5 | 1.0056314 | GO:0034364 |
| Apoa5 | 1.0056314 | GO:0042627 |
| Apoa5 | 1.0056314 | GO:0005576 |
| Apoa5 | 1.0056314 | GO:0005615 |
| Apoa5 | 1.0056314 | GO:0008201 |
| Apoa5 | 1.0056314 | GO:0031210 |
| Apoa5 | 1.0056314 | GO:0019899 |
| Apoa5 | 1.0056314 | GO:0050750 |
| Apoa5 | 1.0056314 | GO:0060230 |
| Apoa5 | 1.0056314 | GO:0008289 |
| Apoa5 | 1.0056314 | GO:0005515 |
| Apoa5 | 1.0056314 | GO:0005543 |
| Apoa5 | 1.0056314 | GO:0008047 |
| Apoa5 | 1.0056314 | GO:0035473 |
| Apoa5 | 1.0056314 | GO:0070325 |
| Apoa5 | 1.0056314 | GO:0060229 |
| Gm872 | 1.1818471 |  |
| Erbb3 | 1.4308434 | GO:0003197 |
| Erbb3 | 1.4308434 | GO:0007169 |
| Erbb3 | 1.4308434 | GO:0007162 |
| Erbb3 | 1.4308434 | GO:0007422 |
| Erbb3 | 1.4308434 | GO:0007409 |
| Erbb3 | 1.4308434 | GO:0007519 |
| Erbb3 | 1.4308434 | GO:0009611 |
| Erbb3 | 1.4308434 | GO:0014037 |
| Erbb3 | 1.4308434 | GO:0009968 |
| Erbb3 | 1.4308434 | GO:0014065 |
| Erbb3 | 1.4308434 | GO:0007623 |
| Erbb3 | 1.4308434 | GO:0043524 |
| Erbb3 | 1.4308434 | GO:0043586 |
| Erbb3 | 1.4308434 | GO:0032869 |
| Erbb3 | 1.4308434 | GO:0042127 |
| Erbb3 | 1.4308434 | GO:0042476 |
| Erbb3 | 1.4308434 | GO:0042493 |
| Erbb3 | 1.4308434 | GO:0021545 |
| Erbb3 | 1.4308434 | GO:0051048 |
| Erbb3 | 1.4308434 | GO:0051402 |
| Erbb3 | 1.4308434 | GO:0060056 |
| Erbb3 | 1.4308434 | GO:0046326 |
| Erbb3 | 1.4308434 | GO:0006468 |
| Erbb3 | 1.4308434 | GO:0007165 |
| Erbb3 | 1.4308434 | GO:0061098 |
| Erbb3 | 1.4308434 | GO:0005615 |
| Erbb3 | 1.4308434 | GO:0005634 |
| Erbb3 | 1.4308434 | GO:0016021 |
| Erbb3 | 1.4308434 | GO:0016323 |
| Erbb3 | 1.4308434 | GO:0016324 |
| Erbb3 | 1.4308434 | GO:0016328 |
| Erbb3 | 1.4308434 | GO:0045211 |
| Erbb3 | 1.4308434 | GO:0016020 |
| Erbb3 | 1.4308434 | GO:0004714 |
| Erbb3 | 1.4308434 | GO:0004716 |
| Erbb3 | 1.4308434 | GO:0005515 |
| Erbb3 | 1.4308434 | GO:0005524 |
| Erbb3 | 1.4308434 | GO:0030296 |
| Erbb3 | 1.4308434 | GO:0019838 |
| Erbb3 | 1.4308434 | GO:0046982 |
| Erbb3 | 1.4308434 | GO:0004672 |
| Erbb3 | 1.4308434 | GO:0016772 |
| Erbb3 | 1.4308434 | GO:0004713 |
| Erbb3 | 1.4308434 | GO:0005515 |
| Serpinb1b | 1.124999 | GO:0030162 |
| Serpinb1b | 1.124999 | GO:0042176 |
| Serpinb1b | 1.124999 | GO:0005737 |
| Serpinb1b | 1.124999 | GO:0005615 |
| Serpinb1b | 1.124999 | GO:0004867 |
| Rinl | 1.0305028 | GO:0006897 |
| Rinl | 1.0305028 | GO:0001726 |
| Rinl | 1.0305028 | GO:0015629 |
| Rinl | 1.0305028 | GO:0005085 |
| Rinl | 1.0305028 | GO:0005096 |
| Rinl | 1.0305028 | GO:0005515 |
| Serpinb9g | 1.1942015 | GO:0010951 |
| Serpinb9g | 1.1942015 | GO:0030162 |
| Serpinb9g | 1.1942015 | GO:0005737 |
| Serpinb9g | 1.1942015 | GO:0004867 |
| Als2cl | 1.0071497 | GO:0007032 |
| Als2cl | 1.0071497 | GO:0008104 |
| Als2cl | 1.0071497 | GO:0035023 |
| Als2cl | 1.0071497 | GO:0016023 |
| Als2cl | 1.0071497 | GO:0005622 |
| Als2cl | 1.0071497 | GO:0005089 |
| Als2cl | 1.0071497 | GO:0005096 |
| Als2cl | 1.0071497 | GO:0005515 |
| Als2cl | 1.0071497 | GO:0042802 |
| Als2cl | 1.0071497 | GO:0017137 |
| Als2cl | 1.0071497 | GO:0007032 |
| Aqp3 | 1.1651936 | GO:0002684 |
| Aqp3 | 1.1651936 | GO:0006833 |
| Aqp3 | 1.1651936 | GO:0015793 |
| Aqp3 | 1.1651936 | GO:0015840 |
| Aqp3 | 1.1651936 | GO:0032526 |
| Aqp3 | 1.1651936 | GO:0042476 |
| Aqp3 | 1.1651936 | GO:0070295 |
| Aqp3 | 1.1651936 | GO:0006810 |
| Aqp3 | 1.1651936 | GO:0006833 |
| Aqp3 | 1.1651936 | GO:0005737 |
| Aqp3 | 1.1651936 | GO:0005887 |
| Aqp3 | 1.1651936 | GO:0005911 |
| Aqp3 | 1.1651936 | GO:0005886 |
| Aqp3 | 1.1651936 | GO:0016323 |
| Aqp3 | 1.1651936 | GO:0016020 |
| Aqp3 | 1.1651936 | GO:0005886 |
| Aqp3 | 1.1651936 | GO:0005911 |
| Aqp3 | 1.1651936 | GO:0015250 |
| Aqp3 | 1.1651936 | GO:0015254 |
| Aqp3 | 1.1651936 | GO:0005215 |
| Aqp3 | 1.1651936 | GO:0015250 |
| Csdc2 | 1.7586055 | GO:0008150 |
| Csdc2 | 1.7586055 | GO:0006397 |
| Csdc2 | 1.7586055 | GO:0006355 |
| Csdc2 | 1.7586055 | GO:0005737 |
| Csdc2 | 1.7586055 | GO:0005634 |
| Csdc2 | 1.7586055 | GO:0003723 |
| Csdc2 | 1.7586055 | GO:0003677 |
| Csdc2 | 1.7586055 | GO:0003676 |
| Fxyd3 | 1.0810175 | GO:0050790 |
| Fxyd3 | 1.0810175 | GO:0006811 |
| Fxyd3 | 1.0810175 | GO:0005789 |
| Fxyd3 | 1.0810175 | GO:0034707 |
| Fxyd3 | 1.0810175 | GO:0016020 |
| Fxyd3 | 1.0810175 | GO:0005216 |
| Fxyd3 | 1.0810175 | GO:0005515 |
| Fxyd3 | 1.0810175 | GO:0051117 |
| Fxyd3 | 1.0810175 | GO:0005254 |
| Carns1 | 1.1703243 | GO:0035499 |
| Carns1 | 1.1703243 | GO:0006200 |
| Carns1 | 1.1703243 | GO:0005575 |
| Carns1 | 1.1703243 | GO:0005524 |
| Carns1 | 1.1703243 | GO:0016887 |
| Carns1 | 1.1703243 | GO:0046872 |
| Carns1 | 1.1703243 | GO:0047730 |
| Cngb1 | 1.2783761 | GO:0008150 |
| Cngb1 | 1.2783761 | GO:0005575 |
| Cngb1 | 1.2783761 | GO:0003674 |
| Mcam | 1.0408211 | GO:0007157 |
| Mcam | 1.0408211 | GO:0007155 |
| Mcam | 1.0408211 | GO:0005886 |
| Mcam | 1.0408211 | GO:0003094 |
| Mcam | 1.0408211 | GO:0061042 |
| Mcam | 1.0408211 | GO:0016021 |
| Mcam | 1.0408211 | GO:0009897 |
| Mcam | 1.0408211 | GO:0005515 |
| Stxbp6 | 1.2740431 | GO:0008150 |
| Stxbp6 | 1.2740431 | GO:0016192 |
| Stxbp6 | 1.2740431 | GO:0005575 |
| Stxbp6 | 1.2740431 | GO:0016021 |
| Stxbp6 | 1.2740431 | GO:0003674 |
| Bend4 | 1.4454508 | GO:0008150 |
| Bend4 | 1.4454508 | GO:0005575 |
| Bend4 | 1.4454508 | GO:0003674 |
| Il17re | 1.004003 | GO:0005737 |
| Il17re | 1.004003 | GO:0005576 |
| Il17re | 1.004003 | GO:0016021 |
| Il17re | 1.004003 | GO:0005515 |
| Plekha4 | 1.0734644 | GO:0008150 |
| Plekha4 | 1.0734644 | GO:0005575 |
| Plekha4 | 1.0734644 | GO:0003674 |
| Plekha4 | 1.0734644 | GO:0005543 |
| Plekha4 | 1.0734644 | GO:0005515 |
| Capn6 | 1.2034225 | GO:0001578 |
| Capn6 | 1.2034225 | GO:0006508 |
| Capn6 | 1.2034225 | GO:0051493 |
| Capn6 | 1.2034225 | GO:0005874 |
| Capn6 | 1.2034225 | GO:0005876 |
| Capn6 | 1.2034225 | GO:0048471 |
| Capn6 | 1.2034225 | GO:0005622 |
| Capn6 | 1.2034225 | GO:0004198 |
| Capn6 | 1.2034225 | GO:0005515 |
| Capn6 | 1.2034225 | GO:0008017 |
| Prph | 1.0329366 | GO:0045104 |
| Prph | 1.0329366 | GO:0005882 |
| Prph | 1.0329366 | GO:0005883 |
| Prph | 1.0329366 | GO:0045098 |
| Prph | 1.0329366 | GO:0044299 |
| Prph | 1.0329366 | GO:0030424 |
| Prph | 1.0329366 | GO:0042622 |
| Prph | 1.0329366 | GO:0043025 |
| Prph | 1.0329366 | GO:0005198 |
| Prph | 1.0329366 | GO:0005515 |
| Aldh1a1 | 1.0613232 | GO:0001822 |
| Aldh1a1 | 1.0613232 | GO:0001889 |
| Aldh1a1 | 1.0613232 | GO:0002072 |
| Aldh1a1 | 1.0613232 | GO:0007494 |
| Aldh1a1 | 1.0613232 | GO:0006979 |
| Aldh1a1 | 1.0613232 | GO:0014070 |
| Aldh1a1 | 1.0613232 | GO:0043065 |
| Aldh1a1 | 1.0613232 | GO:0045471 |
| Aldh1a1 | 1.0613232 | GO:0032355 |
| Aldh1a1 | 1.0613232 | GO:0032526 |
| Aldh1a1 | 1.0613232 | GO:0042904 |
| Aldh1a1 | 1.0613232 | GO:0042493 |
| Aldh1a1 | 1.0613232 | GO:0042572 |
| Aldh1a1 | 1.0613232 | GO:0042573 |
| Aldh1a1 | 1.0613232 | GO:0051289 |
| Aldh1a1 | 1.0613232 | GO:0060206 |
| Aldh1a1 | 1.0613232 | GO:0048048 |
| Aldh1a1 | 1.0613232 | GO:0008218 |
| Aldh1a1 | 1.0613232 | GO:0055114 |
| Aldh1a1 | 1.0613232 | GO:0008152 |
| Aldh1a1 | 1.0613232 | GO:0042905 |
| Aldh1a1 | 1.0613232 | GO:0002138 |
| Aldh1a1 | 1.0613232 | GO:0042493 |
| Aldh1a1 | 1.0613232 | GO:0005737 |
| Aldh1a1 | 1.0613232 | GO:0005634 |
| Aldh1a1 | 1.0613232 | GO:0001758 |
| Aldh1a1 | 1.0613232 | GO:0004028 |
| Aldh1a1 | 1.0613232 | GO:0042802 |
| Aldh1a1 | 1.0613232 | GO:0018479 |
| Aldh1a1 | 1.0613232 | GO:0003995 |
| Aldh1a1 | 1.0613232 | GO:0016491 |
| Aldh1a1 | 1.0613232 | GO:0004029 |
| 9430002A10Rik | -1.2239747 | GO:0008150 |
| 9430002A10Rik | -1.2239747 | GO:0005575 |
| 9430002A10Rik | -1.2239747 | GO:0003674 |
| Pkp2 | 1.0378599 | GO:0007507 |
| Pkp2 | 1.0378599 | GO:0016337 |
| Pkp2 | 1.0378599 | GO:0005911 |
| Pkp2 | 1.0378599 | GO:0005912 |
| Pkp2 | 1.0378599 | GO:0014704 |
| Pkp2 | 1.0378599 | GO:0030057 |
| Pkp2 | 1.0378599 | GO:0005515 |
| Pkp2 | 1.0378599 | GO:0005488 |
| Pkp2 | 1.0378599 | GO:0086091 |
| Pkp2 | 1.0378599 | GO:0055010 |
| Pkp2 | 1.0378599 | GO:0002159 |
| Pkp2 | 1.0378599 | GO:0045110 |
| Pkp2 | 1.0378599 | GO:0086005 |
| Pkp2 | 1.0378599 | GO:0086002 |
| Pkp2 | 1.0378599 | GO:0048496 |
| Pkp2 | 1.0378599 | GO:0086069 |
| Pkp2 | 1.0378599 | GO:0086019 |
| Pkp2 | 1.0378599 | GO:0005882 |
| Pkp2 | 1.0378599 | GO:0005515 |
| Pkp2 | 1.0378599 | GO:0032947 |
| Pkp2 | 1.0378599 | GO:0005080 |
| Pkp2 | 1.0378599 | GO:0019215 |
| Islr | 1.057478 | GO:0005576 |
| Islr | 1.057478 | GO:0005515 |
| Sh3tc1 | 1.185008 | GO:0008150 |
| Sh3tc1 | 1.185008 | GO:0005575 |
| Sh3tc1 | 1.185008 | GO:0003674 |
| Sh3tc1 | 1.185008 | GO:0005515 |
| Cgn | 1.1336632 | GO:0008150 |
| Cgn | 1.1336632 | GO:0016459 |
| Cgn | 1.1336632 | GO:0043296 |
| Cgn | 1.1336632 | GO:0003674 |
| Cgn | 1.1336632 | GO:0003774 |
| Cgn | 1.1336632 | GO:0005515 |
| Tnni1 | 1.0609818 | GO:0006937 |
| Tnni1 | 1.0609818 | GO:0055010 |
| Tnni1 | 1.0609818 | GO:0005861 |
| Tnni1 | 1.0609818 | GO:0003779 |
| Foxs1 | 1.0823464 | GO:0000122 |
| Foxs1 | 1.0823464 | GO:0001503 |
| Foxs1 | 1.0823464 | GO:0001568 |
| Foxs1 | 1.0823464 | GO:0001569 |
| Foxs1 | 1.0823464 | GO:0001570 |
| Foxs1 | 1.0823464 | GO:0001756 |
| Foxs1 | 1.0823464 | GO:0001946 |
| Foxs1 | 1.0823464 | GO:0001974 |
| Foxs1 | 1.0823464 | GO:0007219 |
| Foxs1 | 1.0823464 | GO:0008286 |
| Foxs1 | 1.0823464 | GO:0014034 |
| Foxs1 | 1.0823464 | GO:0043433 |
| Foxs1 | 1.0823464 | GO:0043066 |
| Foxs1 | 1.0823464 | GO:0045892 |
| Foxs1 | 1.0823464 | GO:0030199 |
| Foxs1 | 1.0823464 | GO:0035050 |
| Foxs1 | 1.0823464 | GO:0043010 |
| Foxs1 | 1.0823464 | GO:0040018 |
| Foxs1 | 1.0823464 | GO:0050880 |
| Foxs1 | 1.0823464 | GO:0050885 |
| Foxs1 | 1.0823464 | GO:0055010 |
| Foxs1 | 1.0823464 | GO:0060038 |
| Foxs1 | 1.0823464 | GO:0045944 |
| Foxs1 | 1.0823464 | GO:0046620 |
| Foxs1 | 1.0823464 | GO:0048844 |
| Foxs1 | 1.0823464 | GO:0048343 |
| Foxs1 | 1.0823464 | GO:0048010 |
| Foxs1 | 1.0823464 | GO:0006355 |
| Foxs1 | 1.0823464 | GO:0005634 |
| Foxs1 | 1.0823464 | GO:0005667 |
| Foxs1 | 1.0823464 | GO:0003677 |
| Foxs1 | 1.0823464 | GO:0003705 |
| Foxs1 | 1.0823464 | GO:0003690 |
| Foxs1 | 1.0823464 | GO:0008301 |
| Foxs1 | 1.0823464 | GO:0008134 |
| Foxs1 | 1.0823464 | GO:0043565 |
| Foxs1 | 1.0823464 | GO:0031490 |
| Foxs1 | 1.0823464 | GO:0003700 |
| Best3 | 1.428019 | GO:0015698 |
| Best3 | 1.428019 | GO:0043271 |
| Best3 | 1.428019 | GO:0005886 |
| Best3 | 1.428019 | GO:0034707 |
| Best3 | 1.428019 | GO:0005254 |
| Hes7 | 1.1282716 | GO:0000122 |
| Hes7 | 1.1282716 | GO:0001501 |
| Hes7 | 1.1282716 | GO:0001756 |
| Hes7 | 1.1282716 | GO:0007219 |
| Hes7 | 1.1282716 | GO:0006351 |
| Hes7 | 1.1282716 | GO:0045892 |
| Hes7 | 1.1282716 | GO:0036342 |
| Hes7 | 1.1282716 | GO:0048511 |
| Hes7 | 1.1282716 | GO:0006355 |
| Hes7 | 1.1282716 | GO:0005634 |
| Hes7 | 1.1282716 | GO:0003677 |
| Hes7 | 1.1282716 | GO:0008134 |
| Hes7 | 1.1282716 | GO:0046983 |
| Aloxe3 | 1.2962861 | GO:0019370 |
| Aloxe3 | 1.2962861 | GO:0055114 |
| Aloxe3 | 1.2962861 | GO:0003824 |
| Aloxe3 | 1.2962861 | GO:0005506 |
| Aloxe3 | 1.2962861 | GO:0016165 |
| Aloxe3 | 1.2962861 | GO:0005515 |
| Aloxe3 | 1.2962861 | GO:0016702 |
| Aloxe3 | 1.2962861 | GO:0046872 |
| Ces1a | 1.0534291 | GO:0008152 |
| Ces1a | 1.0534291 | GO:0005575 |
| Ces1a | 1.0534291 | GO:0004091 |
| Ces1a | 1.0534291 | GO:0016787 |
| 1700001C19Rik | 1.1120954 | GO:0008150 |
| 1700001C19Rik | 1.1120954 | GO:0005575 |
| 1700001C19Rik | 1.1120954 | GO:0003674 |
| Slc40a1 | 1.1305532 | GO:0003158 |
| Slc40a1 | 1.1305532 | GO:0002260 |
| Slc40a1 | 1.1305532 | GO:0006826 |
| Slc40a1 | 1.1305532 | GO:0006915 |
| Slc40a1 | 1.1305532 | GO:0006879 |
| Slc40a1 | 1.1305532 | GO:0060586 |
| Slc40a1 | 1.1305532 | GO:0060345 |
| Slc40a1 | 1.1305532 | GO:0048536 |
| Slc40a1 | 1.1305532 | GO:0034755 |
| Slc40a1 | 1.1305532 | GO:0005771 |
| Slc40a1 | 1.1305532 | GO:0005886 |
| Slc40a1 | 1.1305532 | GO:0016021 |
| Slc40a1 | 1.1305532 | GO:0008021 |
| Slc40a1 | 1.1305532 | GO:0005381 |
| Slc40a1 | 1.1305532 | GO:0005515 |
| Slc40a1 | 1.1305532 | GO:0005515 |
| Fer1l6 | 1.8413935 | GO:0008150 |
| Fer1l6 | 1.8413935 | GO:0006810 |
| Fer1l6 | 1.8413935 | GO:0055085 |
| Fer1l6 | 1.8413935 | GO:0005575 |
| Fer1l6 | 1.8413935 | GO:0016021 |
| Fer1l6 | 1.8413935 | GO:0003674 |
| Fer1l6 | 1.8413935 | GO:0005515 |
| Fer1l6 | 1.8413935 | GO:0005524 |
| Fer1l6 | 1.8413935 | GO:0042626 |
| 9930013L23Rik | 1.3005681 | GO:0008150 |
| 9930013L23Rik | 1.3005681 | GO:0005737 |
| 9930013L23Rik | 1.3005681 | GO:0003674 |
| Hap1 | 1.0492382 | GO:0010976 |
| Hap1 | 1.0492382 | GO:0008104 |
| Hap1 | 1.0492382 | GO:0071363 |
| Hap1 | 1.0492382 | GO:0047496 |
| Hap1 | 1.0492382 | GO:0016023 |
| Hap1 | 1.0492382 | GO:0043679 |
| Hap1 | 1.0492382 | GO:0030425 |
| Hap1 | 1.0492382 | GO:0030426 |
| Hap1 | 1.0492382 | GO:0048471 |
| Hap1 | 1.0492382 | GO:0005515 |
| Hap1 | 1.0492382 | GO:0005515 |
| Prss32 | 1.643713 | GO:0006508 |
| Prss32 | 1.643713 | GO:0005886 |
| Prss32 | 1.643713 | GO:0016020 |
| Prss32 | 1.643713 | GO:0004252 |
| Prss32 | 1.643713 | GO:0008236 |
| Prss32 | 1.643713 | GO:0003824 |
| Cyp4f14 | 1.1581664 | GO:0055114 |
| Cyp4f14 | 1.1581664 | GO:0005789 |
| Cyp4f14 | 1.1581664 | GO:0005506 |
| Cyp4f14 | 1.1581664 | GO:0009055 |
| Cyp4f14 | 1.1581664 | GO:0020037 |
| Cyp4f14 | 1.1581664 | GO:0052871 |
| Cyp4f14 | 1.1581664 | GO:0050051 |
| Cyp4f14 | 1.1581664 | GO:0016705 |
| Pi15 | 1.0413632 | GO:0005576 |
| Pi15 | 1.0413632 | GO:0030414 |
| Vill | 1.400919 | GO:0007010 |
| Vill | 1.400919 | GO:0005575 |
| Vill | 1.400919 | GO:0003779 |
| Vill | 1.400919 | GO:0051693 |
| Slc12a1 | 1.300035 | GO:0001822 |
| Slc12a1 | 1.300035 | GO:0007588 |
| Slc12a1 | 1.300035 | GO:0006813 |
| Slc12a1 | 1.300035 | GO:0006814 |
| Slc12a1 | 1.300035 | GO:0048878 |
| Slc12a1 | 1.300035 | GO:0006810 |
| Slc12a1 | 1.300035 | GO:0055085 |
| Slc12a1 | 1.300035 | GO:0006811 |
| Slc12a1 | 1.300035 | GO:0016324 |
| Slc12a1 | 1.300035 | GO:0016021 |
| Slc12a1 | 1.300035 | GO:0016020 |
| Slc12a1 | 1.300035 | GO:0005515 |
| Slc12a1 | 1.300035 | GO:0008511 |
| Slc12a1 | 1.300035 | GO:0015377 |
| Greb1 | 1.7618985 | GO:0016021 |
| Tnfrsf9 | 1.154387 | GO:0042127 |
| Tnfrsf9 | 1.154387 | GO:0009897 |
| Tnfrsf9 | 1.154387 | GO:0004872 |
| Tnfrsf9 | 1.154387 | GO:0005515 |
| Tnfrsf9 | 1.154387 | GO:0016021 |
| Pla1a | 1.0460315 | GO:0016042 |
| Pla1a | 1.0460315 | GO:0008150 |
| Pla1a | 1.0460315 | GO:0005575 |
| Pla1a | 1.0460315 | GO:0005576 |
| Pla1a | 1.0460315 | GO:0003674 |
| Pla1a | 1.0460315 | GO:0016787 |
| Bmf | 1.2751951 | GO:0006915 |
| Bmf | 1.2751951 | GO:0042981 |
| Bmf | 1.2751951 | GO:0032464 |
| Bmf | 1.2751951 | GO:0090200 |
| Bmf | 1.2751951 | GO:0001669 |
| Bmf | 1.2751951 | GO:0005737 |
| Bmf | 1.2751951 | GO:0015629 |
| Bmf | 1.2751951 | GO:0016459 |
| Bmf | 1.2751951 | GO:0005515 |
| Bmf | 1.2751951 | GO:0005515 |
| Enpp3 | 1.101996 | GO:0006796 |
| Enpp3 | 1.101996 | GO:0006955 |
| Enpp3 | 1.101996 | GO:0009143 |
| Enpp3 | 1.101996 | GO:0008152 |
| Enpp3 | 1.101996 | GO:0005576 |
| Enpp3 | 1.101996 | GO:0016021 |
| Enpp3 | 1.101996 | GO:0048471 |
| Enpp3 | 1.101996 | GO:0003676 |
| Enpp3 | 1.101996 | GO:0004528 |
| Enpp3 | 1.101996 | GO:0005044 |
| Enpp3 | 1.101996 | GO:0030247 |
| Enpp3 | 1.101996 | GO:0035529 |
| Enpp3 | 1.101996 | GO:0046872 |
| Enpp3 | 1.101996 | GO:0047429 |
| Enpp3 | 1.101996 | GO:0016787 |
| Enpp3 | 1.101996 | GO:0003824 |
| Adamts13 | 1.3339262 | GO:0007229 |
| Adamts13 | 1.3339262 | GO:0006508 |
| Adamts13 | 1.3339262 | GO:0005615 |
| Adamts13 | 1.3339262 | GO:0005578 |
| Adamts13 | 1.3339262 | GO:0004175 |
| Adamts13 | 1.3339262 | GO:0004222 |
| Adamts13 | 1.3339262 | GO:0008270 |
| Adamts13 | 1.3339262 | GO:0007596 |
| Adamts13 | 1.3339262 | GO:0043171 |
| Adamts13 | 1.3339262 | GO:0034341 |
| Adamts13 | 1.3339262 | GO:0034612 |
| Adamts13 | 1.3339262 | GO:0070670 |
| Adamts13 | 1.3339262 | GO:0006508 |
| Adamts13 | 1.3339262 | GO:0005515 |
| Pappa2 | 1.5146599 | GO:0006508 |
| Pappa2 | 1.5146599 | GO:0030154 |
| Pappa2 | 1.5146599 | GO:0005575 |
| Pappa2 | 1.5146599 | GO:0016020 |
| Pappa2 | 1.5146599 | GO:0008237 |
| Pappa2 | 1.5146599 | GO:0005515 |
| Rab37 | 1.5927901 | GO:0007264 |
| Rab37 | 1.5927901 | GO:0006184 |
| Rab37 | 1.5927901 | GO:0015031 |
| Rab37 | 1.5927901 | GO:0006886 |
| Rab37 | 1.5927901 | GO:0006913 |
| Rab37 | 1.5927901 | GO:0007165 |
| Rab37 | 1.5927901 | GO:0030141 |
| Rab37 | 1.5927901 | GO:0005622 |
| Rab37 | 1.5927901 | GO:0005634 |
| Rab37 | 1.5927901 | GO:0005737 |
| Rab37 | 1.5927901 | GO:0016020 |
| Rab37 | 1.5927901 | GO:0003924 |
| Rab37 | 1.5927901 | GO:0005515 |
| Rab37 | 1.5927901 | GO:0005525 |
| Rab37 | 1.5927901 | GO:0005793 |
| Otop1 | 1.2532458 | GO:0009590 |
| Otop1 | 1.2532458 | GO:0042472 |
| Otop1 | 1.2532458 | GO:0016020 |
| Otop1 | 1.2532458 | GO:0031214 |
| Otop1 | 1.2532458 | GO:0005615 |
| Otop1 | 1.2532458 | GO:0016021 |
| Tdrd9 | 1.1417947 | GO:0007283 |
| Tdrd9 | 1.1417947 | GO:0007275 |
| Tdrd9 | 1.1417947 | GO:0007140 |
| Tdrd9 | 1.1417947 | GO:0009566 |
| Tdrd9 | 1.1417947 | GO:0043046 |
| Tdrd9 | 1.1417947 | GO:0030154 |
| Tdrd9 | 1.1417947 | GO:0031047 |
| Tdrd9 | 1.1417947 | GO:0034587 |
| Tdrd9 | 1.1417947 | GO:0005634 |
| Tdrd9 | 1.1417947 | GO:0071547 |
| Tdrd9 | 1.1417947 | GO:0003676 |
| Tdrd9 | 1.1417947 | GO:0005515 |
| Tdrd9 | 1.1417947 | GO:0005524 |
| Tdrd9 | 1.1417947 | GO:0008026 |
| Tdrd9 | 1.1417947 | GO:0004386 |
| Fmo3 | 1.0424266 | GO:0017144 |
| Fmo3 | 1.0424266 | GO:0055114 |
| Fmo3 | 1.0424266 | GO:0016021 |
| Fmo3 | 1.0424266 | GO:0031227 |
| Fmo3 | 1.0424266 | GO:0043231 |
| Fmo3 | 1.0424266 | GO:0004499 |
| Fmo3 | 1.0424266 | GO:0016597 |
| Fmo3 | 1.0424266 | GO:0034899 |
| Fmo3 | 1.0424266 | GO:0050660 |
| Fmo3 | 1.0424266 | GO:0050661 |
| Fmo3 | 1.0424266 | GO:0016491 |
| Pld4 | 1.2123895 | GO:0016042 |
| Pld4 | 1.2123895 | GO:0008150 |
| Pld4 | 1.2123895 | GO:0008152 |
| Pld4 | 1.2123895 | GO:0005575 |
| Pld4 | 1.2123895 | GO:0005789 |
| Pld4 | 1.2123895 | GO:0016021 |
| Pld4 | 1.2123895 | GO:0004630 |
| Pld4 | 1.2123895 | GO:0005515 |
| Pld4 | 1.2123895 | GO:0070290 |
| Pld4 | 1.2123895 | GO:0003824 |
| Gli1 | 1.5059247 | GO:0007165 |
| Gli1 | 1.5059247 | GO:0007283 |
| Gli1 | 1.5059247 | GO:0007418 |
| Gli1 | 1.5059247 | GO:0007224 |
| Gli1 | 1.5059247 | GO:0009953 |
| Gli1 | 1.5059247 | GO:0009954 |
| Gli1 | 1.5059247 | GO:0030154 |
| Gli1 | 1.5059247 | GO:0030324 |
| Gli1 | 1.5059247 | GO:0021696 |
| Gli1 | 1.5059247 | GO:0021938 |
| Gli1 | 1.5059247 | GO:0021983 |
| Gli1 | 1.5059247 | GO:0060032 |
| Gli1 | 1.5059247 | GO:0045944 |
| Gli1 | 1.5059247 | GO:0045893 |
| Gli1 | 1.5059247 | GO:0045893 |
| Gli1 | 1.5059247 | GO:0008284 |
| Gli1 | 1.5059247 | GO:0090090 |
| Gli1 | 1.5059247 | GO:0045740 |
| Gli1 | 1.5059247 | GO:0009913 |
| Gli1 | 1.5059247 | GO:0001649 |
| Gli1 | 1.5059247 | GO:0045880 |
| Gli1 | 1.5059247 | GO:0005737 |
| Gli1 | 1.5059247 | GO:0005634 |
| Gli1 | 1.5059247 | GO:0005929 |
| Gli1 | 1.5059247 | GO:0005622 |
| Gli1 | 1.5059247 | GO:0005829 |
| Gli1 | 1.5059247 | GO:0003677 |
| Gli1 | 1.5059247 | GO:0003682 |
| Gli1 | 1.5059247 | GO:0003705 |
| Gli1 | 1.5059247 | GO:0005515 |
| Gli1 | 1.5059247 | GO:0008270 |
| Gli1 | 1.5059247 | GO:0008017 |
| Gli1 | 1.5059247 | GO:0005515 |
| Gli1 | 1.5059247 | GO:0044212 |
| Pkp3 | 1.1993194 | GO:0007155 |
| Pkp3 | 1.1993194 | GO:0005634 |
| Pkp3 | 1.1993194 | GO:0005914 |
| Pkp3 | 1.1993194 | GO:0030057 |
| Pkp3 | 1.1993194 | GO:0005515 |
| Pkp3 | 1.1993194 | GO:0005488 |
| Slc1a2 | 1.1141486 | GO:0007399 |
| Slc1a2 | 1.1141486 | GO:0009416 |
| Slc1a2 | 1.1141486 | GO:0010259 |
| Slc1a2 | 1.1141486 | GO:0009611 |
| Slc1a2 | 1.1141486 | GO:0015813 |
| Slc1a2 | 1.1141486 | GO:0007632 |
| Slc1a2 | 1.1141486 | GO:0043200 |
| Slc1a2 | 1.1141486 | GO:0030534 |
| Slc1a2 | 1.1141486 | GO:0031668 |
| Slc1a2 | 1.1141486 | GO:0035264 |
| Slc1a2 | 1.1141486 | GO:0042493 |
| Slc1a2 | 1.1141486 | GO:0021537 |
| Slc1a2 | 1.1141486 | GO:0051938 |
| Slc1a2 | 1.1141486 | GO:0070779 |
| Slc1a2 | 1.1141486 | GO:0046326 |
| Slc1a2 | 1.1141486 | GO:0006835 |
| Slc1a2 | 1.1141486 | GO:0005887 |
| Slc1a2 | 1.1141486 | GO:0043197 |
| Slc1a2 | 1.1141486 | GO:0043198 |
| Slc1a2 | 1.1141486 | GO:0030424 |
| Slc1a2 | 1.1141486 | GO:0030673 |
| Slc1a2 | 1.1141486 | GO:0042734 |
| Slc1a2 | 1.1141486 | GO:0016020 |
| Slc1a2 | 1.1141486 | GO:0005313 |
| Slc1a2 | 1.1141486 | GO:0005314 |
| Slc1a2 | 1.1141486 | GO:0015501 |
| Slc1a2 | 1.1141486 | GO:0017153 |
| Ano9 | 1.0558662 | GO:0008150 |
| Ano9 | 1.0558662 | GO:0005622 |
| Ano9 | 1.0558662 | GO:0034707 |
| Ano9 | 1.0558662 | GO:0003674 |
| Ano9 | 1.0558662 | GO:0005254 |
| Fmnl1 | 1.2466059 | GO:0006929 |
| Fmnl1 | 1.2466059 | GO:0008360 |
| Fmnl1 | 1.2466059 | GO:0009987 |
| Fmnl1 | 1.2466059 | GO:0030866 |
| Fmnl1 | 1.2466059 | GO:0051014 |
| Fmnl1 | 1.2466059 | GO:0016043 |
| Fmnl1 | 1.2466059 | GO:0030036 |
| Fmnl1 | 1.2466059 | GO:0008360 |
| Fmnl1 | 1.2466059 | GO:0030866 |
| Fmnl1 | 1.2466059 | GO:0005829 |
| Fmnl1 | 1.2466059 | GO:0005886 |
| Fmnl1 | 1.2466059 | GO:0045335 |
| Fmnl1 | 1.2466059 | GO:0005522 |
| Fmnl1 | 1.2466059 | GO:0032794 |
| Fmnl1 | 1.2466059 | GO:0051015 |
| Fmnl1 | 1.2466059 | GO:0048365 |
| Fmnl1 | 1.2466059 | GO:0003779 |
| Fmnl1 | 1.2466059 | GO:0017048 |
| Fmnl1 | 1.2466059 | GO:0005488 |
| Fmnl1 | 1.2466059 | GO:0005515 |
| Fmnl1 | 1.2466059 | GO:0032794 |
| Cyb561 | 1.1509414 | GO:0022900 |
| Cyb561 | 1.1509414 | GO:0016021 |
| Cyb561 | 1.1509414 | GO:0030658 |
| Cyb561 | 1.1509414 | GO:0000293 |
| Cyb561 | 1.1509414 | GO:0046872 |
| Serpini1 | 1.025044 | GO:0010951 |
| Serpini1 | 1.025044 | GO:0030155 |
| Serpini1 | 1.025044 | GO:0030162 |
| Serpini1 | 1.025044 | GO:0004867 |
| Serpini1 | 1.025044 | GO:0005576 |
| Serpini1 | 1.025044 | GO:0004867 |
| Cacna1g | 1.5206757 | GO:0001508 |
| Cacna1g | 1.5206757 | GO:0002027 |
| Cacna1g | 1.5206757 | GO:0007268 |
| Cacna1g | 1.5206757 | GO:0010045 |
| Cacna1g | 1.5206757 | GO:0060371 |
| Cacna1g | 1.5206757 | GO:0006811 |
| Cacna1g | 1.5206757 | GO:0055085 |
| Cacna1g | 1.5206757 | GO:0005886 |
| Cacna1g | 1.5206757 | GO:0016021 |
| Cacna1g | 1.5206757 | GO:0016020 |
| Cacna1g | 1.5206757 | GO:0005245 |
| Cacna1g | 1.5206757 | GO:0005515 |
| Cacna1g | 1.5206757 | GO:0008332 |
| Cacna1g | 1.5206757 | GO:0005216 |
| Cacna1g | 1.5206757 | GO:0005891 |
| Cacna1g | 1.5206757 | GO:0042391 |
| Cacna1g | 1.5206757 | GO:0070509 |
| Cacna1g | 1.5206757 | GO:0097110 |
| Cacna1g | 1.5206757 | GO:0008332 |
| Sh2d3c | 1.075047 | GO:0007169 |
| Sh2d3c | 1.075047 | GO:0007165 |
| Sh2d3c | 1.075047 | GO:0007264 |
| Sh2d3c | 1.075047 | GO:0005737 |
| Sh2d3c | 1.075047 | GO:0016020 |
| Sh2d3c | 1.075047 | GO:0005622 |
| Sh2d3c | 1.075047 | GO:0005068 |
| Sh2d3c | 1.075047 | GO:0005085 |
| Sh2d3c | 1.075047 | GO:0005515 |
| Padi2 | 1.3538661 | GO:0018101 |
| Padi2 | 1.3538661 | GO:0005737 |
| Padi2 | 1.3538661 | GO:0004668 |
| Padi2 | 1.3538661 | GO:0005509 |
