# Supplementary material for: Histone deacetylases 1 and 2 maintain S-phase chromatin and DNA replication fork progression
Source: Epigenetics Chromatin. 2013 Aug 15;6:27. doi: 10.1186/1756-8935-6-27 (PMC3765969; doi:10.1186/1756-8935-6-27)
Supplement: Additional file 23: Table S2 — Details of the antibodies used in this study are listed in the supplementary table. [file 1756-8935-6-27-S23.docx]

**Additional File 23: Table S2**

**Antibodies used in this study:**

| **ANTIBODY** | **COMPANY** | **CATALOG** |
| --- | --- | --- |
| Anti-PCNA | Santa Cruz Biotechnology | SC56 |
| Anti-Hdac1 | Abcam | ab19845 |
| Anti-Hdac2 | Abcam | ab32117 |
| Anti-H4K5ac | Abcam | ab51997-100 |
| Anti-H4K12ac | Millipore | 27657 |
| Anti-H4 | Abcam | ab7311 |
| Anti-H3K56ac | Epitomics | 2134-1 |
| Anti-H3K9K14ac | Millipore | 06-599 |
| Anti-βactin | Abcam | ab8224 |
| Anti-BrdU-FITC (FACS) | Molecular probes | B35139 |
| Anti-BrdU (westerns) | BD Biosciences | B555627 |
| Anti-IdU | BD Biosciences | 347580 |
| Anti-CldU | Abcam | ab6326 |
| Anti-PanAc-K | Cell Signaling | 9441 |
| Anti-H3 | Millipore | 05-928 |
| Anti-HP1 gamma | Millipore | MAB3450 |
| Anti-HP1 alpha | Millipore | 05-689 |
| Anti-Smc3 | Cell Signaling Technology | 5696 |
| Anti-H4K16ac | Active Motif | 39167 |
| Anti-SMARCA5 | Abcam | ab3749 |
| Anti-TBP | Abcam | ab818 |
| Anti-RPA | Bethyl Laboratories | A300-244A |
| Anti-P-RPA (S4/S8) | Bethyl Laboratories | A300-245A |
| Anti-Hdac3 | Abcam | ab7030 |
| Anti-Gamma H2AX | Millipore | 05-636 |
| Anti-p53 | Cell Signaling Technology | 92825 |
| Anti-Rad51 | Genetex Inc. | GTX100469 |
